# Surfactant-free room temperature syntheses of stable colloidal silver nanoparticles in alkaline water–alcohol mixtures: pitfalls and opportunities

**DOI:** 10.1039/d6na00408c

**Published:** 2026-08-04

**Authors:** Lukas Høher Sivebæk Lind, Aleksandra Smolska, Javier Eiroa Soto, Jonathan Quinson

**Affiliations:** a Biological and Chemical Engineering Department, Aarhus University 40 Åbogade Aarhus Denmark j.quinson@udc.es; b CICA-Centro Interdisciplinar de Química e Bioloxía, Facultade de Ciencias, Universidade da Coruña, Campus de Elviña 15008 A Coruña Spain

## Abstract

Ethylene glycol in quantities as low as 5 vol% in water with only 0.5 mM NaOH rapidly leads at room temperature to silver nanoparticles. The synthesis is best initiated in the dark to lead to surfactant-free colloidal dispersions that are stable for months. The resulting nanomaterials are relevant for catalysis and beyond.

## Abstract 2

Silver nanoparticles are easily obtained at room temperature without a surfactant in alkaline ethylene glycol and water mixtures.

## Graphical abstract

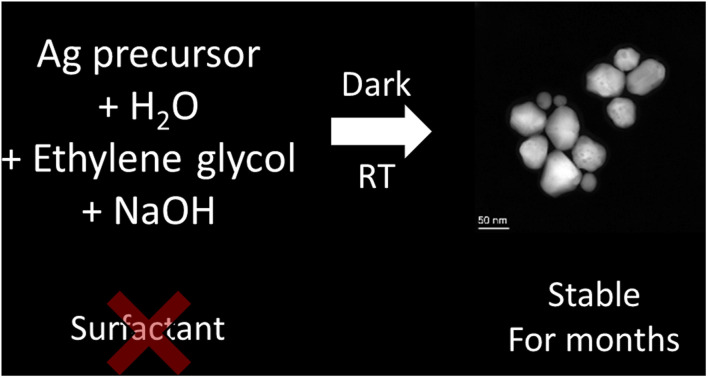

Silver (Ag) nanoparticles (NPs) are among the most studied metal NPs due to their vast range of applications in catalysis, medicine and many more fields.^1–5^ Several syntheses have been reported but often rely on relatively complex processes.^6^ Due to the relatively high redox potential of silver precursors, a range of colloidal syntheses can be performed at room temperature (RT) in water. The process typically requires the use of various additives such as surfactants that can be detrimental for various applications where a clean surface would be needed, for instance, in catalysis of medicine.^7^

To bypass the challenges related to classical colloidal syntheses, we recently reported a very simple synthesis of gold (Au) NPs that only requires water, an alcohol such as ethanol, a base and a metal precursor.^8–11^ The alcohol plays the role of the source of reducing agents, and the NPs are electrostatically stabilized.^12^ The synthesis proceeds easily at RT and qualifies as surfactant-free in the sense that no other chemical than the metal precursor has a molar mass above 100 g mol^−1^, as per the definition discussed in detail elsewhere.^7,13^ Given the general challenges in reproducibility in the nanomaterial sciences and the need to develop simpler and more widely implementable syntheses of NPs,^14–16^ this communication highlights how to best transfer the promising alcohol-mediated synthesis route to the more challenging synthesis of Ag NPs. A broader literature survey, the methodology followed here and the metrics used are provided in the SI. Ag NPs can indeed be obtained as confirmed by UV-vis and STEM (Fig. 1).

**Fig. 1:**
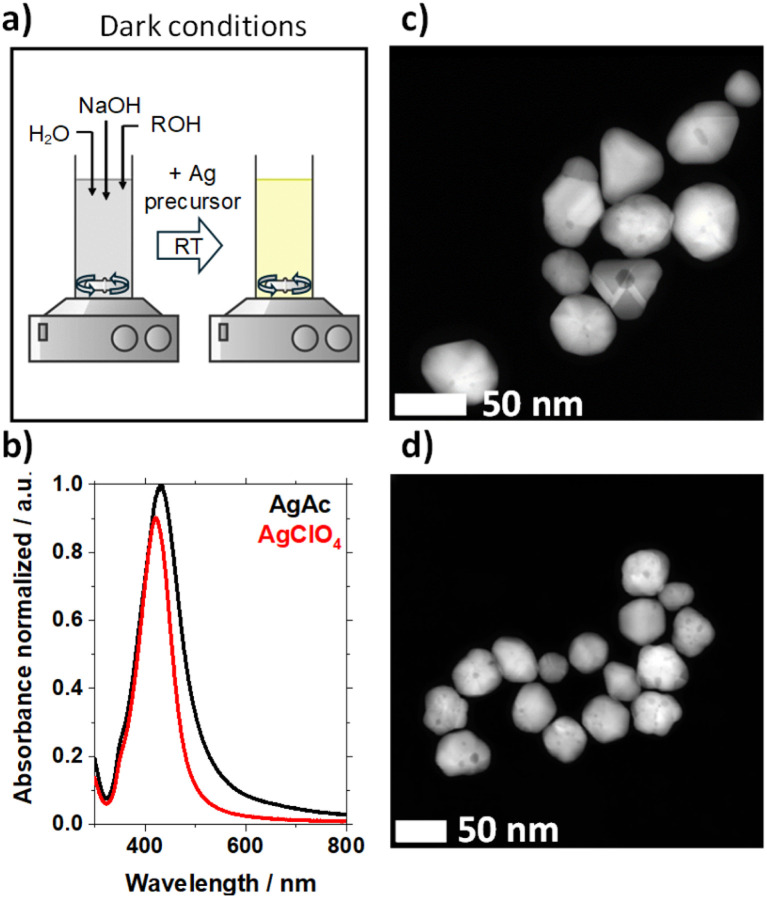
(a) Schematic illustration of the room temperature (RT) synthesis of Ag NPs by a surfactant-free alcohol-mediated colloidal synthesis in alkaline aqueous media. (b) Illustrative UV-vis spectra and (c and d) STEM micrographs of Ag NPs obtained using (c) AgAc or (d) AgClO_4_ as precursors, as indicated. 5 vol% EG, 0.25 mM Ag and a NaOH/Ag molar ratio of 2 were used. In (b), the normalization is performed relative to the maximum of the dataset presented.

However, a first challenge when performing parametric studies on the synthesis of nanomaterials is to ensure that even subtle parameters are actually controlled.^17^ First, we here stress the importance of light in both the synthesis and storage of the Ag NPs. Ag precursors and Ag NPs are photosensitive;^18^ broadly speaking, light is known to influence the formation of Ag NPs, and in particular, the reduction of the precursor as well as Ag NP oxidation.^19,20^ Photo-induced reduction is therefore a possible formation pathway for Ag nanomaterials.^21,22^ However, it is often unclear in most reports, see Table S1, how the synthesis was actually performed, although the preference seems to be for ambient light conditions.^23^ It is also often unclear how the stock solutions and colloids were stored, although there is a preference to use a fridge.^18^

Here, the process is more controlled when the synthesis itself is performed in the dark (as opposed to the case of Au NPs^11^), see Fig. 2, Section S3 and Fig. S5. Stable *A*_spr_ values (*λ*_spr_ in the SI) are observed over time if the synthesis is performed under dark conditions, see Fig. S6–S8. If the synthesis is performed in ambient light, the colloids are less stable over time, regardless of their conditions of storage (ambient temperature or in a fridge). Interestingly, samples stored in the dark and then kept under ambient light conditions also show relatively good stability. The actual reasons for the observed stability have not been established yet but could come from the formation of different chemicals produced depending on how the Ag precursor reduction proceeds under light or dark conditions. Answering this question goes beyond the scope of this first report. Nevertheless, this finding allows performing reliable larger parametric screening at lower energy costs (no need for a fridge).

**Fig. 2:**
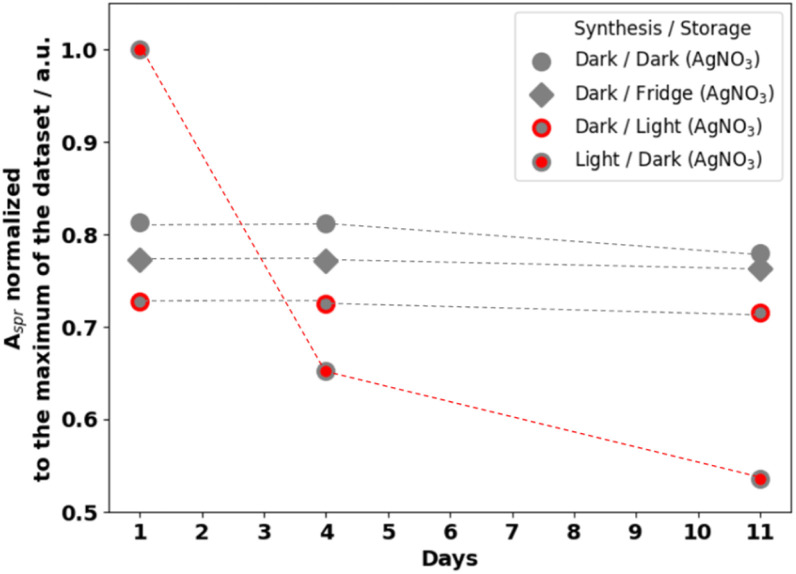
Evolution of *A*_spr_ as a function of time for different synthesis and storage conditions. UV-vis spectra can be found in Fig. S7, and *λ*_spr_ values are presented in Fig. S8. The experiments were performed with 30 vol% EG, 0.25 mM AgNO_3_, and a NaOH/Ag molar ratio of 4. The dotted lines are a guide for the eyes; the red dotted line corresponds to conditions where the synthesis was performed at ambient light, whereas the grey dotted lines correspond to cases where the synthesis was performed under dark conditions.

Having established how to maximize the stability of the surfactant-free colloids, we now turn to the influence of various parameters: nature of alcohol (methanol, ethanol (EtOH), ethylene glycol (EG), and glycerol), alcohol content (5–97.5 vol%, focusing on content lower than 50 vol%), base concentration (NaOH/Ag molar ratio focusing on 2, 4, 8 and 16 for 0.25 mM Ag), type of precursor (AgNO_3_, AgClO_4_, Ag_2_SO_4_, and AgAc), as summarized in Table S2. The influence of each parameter is discussed in Section S4 with further STEM data in Fig. S9–S15 and size analysis in Fig. S16. Bubble plots of *A*_spr_ and *λ*_spr_ values are provided in Fig. S17. Bubble plots of *A*_spr_ values obtained using AgNO_3_ as the precursor are reported in Fig. 3a–c with illustrative UV-vis spectra in Fig. 3d–f (complementary spectra in Fig. S18). A detailed interpretation of the bubble plots is proposed in Section S4.

**Fig. 3:**
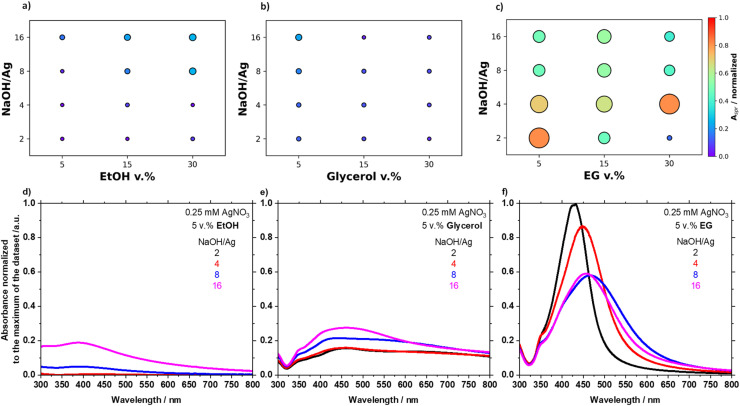
(a–c) Bubble plot of the influence of various parameters on *A*_spr_ values for the synthesis of Ag NPs using different alcohols, different contents of alcohol and different base concentrations (NaOH/Ag molar ratios), as indicated. In all cases, the concentration of Ag was 0.25 mM and the precursor was AgNO_3_. The results for different precursors are reported in Fig. S16 and S17 together with *λ*_spr_ values. The size of the datapoint is proportional to the value they represent, and to help the reading, a colour scale is also used, as indicated. (d–f) Illustrative UV-vis spectra under various experimental conditions, as indicated, using 5 vol% (d) ethanol, (e) glycerol and (f) EG.

EG is found to be the best alcohol to ensure the formation of small size, *ca.* 50 nm, Ag NPs obtained using a surfactant-free procedure at RT, leading to more pronounced spr features and higher *A*_spr_ values. Methanol (not explored further in the study due to higher toxicity, see Table S1) and EtOH lead to poorly defined spectra, indicative of poorly defined NPs, see Section S4. Glycerol leads to slightly better results than EtOH but not as promising as those obtained using EG. Conveniently, the synthesis is easily performed at low EG vol% (5–10 vol%) with a small amount of base (as low as 0.5 mM NaOH). A benefit of using low amount of EG and yet obtained stable colloidal dispersions is to prepare Ag NPs in a relatively low viscosity solvent, which will ultimately facilitate the scale up, *e.g.*, by synthesis in flow systems.^24^

The synthesis is not very sensitive to the nature of the precursor, although we observed a tendency to obtain smaller *λ*_spr_ values when AgClO_4_ was used, indicative of smaller NPs. Using AgNO_3_ or AgClO_4_ as the precursor, size control can be achieved by tuning the NaOH/Ag molar ratio, where larger NPs are obtained at higher NaOH/Ag molar ratios, see Fig. S10. There was no apparent benefit to using higher alcohol contents or higher NaOH/Ag molar ratios, given that those conditions actually led to less defined spectra, suggesting different shapes and/or multimodal populations that were not explored further, see Fig. S18.

The formation of Ag NPs was followed by UV-vis analysis.^25^ The results are presented in Fig. 4, see also Section S5. The time required for the *A*_spr_ values to reach a steady state, indicative of a completed reaction, increases in the order of EtOH < glycerol < EG. It takes maximum *ca.* 20 min to complete the reaction. The classical nucleation theory would suggest that a fast reduction and nucleation would lead to more monodispersed NPs.^26^ In this sense, it could be expected that the conditions leading to the fastest reduction would favour more defined NPs. It is actually observed that the slowest formation in EG actually leads to the Ag NPs with the best defined size and optical properties. This might in part be due to the absence of surfactant that in most cases play a dual role of reducing agent and stabilizers in more conventional colloidal syntheses.^27,28^ Overall, the results show the promising features of this new and relatively simple synthesis to ultimately shine new light on the formation mechanism of Ag NPs.^13^

**Fig. 4:**
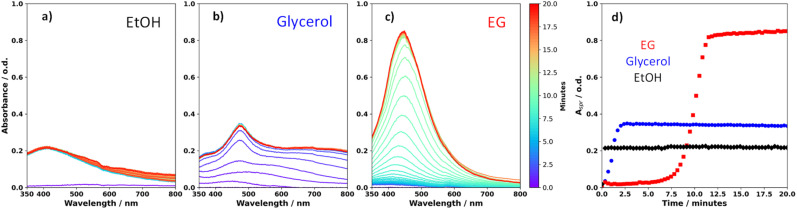
Illustrative UV-vis kinetics of Ag NP formation using (a) EtOH, (b) glycerol and (c) EG. In all cases, the amount of alcohol was 30 vol%, the base concentration was 1 mM and the precursor was AgNO_3_ at 0.94 mM. (d) Evolution of *A*_spr_ as a function of time for every alcohol, as indicated.

Despite the absence of surfactants, long-term storage over months at RT is possible without the need for a fridge, provided the synthesis itself is performed in the dark, *e.g.*, for as long as 5 months, see Section S6 and Fig. S19. The stabilization is attributed to electrostatic interactions confirmed by zeta-potential measurements with a value around −20 to – 40 mV, indicative of stable colloidal dispersions in a solution with a final pH typically around 5. The surfactant-free Ag NPs are promising catalysts, for example, for water treatment as detailed in the SI, see Section S7 and Fig. S20.

## Conclusions

In conclusion, we report a surfactant-free RT synthesis concept of Ag NPs in alkaline water–EG mixtures, at low EG content (5 vol%) and low base concentration (0.5 mM), leading to NPs that are stable for months at RT, provided the synthesis is performed in the dark. While there is certainly room for improvement and to provide a detailed understanding of the role of EG and the stabilization mechanism of the NPs, the synthesis approach reported combines the promising features of being fast, simple and widely implementable. The synthesis approach developed bears the potential for high throughput to provide deeper insights into the formation and stabilization mechanisms of the Ag NPs that will ultimately be required to achieve finer size control^11,29,30^ to make the most of their properties, for instance, in catalysis.^5,31^

## Author contributions

Conceptualization: J. Q.; data curation: L. H. S. L., A. S., and J. Q.; formal analysis: L. H. S. L. and A. S.; funding acquisition: J. Q.; investigation: L. H. S. L. and A. S.; methodology: L. H. S. L., A. S., and J. Q.; project administration: J. Q. ; resources: J. Q.; supervision: J. Q.; validation: L. H. S. L.; visualization: L. H. S. L., A. S., and J. Q.; writing – original draft: J. Q.; writing – review and editing: L. H. S. L., A. S., and J. Q.

## Conflicts of interest

There are no conflicts to declare.

## Supplementary Material

NA-OLF-D6NA00408C-s001

## Data Availability

The data supporting this article have been included as part of the supplementary information (SI). Supplementary information: further literature; methodology; UV-vis, STEM, XRD and catalysis data. See DOI: https://doi.org/10.1039/d6na00408c.
