# Supplementary material for: Surfactant-free room temperature syntheses of stable colloidal silver nanoparticles in alkaline water–alcohol mixtures: pitfalls and opportunities
Source: Nanoscale Adv. 2026 Aug 4;8(17):4781–4. doi: 10.1039/d6na00408c (PMC13474992; doi:10.1039/d6na00408c)
Supplement: Supplementary file 1 [file NA-008-D6NA00408C-s001.pdf]

Supplementary Information (SI) for Nanoscale Advances.
This journal is © The Royal Society of Chemistry 2026

Supplementary information for

Surfactant-free room temperature syntheses of stable colloidal silver nanoparticles in alkaline water-alcohols mixtures: pitfalls and opportunities

Lukas Høher Sivebæk Lind,[a] Aleksandra Smolka,[a] Javier Eiroa Soto,[b] Jonathan Quinson[b,a,*]

a) Biological and Chemical Engineering Department, Aarhus University, 40 Åbogade, Aarhus, Denmark

b) CICA-Centro Interdisciplinar de Química e Bioloxía, Facultade de Ciencias, Universidade da Coruña, Campus de Elviña, 15008, A Coruña, Spain

E-mail: jquinson@bce.au.dk, j.quinson@udc.es

Table of content

S1. Literature 2
S2. Materials and Methods 5
S3. Influence of light 17
3.1. Influence of light on the synthesis 17
3.2. Influence of light on storage 20
S4. Parametric study 22
4.1. Further STEM data 23
4.2. Further UV-vis data 29
4.3. Influence of alcohol 33
4.4. Influence of alcohol content 33
4.5. Influence of NaOH/Ag molar ratio 34
4.6. Influence of precursors 34
4.7. Influence of temperature 34
4.8. Comments on size and shape control 35
4.9. Conclusion 35
S5. Kinetics 36
S6. Stability 37
S7. Proof of concept for catalysis 38
S8. Comparison gold *vs.* silver nanoparticles 39
S9. References 40

S1. Literature

Colloidal syntheses. Colloidal syntheses are common in academia to obtain various (metal) nanomaterials (NMs) in the liquid phase.[1] In a typical experiment, a metal precursor containing at least one metal atom in an oxidized state is reduced in a solvent, typically after energy is provided, in presence of a reducing agent and typically in presence of stabilizers.[2, 3]

Challenges in colloidal syntheses. The classical syntheses of NMs using given solvents, reducing agents and stabilizers, remain challenging to fully understand and control due to several factors: (**i**) the inherent complex chemistry at stake in the formation of NPs and the sensitivity to several seemingly irrelevant parameters, such as the importance of clean glassware,[4] influence of lights,[5] influence of the type of containers used for storage of the stock solutions,[6] influence of chemical grade,[7] of the age of stock solution,[6, 8] influence of the type of precursor,[9] influence of the order of addition of the chemicals,[5] etc.; (**ii**) the challenging and time consuming exploration and characterization of the vast parameter space available to tune a given NP properties;[10] (**iii**) the trend to not report 'failed' experiments which prevent researchers to better design their studies.[11]

As a consequence, the remaining drawbacks to develop more sustainable colloidal syntheses are to develop approaches using: (**A**) safe(r) chemicals, (**B**) few(er) chemicals, (**C**) little amount of a given chemical (other than the precursor), (**D**) well-identified chemicals, (**E**) little energy. Point (D) is to be opposed to biogenic methods using for instance plant extracts or micro-organisms that play the role of reducing agent and/or stabilizers and where the active species can be challenging to identify, and that are largely reported for their potential, yet arguable, *green* features,[12] as commented below and elsewhere.[11]

Au and Ag NPs. A holistic approach towards sustainability requires that sustainable, safe, and simple(r) syntheses of the relevant nanomaterials are developed.[13-15] Conveniently, Au and Ag NPs, that are both important materials for various applications for instance in catalysis, can easily be obtained by colloidal approaches using mild reducing agents, due to the high redox potential of the metal complex precursors required.[4] In particular, a range a biogenic methods using plant extracts or micro-organisms have been reported.[16] Unfortunately, these syntheses require extraction steps in typically organic media and will depend on the batch and source of the plants and/or organisms used. These chemicals can play the role of reducing agents and/or stabilizers, which overall make the controlled synthesis of Au or Ag NPs not as *green* as expected in the first place,[17] and potentially challenging to control and reproduce in diverse parts of the world and/or to scale.

Alcohol-mediated syntheses. Among promising strategy to possibly develop green(er) syntheses of Ag NPs and address part of the challenges above is the use of alcohols as solvent and source of reducing agents, as milder chemicals than other reactants such as $NaBH_4$ commonly used in aqueous room temperature (RT) syntheses of Ag NPs.[18] An account for various reported using alcohols as solvent / reducing agent is proposed in **Table S1**. Ag NPs have previously been obtained using mono-alcohols. Examples of alcohols reported are tert-butanol,[19] isopropanol[20] or

ethanol.[21] However, the synthesis is induced radiolytically[19] or using high temperatures.[22, 23] Promisingly, to develop low energy processes, the synthesis can be performed at RT in the presence of a base.[20] However, stabilizers are used almost systematically.[21] Polyols are also widely reported for the synthesis of various materials,[24] and Ag NPs is not an exception. In particular, glycerol has been extensively studied,[25] and to a lower extend ethylene glycol (EG)[26] has been proposed as an alternative. It has been suggested that aldehydes formed from the alcohols account for the ability to reduce Ag precursor in the case of glycerol[8], e.g. glycolaldehyde for EG-mediated synthesis, when the reaction is not performed under alkaline conditions. Without base the synthesis is usually slow and proceeds in several days in glycerol at RT.[27, 28] Under alkaline conditions the synthesis can proceeds at RT,[29] mediated by the formed alkoxides.[30] In almost all cases, the studies focus on using 100% alcohol and/or a stabilizer is added,[10] for instance PVP.[7] Avoiding these additives, that can also play multiple roles such as reducing agents *and* stabilizers, could be beneficial because their purity can (negatively) affect the results of the synthesis,[7, 29, 31] and for catalysis the removal of those additives is often required to lead to more active nano-catalysts.[32]

Overall, there is a tendency to prefer $AgNO_3$ and $AgClO_4$ as precursors for Ag NPs syntheses in alcohols-mediated syntheses, a large focus is given to 100% alcohol, with a preponderance for using additives. There is also often little information on the conditions under which the reaction where performed (e.g. which light conditions), despite the reported photosensitivity of Ag based precursors and NPs.[33, 34] A final consideration to develop more sustainable investigation, in particular in the case of designing comprehensive studies, is that usually rather large volumes of solutions (5 mL ore more are typically used), as summarized in **Table S1**.

Surfactant-free syntheses in alkaline water-alcohol mixtures. Although they are challenging to develop, *surfactant-free* syntheses of colloidal nanomaterials are promising since they minimize the amount of (toxic) chemicals to use, which can improve reproducibility,[7] and lead to readily active materials for catalysis,[35] without generating extra waste (e.g. for surfactant removal) and without requiring high energy steps.[36] These syntheses will ultimately be ideal model systems to provide a better understanding of nanomaterials formation.[37]

To develop simpler yet robust *green* synthesis of nanomaterials, we reported a synthesis of gold (Au) NPs performed simply performed in water-alcohols mixtures at RT easily leading to ca. 10 nm Au NPs.[5, 38] The synthesis presents the benefits to only require a base such as NaOH,[5] commercially available mono-alcohol such as ethanol,[39] high purity water,[39] and a metal precursor. No extra additives / ligands / surfactant / stabilizers are needed and therefore the synthesis can be considered *surfactant-free*, following a definition of surfactant-free NPs that focus on the synthesis rather than the final nanomaterials as detailed in a dedicated review.[40] The benefits of surfactant-free synthesis is to require fewer chemicals and when performed in low boiling point solvents can lead to readily active catalysts.[36]

Table S1. Overview of the literature on Ag NPs with a focus on synthesis using alcohols (ROH) as solvent and/or source of reducing agents.

Ref	Date	Gas	Precursor		ROH		[NaOH] mM	V mL	Size nm	Additives	Method	Light conditions		
			Type	[Ag] mM	Type	v.%						Synthesis	NP Storage	Stock Silver
20	1993	Ar<br>O_2	$AgNO_3$	0.25	2-propanol	50, 100	0.5	100	7.4	Nafion	RT	-	-	Fresh<br>or Dark
19	2005	N_2	$AgClO_4$	0.10	tert-butanol	100	-	-	20	PVP	Radiolysis	N/A	-	Fresh<br>+Dark
22	2009	-	$AgNO_3$	2.00	Ethanol	100	-	10.2	10 ± 5	PVP	Microwave	N/A	-	-
41	2021	-	$AgNO_3$	5.00	Ethanol	100	-	100	5-30	NaOleate	50-80 °C	-	-	-
23	2002	-	$AgNO_3$	100+	EG	100	-	11	100+	PVP	160 °C	-	-	-
28	2005	-	$AgNO_3$	22.00	EG	100	-	50	10	PVP	RT	-	-	-
25	2007	N_2	$AgClO_4$	0.10	EG	1-99	-	-	20-70	-	Radiolysis	N/A	-	-
31	2020	Ar	$AgOCOCF_3$	0.03	EG	100	-	5	16-28	TA,NaCt<br>PVP	150 °C	-	Fridge	Freshly<br>prepared
29	2010	-	$AgNO_3$<br>$AgClO_4$	0.10	EG<br>Glycerol	100	5.0	-	-<br>25<br>25	-<br>SDS<br>PVP	RT	-	-	Freshly<br>prepared
42<br>30	2012<br>2015	-	$AgNO_3$	0.05<br>0.40	Glycerol	3.7	100<br>10	10	10-20	PVP	RT	-	-	-
43	2014	-	$AgNO_3$	0.10	Glycerol	0.7	10.0	10	20	PVP	RT	-	-	-
8<br>27	2020<br>2021	-	$AgNO_3$	0.10	Glycerol	100<br>10-30	-	5	38 ± 13<br>10-70	-<br>PVP	RT<br>Light<br>Ultrasound<br>Heat	-	-	(Freshly<br>prepared)
44	2023	-	$AgNO_3$	-	Glycerol	10-100	-	-	1-170	PVP	RT	-	-	-
**This work**	2026	-	$AgNO_3$<br>$AgClO_4$<br>Ag_2SO_4<br>Ag(Ac)	0.25	Methanol<br>Ethanol<br>EG<br>Glycerol	5-97.5	0.5-4.0	2	30-100	-	RT	Dark	Dark	Fridge

NaCt: sodium citrate; PVP: polyvinylpyrrolidone; SDS: sodium dodecyl sulfate; TA: tannic acid

S2. Materials and Methods

Chemicals. All chemicals were used as received: silver nitrate ($AgNO_3$, ACS reagent, ≥ 99.0 %, Sigma Aldrich); silver perchlorate ($AgClO_4$, monohydrate, ≥ 98.0%, VWR); silver sulfate (Ag_2SO_4, ACS reagent ≥ 99.5 %, Sigma Aldrich); silver acetate ($AgC_2H_3O_2$, AgAc, ReagentPlus, 99%, Sigma Aldrich); methanol (AnalaR NORMAPUR, Reag. Ph. Eur. ACS, VWR); ethanol (EtOH, Absolute, VWR); ethylene glycol (EG, EMSURE®, Reag. Ph. Eur. Reag. USP, Supelco®); glycerol (bi-distilled 99.5%, VWR); NaOH (Reag. Ph. Eur., VWR); water (mQ, Milli-Q,Millipore, resistivity of >18.2 MΩ•cm); HCl (puriss. ACS reagent, reag. ISO, Reag. Ph. Eur. fuming, ≥ 37%, Sigma Aldrich); HNO_3 (puriss ≥ 65%, Sigma Aldrich); 4-nitrophenol (4-NP, ReagentPlus®, ≥99%, Sigma-Aldrich); $NaBH_4$ (ReagentPlus®, 99%, Sigma-Aldrich).

Note on Sustainable Chemistry. Before considering the various variables that can influence the NP properties, an important conceptual consideration is that developing *green(er)* syntheses of NPs also requires developing *green(er)* investigations.[15] Developing (i) time and (ii) resource efficient studies is a priority to achieve sustainable goals and research. Here, a focus is given on exploiting RT processes and the screening is performed in 2 mL, i.e. a relatively low volume to minimize the amount of chemicals needed and waste generated. The reaction is directly performed in polystyrene UV-vis cuvette for convenient labelling, storage and UV-vis characterization. An inherent benefit of this synthetic strategy is to allow the screening of a relatively large parameter spaces. Furthermore, the effect of N_2 (absence of O_2) was not investigated at this stage to keep the synthesis protocol as simple as possible.

General synthesis. The general synthesis method was adapted from a method developed for Au NPs reported by Quinson et al.[5, 38, 39] Stock solutions of $AgNO_3$, $AgClO_4$, Ag_2SO_4 and AgAc in mQ were prepared at 50, 50, 25 (to use the same volume of stock solutions to obtain a given quantity of Ag) and 50 mM, respectively, in glass containers. All solid powders were handled with plastic spatula. These stock solutions were covered with aluminum when in use and stored in a fridge at ca. 5 °C. Stock solution of the precursors were all prepared using mQ and alcohol used as received (stored at RT in a chemical cabinet) and stored in glass (Pyrex® containers). A stock solution of NaOH at 50 mM in mQ was used (stored at RT in a draw, in plastic container[6]). The EG and glycerol were used as received but due to their high viscosity, the as received solutions were preferentially diluted to 60 v.% in mQ to ensure more reproducible results and this solution was used as a preferred stock solution (both stored at RT in a plastic container and in chemical cabinet).

A UV-vis rectangular cuvette made of polystyrene (1 cm wide, square shape) was used as reactor. A Teflon-covered magnet was placed in the cuvette (8 mm x 3 mm). The magnets were cleaned with *aqua regia* (4:1; v:v; HCl:HNO_3 – ***to be handled and discarded with care following the good practices implemented in the laboratory***), copiously washed with water and dry before use. The reactor and magnets were de-dusted with a flow of compressed air before chemicals were added. The water, base and alcohol stock solutions were mixed in the desired ratios and the stock solution of Ag precursor was added last under stirring at 500 rotations per minutes (rpm). Note that the magnetic field of most stirring device is strong enough so

that up to ca. 25 samples ca be stirred at once. The reactor when the capped and the sample left to stir for 2 hours (or as indicated) in a Photobox to control the light conditions[39] (ultimately used as a black box without light on, or as indicated, Puluz LED portable photo studio, PU5060EU, 60 cm x 60 cm x 60, 60 W). Afterwards the magnets were removed and the samples were stored at RT away from the light (or as indicated). In all cases the final volume was 2.0 mL. In a typical experiments the concentration of Ag was 0.25 mM. **Table S2** provides an overview of the parameter space explored. The general procedure is schematized in **Figure 1** of the manuscript. As detailed in the manuscript, the synthesis is best performed under dark conditions (using aluminum foil when and after the Ag precursor is added, and/or using a photobox without light on to minimize the light exposure during addition of the Ag precursor).

Given the influence of the light and storage conditions established below, a more extensive parametric studies was then performed using 2 hours stirring under dark conditions (obtained using aluminum foil) before samples were stored at RT still in the dark (aluminum foil).

Table S2. Overview of the overall parameter space screened in this study for the surfactant-free synthesis of Ag NPs in alkaline water-alcohols mixtures.

<table>
<tr><th>Alcohol (ROH)</th><th>v.%</th><th>Precursor</th><th>[Ag] mM</th><th>Volume mL</th><th>NaOH/Ag Molar ratio</th><th>Light conditions for synthesis</th><th>Storage</th></tr>
<tr><td>Methanol</td><td>30</td><td>$AgNO_3$
$AgClO_4$</td><td rowspan="4">0.25</td><td rowspan="4">2</td><td>4</td><td>Light</td><td>RT</td></tr>
<tr><td>Ethanol</td><td>5-97.5</td><td rowspan="3">$AgNO_3$
$AgClO_4$
Ag_2SO_4
Ag(Ac)</td><td>2-16</td><td>Light / Dark</td><td>RT</td></tr>
<tr><td>EG</td><td>5-97.5</td><td>2-16</td><td>Light / Dark</td><td>RT / 5 °C</td></tr>
<tr><td>Glycerol</td><td>5-30</td><td>2-16</td><td>Dark</td><td>RT</td></tr>
</table>

Note that the parameter space investigated is limited by the fact that without alcohol the reaction does not proceed (no source of reducing agent), and without base the reaction is poorly controlled (no formation of alkoxides that will serve as reducing agents). We also aimed to use the same stock solutions of Ag precursor and base prepared in mQ water and therefore did not explore 100% alcohols. There was not clear benefits to use alcohol contents above 50 v.% and higher based contents so the focus below is on low v.% of alcohols and low amounts of base. In a same way, due to the ideal use of dark conditions and the benefits of RT processes, no incentive was given to study in detail the effect of temperature at this stage.

Kinetics. Kinetics experiments were performed by measuring the UV-vis spectra of 2 mL solution in the same cuvettes as detailed above (see experimental details below). Due to saturation of the equipment used for measurements, the samples were prepared from the stock solutions of silver detailed above diluted by 4 to lead to new stock solutions. The final concentrations used were 0.94 mM of Ag, 1 mM of NaOH, and 30 v.% alcohol (therefore a NaOH/Ag molar ratio of 1).

Catalysis. The catalytic tests was performed following our recently reported procedure,[45] and following the general guidelines from the literature.[46] ***Pay attention on how to discard the $NaBH_4$ and 4-nitrophenol based waste following the good practices implemented in the laboratory***. Briefly, 30 µL of as prepared 0.25 mM Ag NPs were added to 0.1 mL of 4-nitrophenol at 1 mM mixed with 0.1 mL of of $NaBH_4$ at 100 mM. Total volume of the aqueous solution was 2 mL. This leads to a final concentration of 0.00375 mM of Ag, 0.05 mM of 4-nitrophenol and 5 mM of NaBH4, therefore a final $NaBH_4$/4-nitrophenol molar ratio of 100 and a Ag/4-nitrophenol molar percentage of 7.5%. The decrease of the absorbance at 400 nm was followed over time using a Go Direct® UV-vis spectrophotometer.

Characterization

UV-vis. General UV-vis measurements were performed using a Scientific Genesys 10S UV-vis spectrophotometer with a scanning range of 290 nm to 800 nm. For the kinetics, a GO Direct UV-VIS Spectrophotometer (measuring the range 220-850 nm in a few seconds) was used. In all cases, a background consisting of water-alcohol mixture with the relevant alcohol and alcohol v.% was used. The samples were measured as prepared. Unless otherwise specified, all samples were systematically characterized after 24 hours, and longer time for time studies, as indicated.

STEM. Scanning transmission electron microscopy (STEM) micrographs were acquired on a TALOS F200X operated at 200 kV equipped with a high-angle annular dark-field (HAADF) detector. The samples were prepared by depositing the as-prepared colloids on Cu TEM grids. The Ag NP diameters were determined using ImageJ counting at least 50 NPs and typically more than 50 NPs, as indicated.

XRD. X-ray diffraction (XRD) measurements were acquired using a Panalytical Aeris diffractometer configured in Bragg-Brentano geometry equipped with a PIXcel1D detector. The instrument employed Cu Kα radiation (λ_1 = 1.54056 Å, λ_2 = 1.54439 Å), operated at 40 kV and 15 mA. The diffraction pattern was collected over a 2θ range of 5-90°, with an acquisition time of 600 min.

For sample preparation, 1 mL of Ag NPs dispersion was centrifuged at 12000 rpm for 10 minutes. The supernatant was discarded, and the resulting precipitate was washed three times with ethanol. The purified material was then redispersed in 50 µL of ethanol via sonication and drop-casted onto an XRD sample holder. The deposited material was dried at 40°C in the oven to remove residual solvent before analysis.

Zeta-potential measurements. Zeta potential measurements were performed using a Zetasizer Pro (Malvern Panalytical) equipment that uses an advanced electrophoretic light scattering technique (M3-PALS with Constant Current Mode). The solutions were measured in a dedicated cell (Disposable Folded Capillary Cell, DTS1070). The parameters used for acquisition were a refractive index of silver NPs of 0.06 and an absorption of 4.276.

pH. pH measurements were performed using a Sigma-Aldrich® micro pH combination electrode, glass body after calibration of a pH probe using pH buffers of pH values 10, 7, and 4.

Methodology on characterization. Although a range of characterization are typically used for NP characterization,[47] a detailed characterization of all samples is impossible for the ca. 270 samples considered here (too time-, resource- and cost- consuming). Conveniently, Ag NPs show size dependent surface plasmon resonance (spr) that can be studied in UV-vis, i.e. by a relatively simple and high throughput technique.[48] In practice the spr phenomenon and related values depend on complex materials-size-shape-medium parameters and the relationships detailed below are only true in first approximation, e.g. the values retrieved are solvent-dependent, so in theory no comparison between the use of different alcohol can be performed. However, given the low amount of alcohol used here, UV-vis remains a convenient guide for screening studies.

The position of the spr (λ_{spr}) relates to the size of the Ag NPs, where lower λ_{spr} correspond to smaller size NPs.[28, 48] The absorbance intensity of the spr (A_{spr}) can be related to the formation of monodisperse Ag NPs: in first approximation a higher A_{spr} is proportional of the number of Ag NPs and increases as the NPs get more mono-dispersed and/or smaller for NPs in the size range 30-100 nm.[28, 49] These two parameters are retained for evaluating the properties of the colloids. The evolution of the spectrum is also giving an indication on the stability of the colloids, where stable A_{spr} and λ_{spr} values over time correspond to more stable NPs. Only selected samples (based on the UV-vis data) were then characterized by STEM for a more detailed size and shape analysis. Results in **Figure S1** confirm that higher A_{spr} and lower λ_{spr} correspond to smaller size NPs in agreement with previous reports.[28, 49] See also **Figure S2** and **Table S3**.

It is important to stress that the UV-vis data are more descriptive of the samples given that the signal results from the overall sample whereas STEM data takes into account only few and selected NPs.

a)

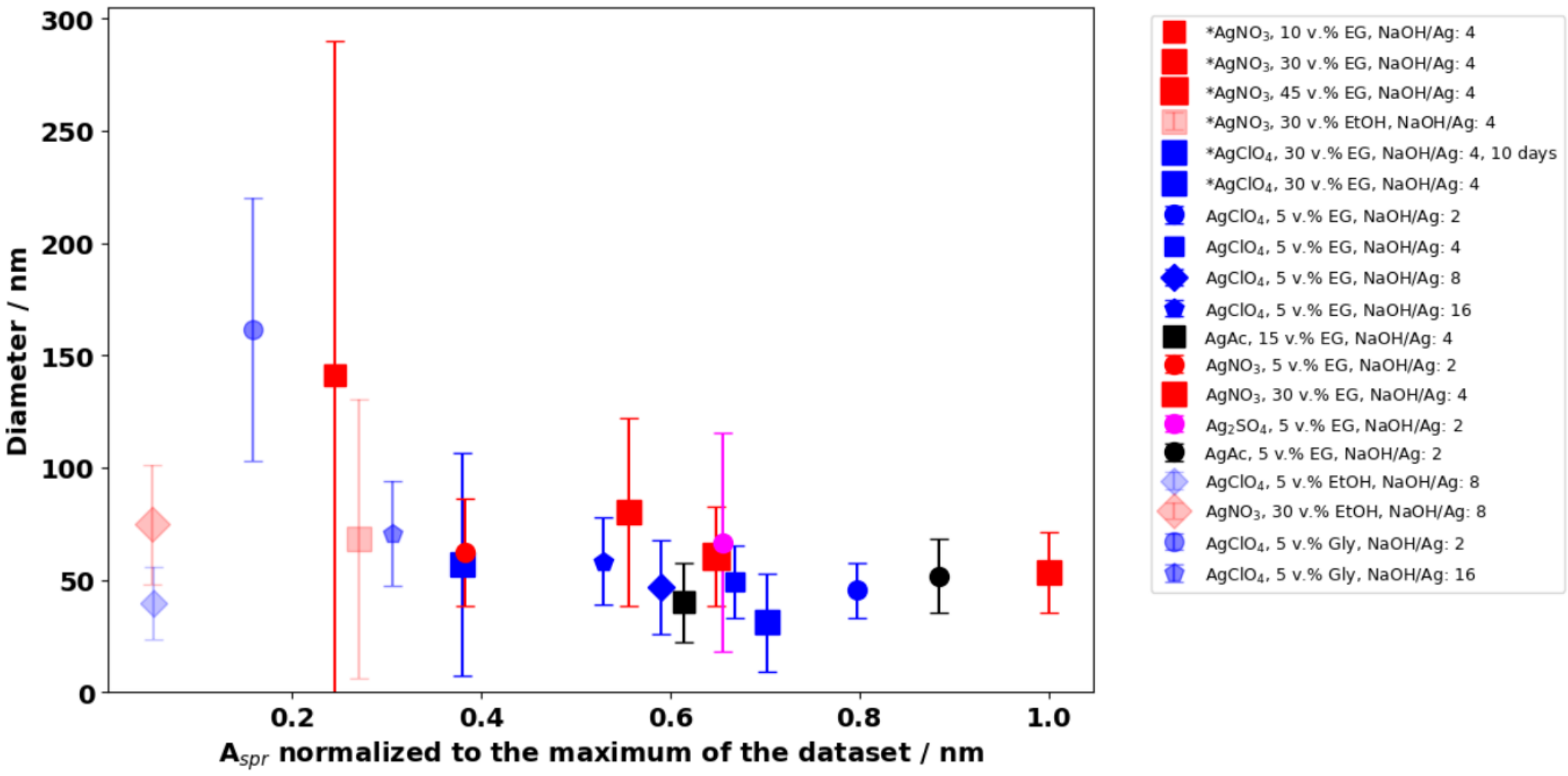

b)

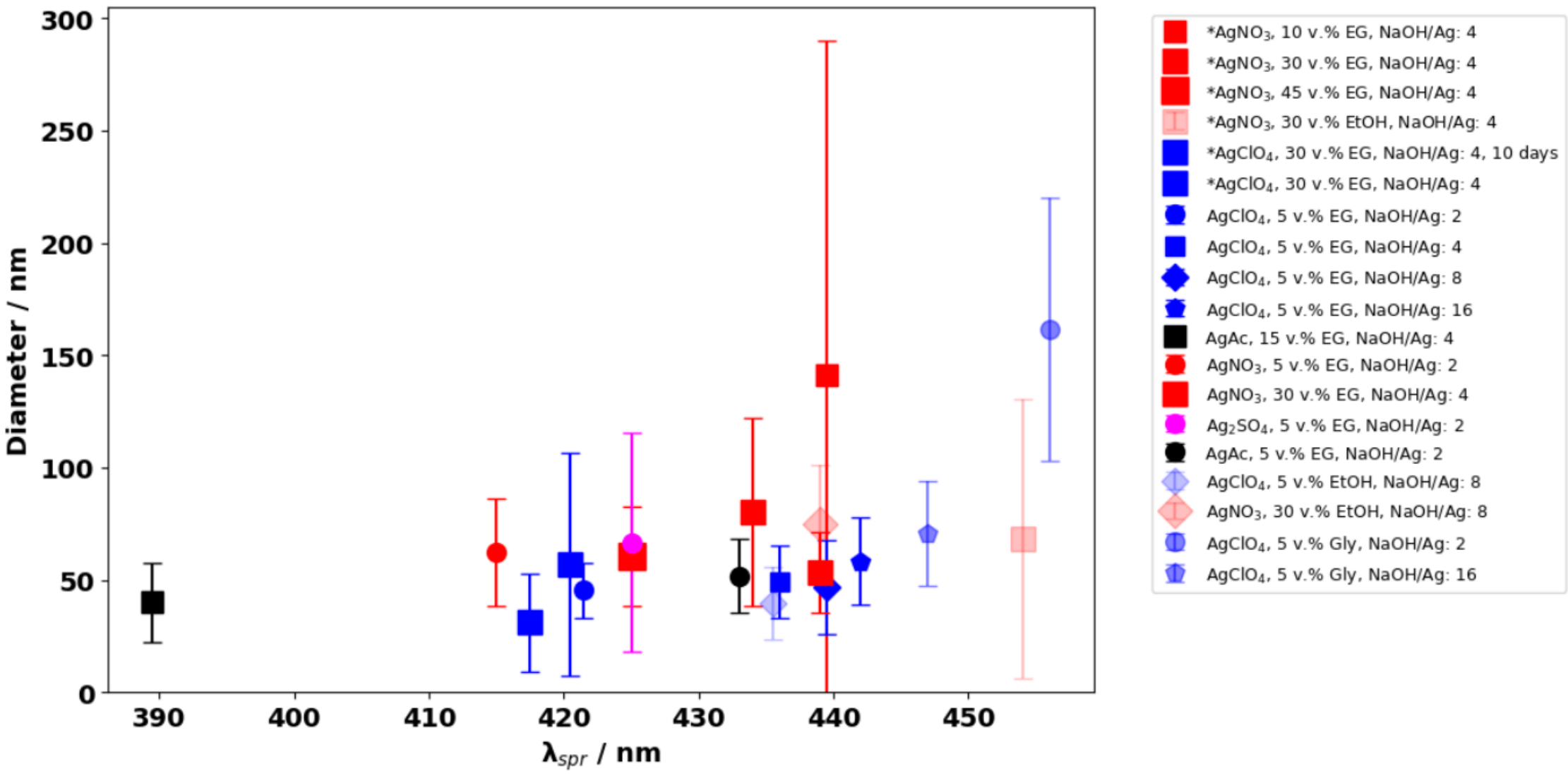

Figure S1. Size distribution and standard deviation as a function (a) A_{spr} and (b) λ_{spr} values for various metal precursors, alcohols and alcohol contents, as indicated. The colors represent the different metal precursors $AgNO_3$ (blue), $AgClO_4$ (red), Ag_2SO_4 (magenta), and AgAc (black). Alcohols are denoted with distinct shades. The NaOH/Ag is coded by the shape of the datapoints and the amount of alcohol by the size of the datapoint. A *star* (*) before the name indicates that the synthesis was run at ambient light condition. *10 days* means that the sample was 10 days old when the grid was prepared.

Table S3. Relationship between metrics retrieved from UV-vis and STEM analysis for specific samples.

Entry	Conditions synthesis	Precursor	Alcohol	Alcohol v.%	NaOH/Ag molar ratio	A_{spr}*	λ_{spr} nm	d nm	N
1	Light	$AgNO_3$	EG	10	4	0.245	439.5	141.0 ± 149.1	50
2	Light	$AgNO_3$	EG	30	4	0.556	434.0	80.1 ± 41.7	85
3	Light	$AgNO_3$	EG	45	4	0.648	425.0	60.5 ± 22.0	51
4	Light	$AgNO_3$	EtOH	30	4	0.271	454.0	68.2 ± 62.3	178
5**	Light	$AgClO_4$	EG	30	4	0.379	420.5	56.8 ± 49.6	203
6	Light	$AgClO_4$	EG	30	4	0.702	417.5	30.9 ± 21.7	416
7	Dark	$AgClO_4$	EG	5	2	0.798	421.5	45.3 ± 12.0	225
8	Dark	$AgClO_4$	EG	5	4	0.668	439.0	49.2 ± 16.0	237
9	Dark	$AgClO_4$	EG	5	8	0.590	439.5	47.0 ± 21.0	290
10	Dark	$AgClO_4$	EG	5	16	0.530	442.0	58.3 ± 19.3	664
11	Dark	AgAc	EG	15	4	0.614	389.5	40.0 ± 17.6	494
12	Dark	$AgNO_3$	EG	5	2	0.383	415.0	62.3 ± 24.0	470
13	Dark	$AgNO_3$	EG	30	4	1.000	439.0	53.5 ± 18.0	211
14	Dark	Ag_2SO_4	EG	5	2	0.656	425.0	66.8 ± 48.9	495
15	Dark	AgAc	EtOH	5	2	0.884	433.0	51.7 ± 16.4	364
16	Dark	$AgClO_4$	EtOH	5	8	0.054	435.5	39.6 ± 16.4	109
17	Dark	$AgNO_3$	EtOH	30	8	0.052	439.0	74.6 ± 26.5	382
18	Dark	$AgClO_4$	Gly	5	2	0.159	456.0	161.6 ± 58.7	198
19	Dark	$AgClO_4$	Gy	5	16	0.306	447.0	70.6 ± 23.2	240

* Normalized to the maximum of the dataset, ** 10 days old sample when STEM grid was prepared

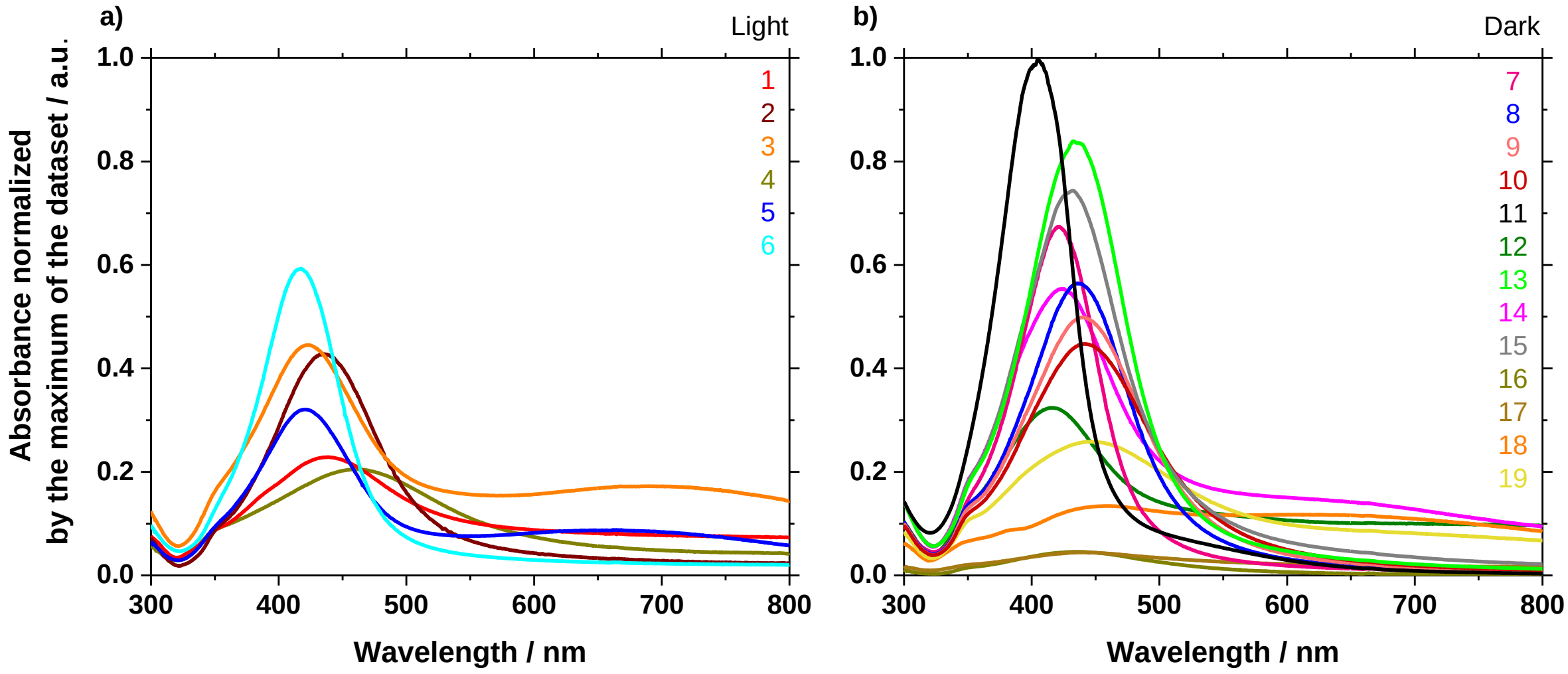

Figure S2. UV-vis spectra for samples corresponding to entries in **Table S3**, as indicated.

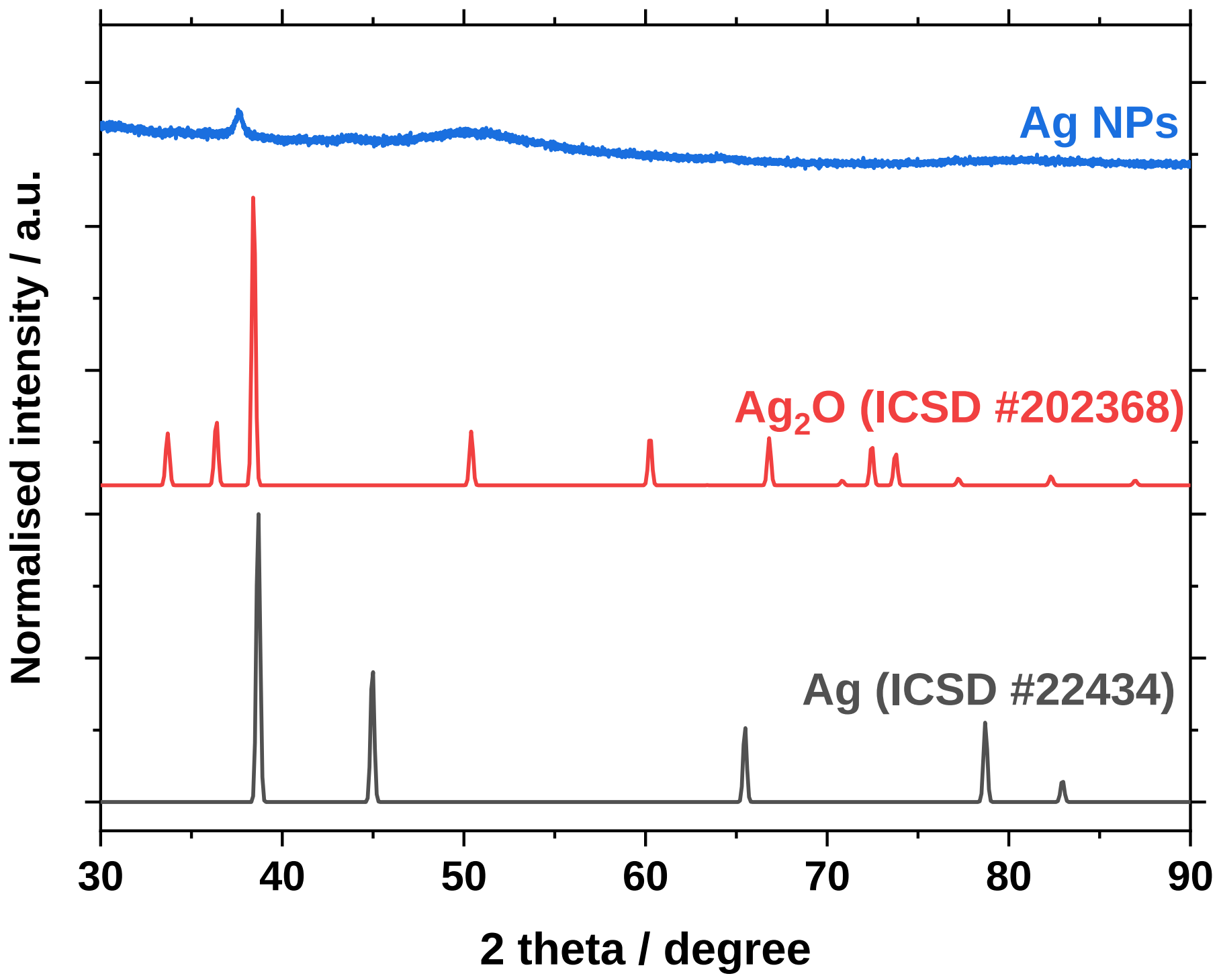

Figure S3. Illustrative XRD diffractogram of the Ag-based materials obtained and references for Ag and Ag_2O.

The resulting diffraction features in **Figure S3** are weak in intensity and only the main diffraction peak of the material is easily observed. The sample exhibit limited peak resolution due to low metal content and nanoscale dimensions of the particles. The data are nevertheless included for completeness and transparency.

The XRD diffractogram of the material obtained indicate the formation of Ag-based NPs. The formation of Ag NPs is supported by the relatively well-pronounced spr peak whereas Ag_2O materials show a broad absorption without a well-defined peak.[50, 51]

1)

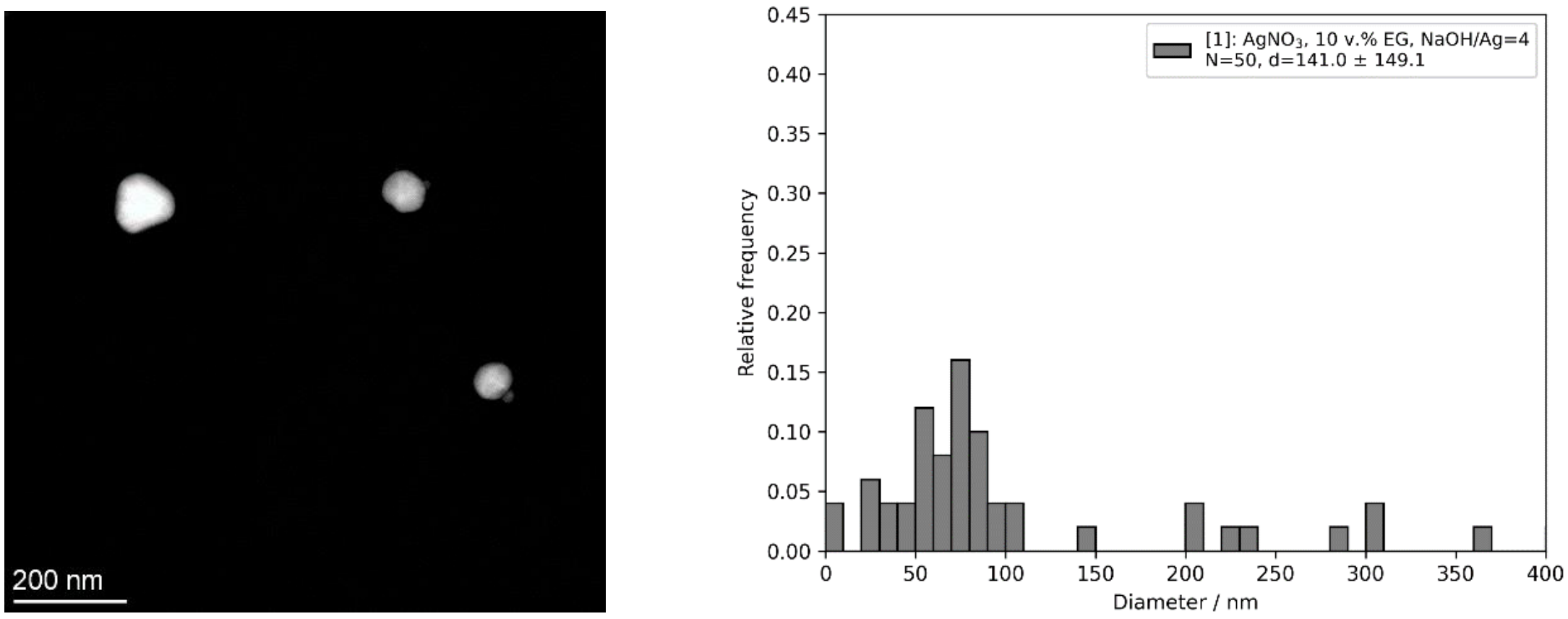
200 nm
[1]: $AgNO_3$, 10 v.% EG, NaOH/Ag=4
N=50, d=141.0 ± 149.1
Relative frequency
Diameter / nm

2)

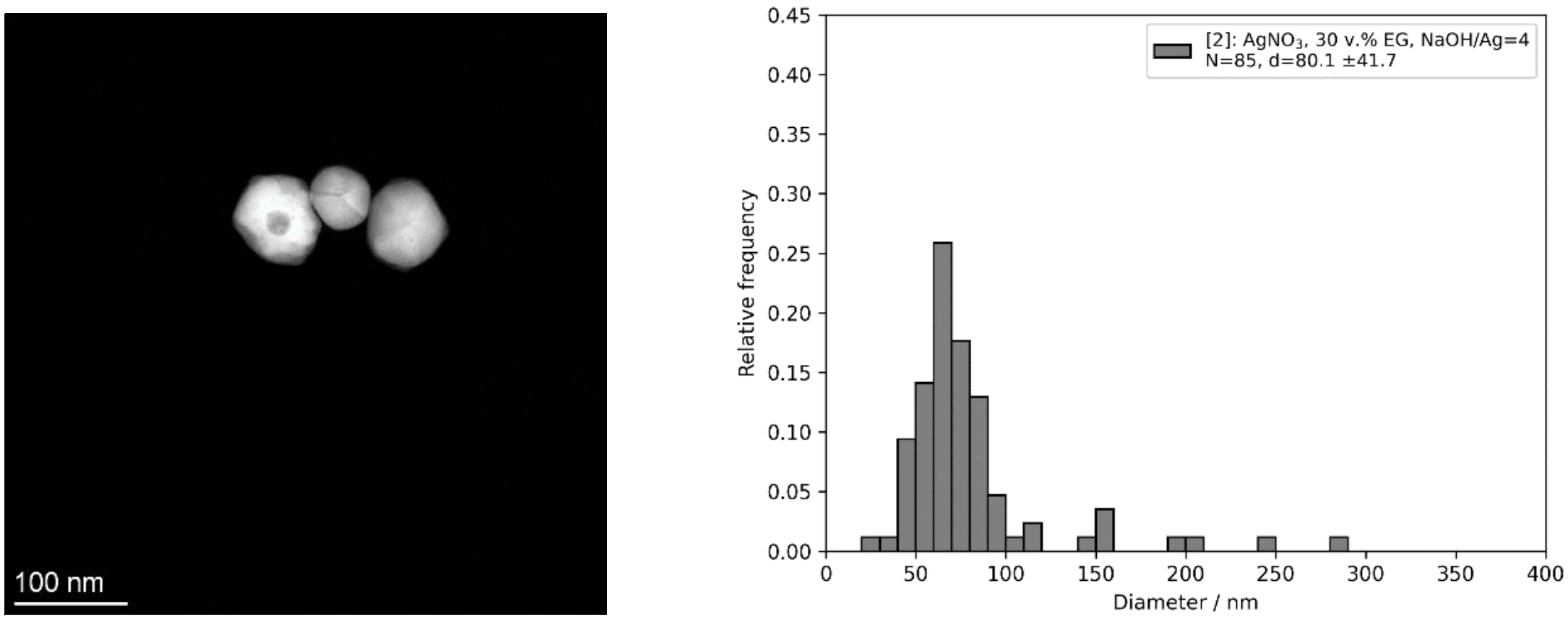
100 nm
[2]: $AgNO_3$, 30 v.% EG, NaOH/Ag=4
N=85, d=80.1 ±41.7
Relative frequency
Diameter / nm

3)

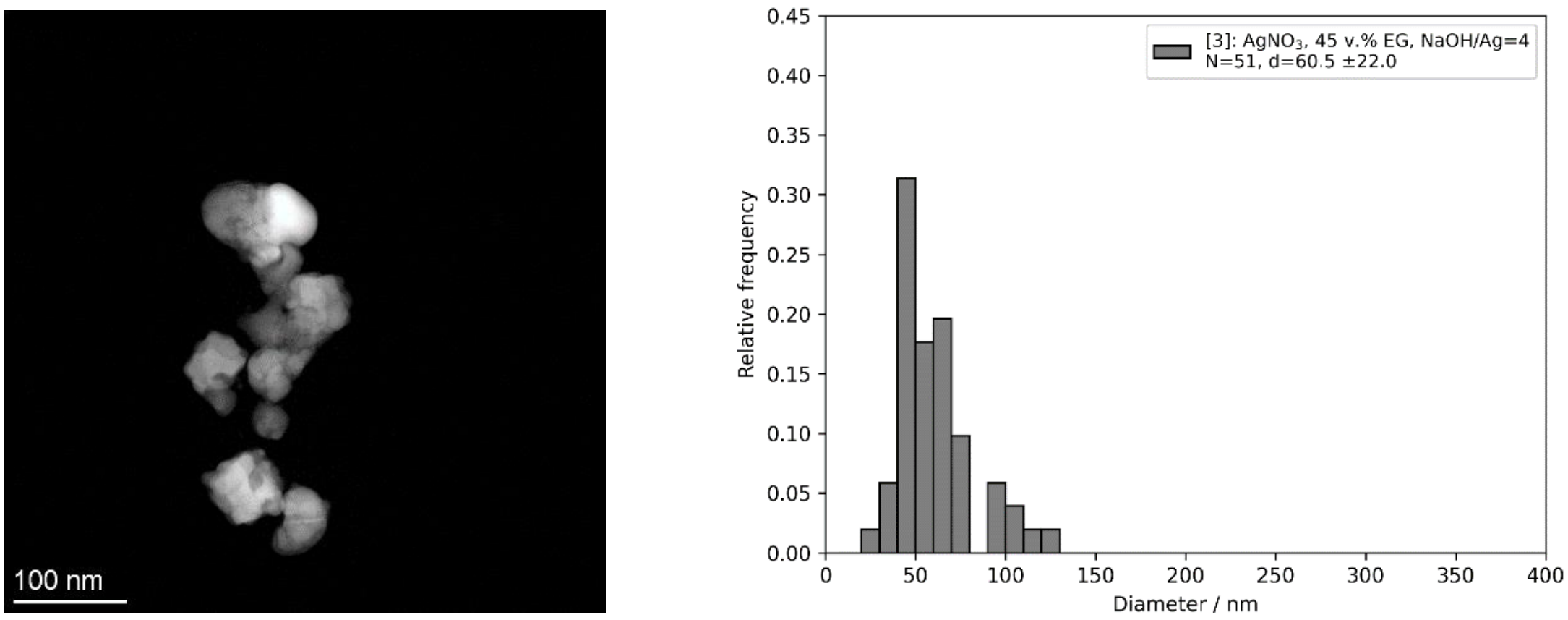
100 nm
[3]: $AgNO_3$, 45 v.% EG, NaOH/Ag=4
N=51, d=60.5 ±22.0
Relative frequency
Diameter / nm

4)

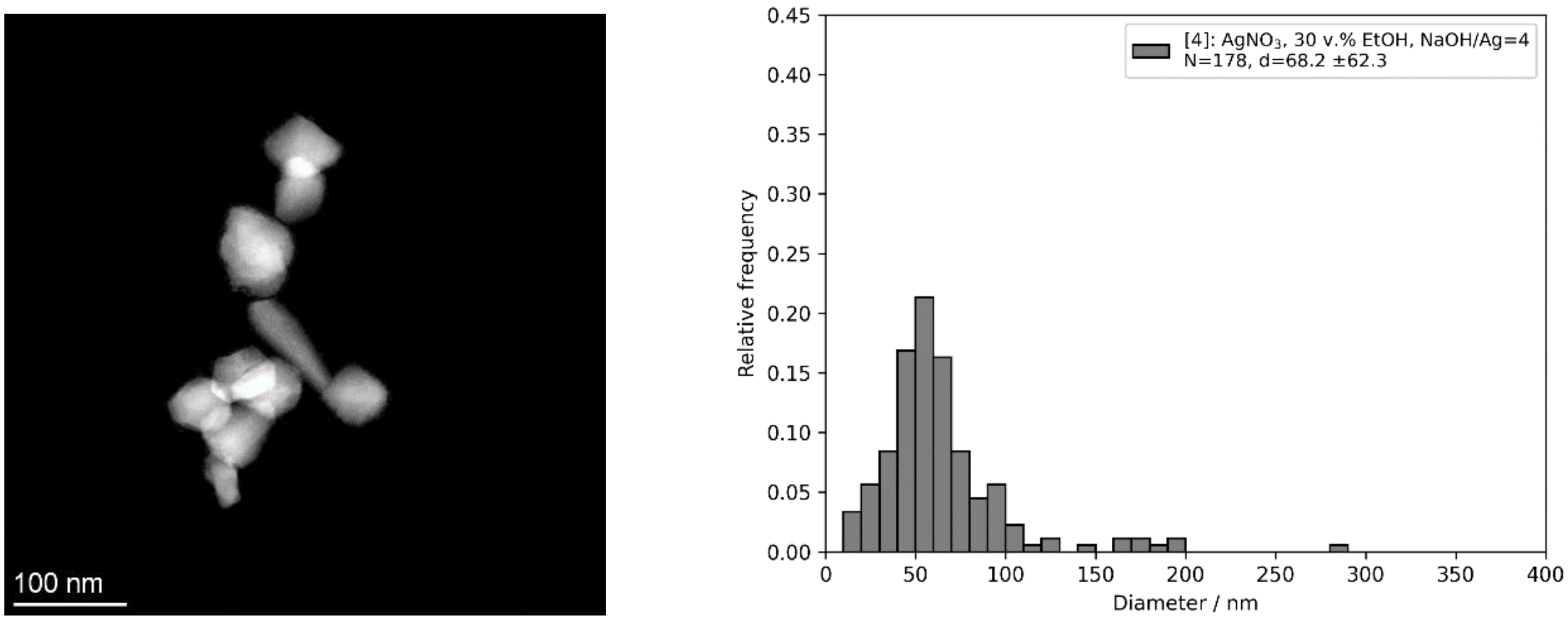
100 nm
[4]: $AgNO_3$, 30 v.% EtOH, NaOH/Ag=4
N=178, d=68.2 ±62.3
Relative frequency
Diameter / nm

5)

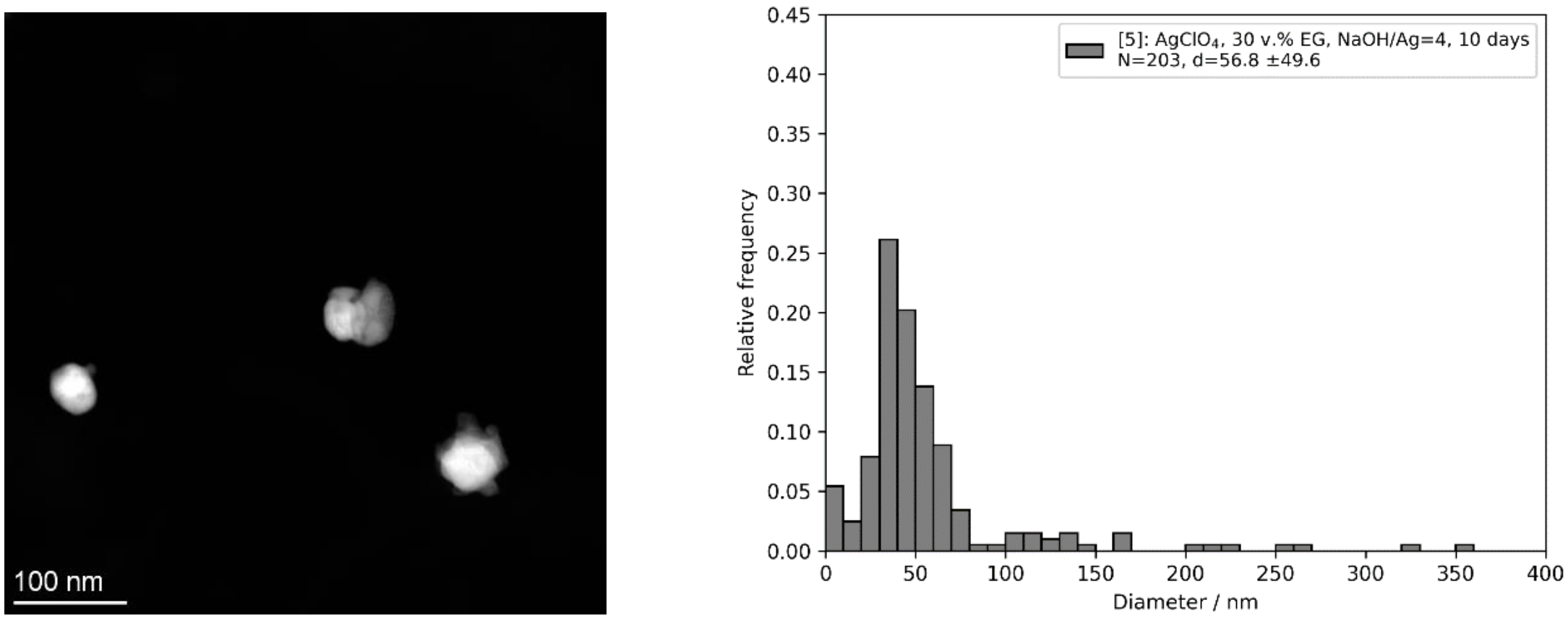
100 nm
[5]: $AgClO_4$, 30 v.% EG, NaOH/Ag=4, 10 days
N=203, d=56.8 ±49.6
Relative frequency
Diameter / nm

6)

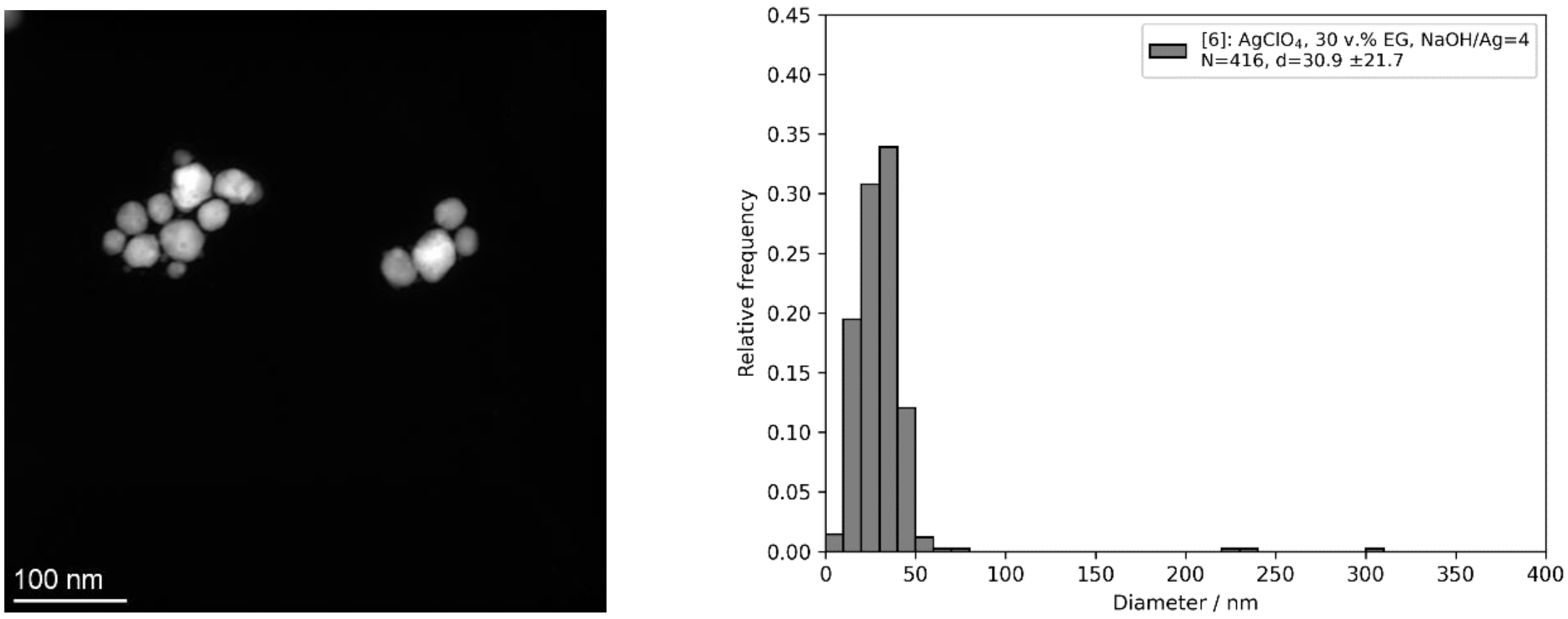
100 nm
[6]: $AgClO_4$, 30 v.% EG, NaOH/Ag=4
N=416, d=30.9 ±21.7
Relative frequency
Diameter / nm

7)

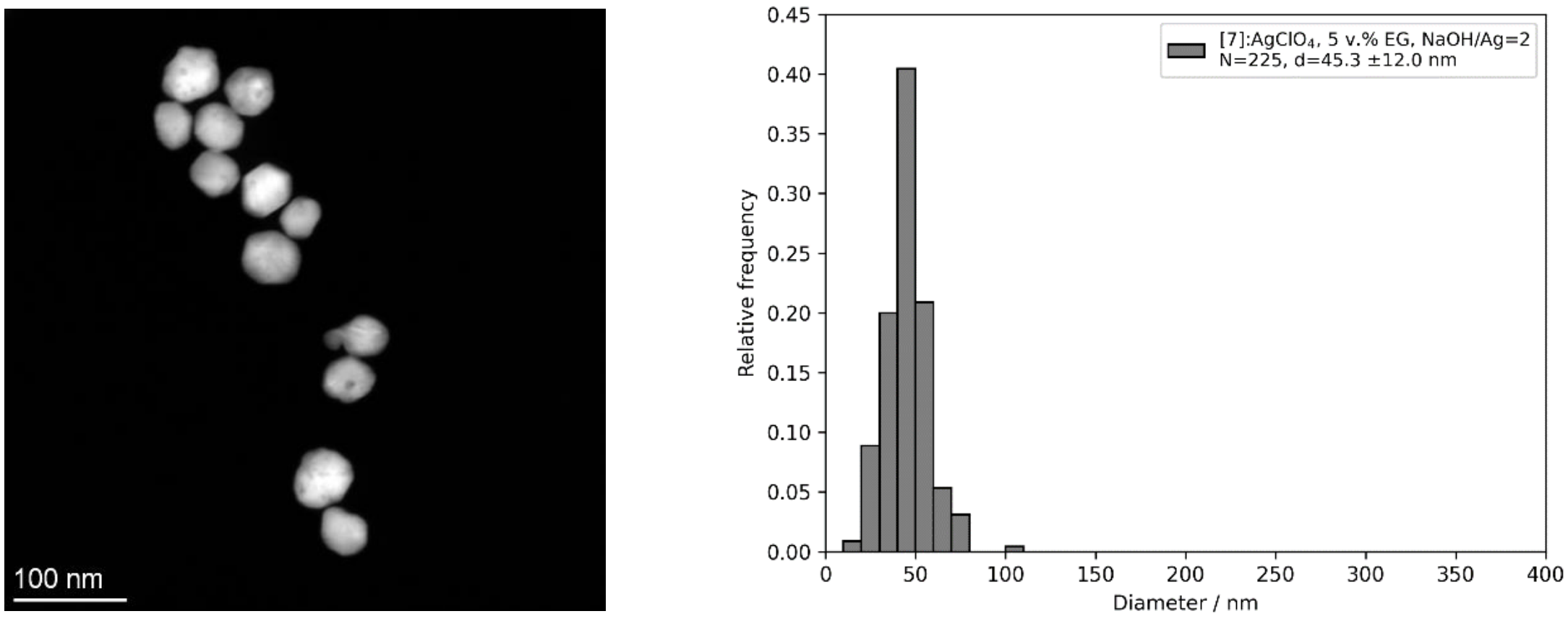
100 nm
[7]:$AgClO_4$, 5 v.% EG, NaOH/Ag=2
N=225, d=45.3 ±12.0 nm
Relative frequency
Diameter / nm

8)

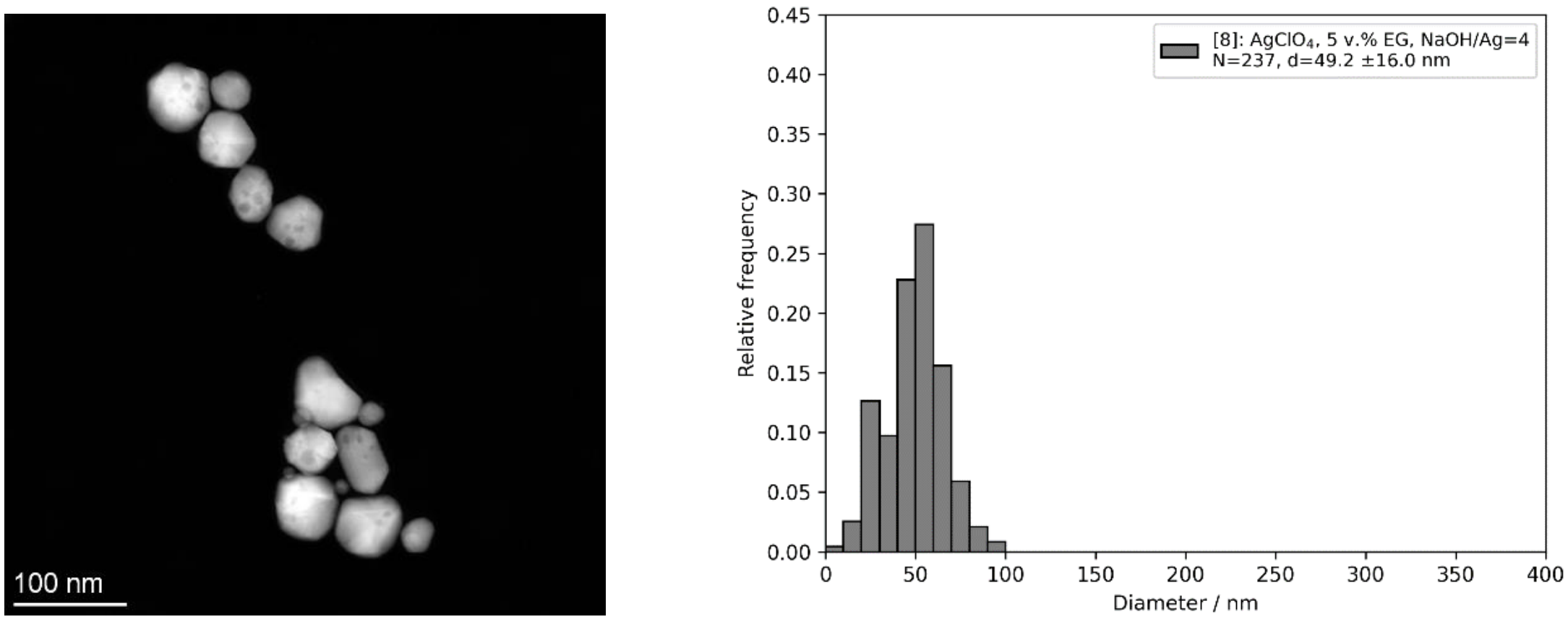
100 nm
[8]: $AgClO_4$, 5 v.% EG, NaOH/Ag=4
N=237, d=49.2 ±16.0 nm
Relative frequency
Diameter / nm

9)

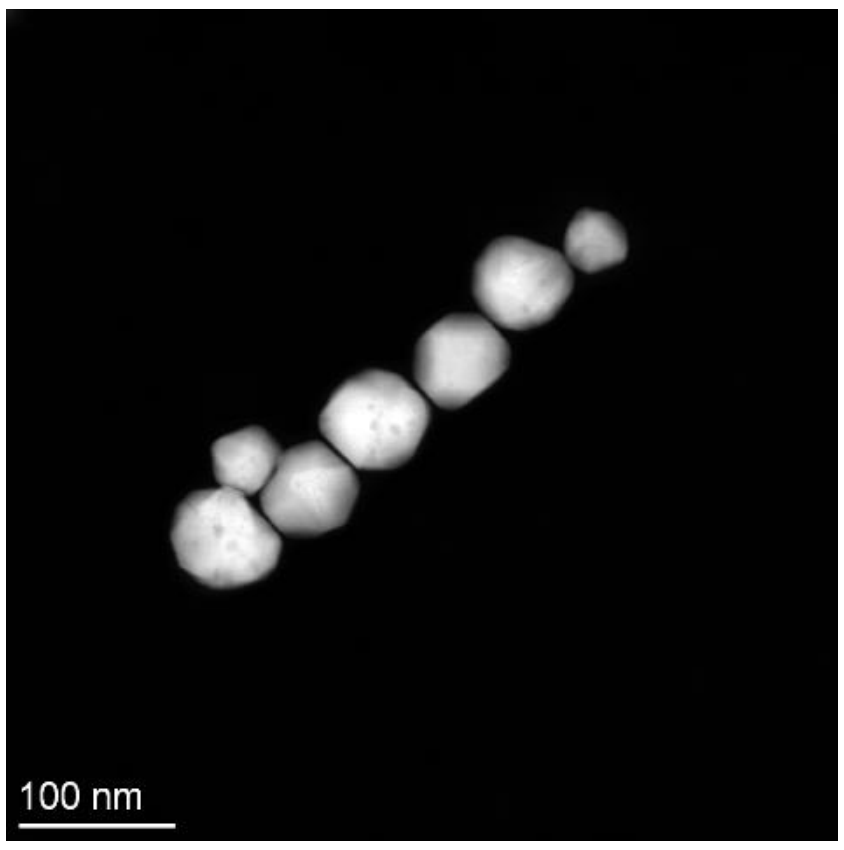
100 nm

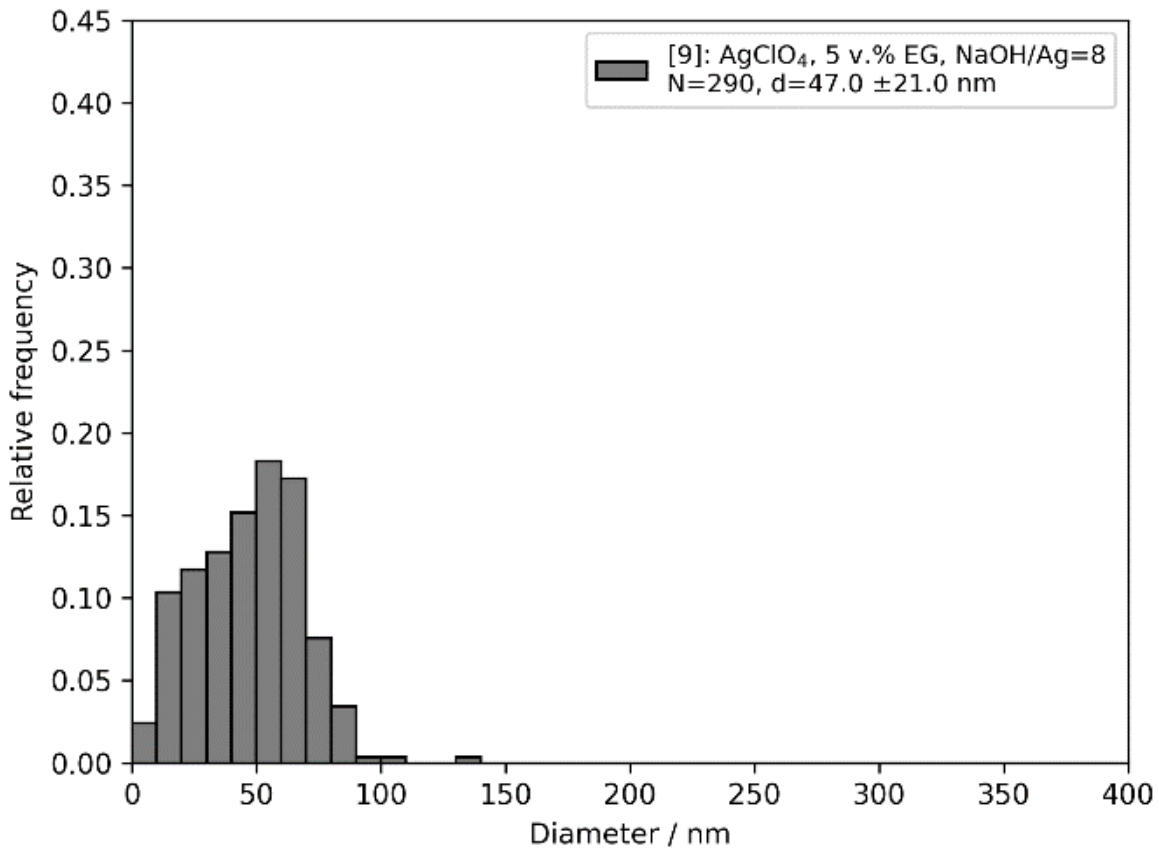
[9]: $AgClO_4$, 5 v.% EG, NaOH/Ag=8
N=290, d=47.0 ±21.0 nm
Relative frequency
Diameter / nm

10)

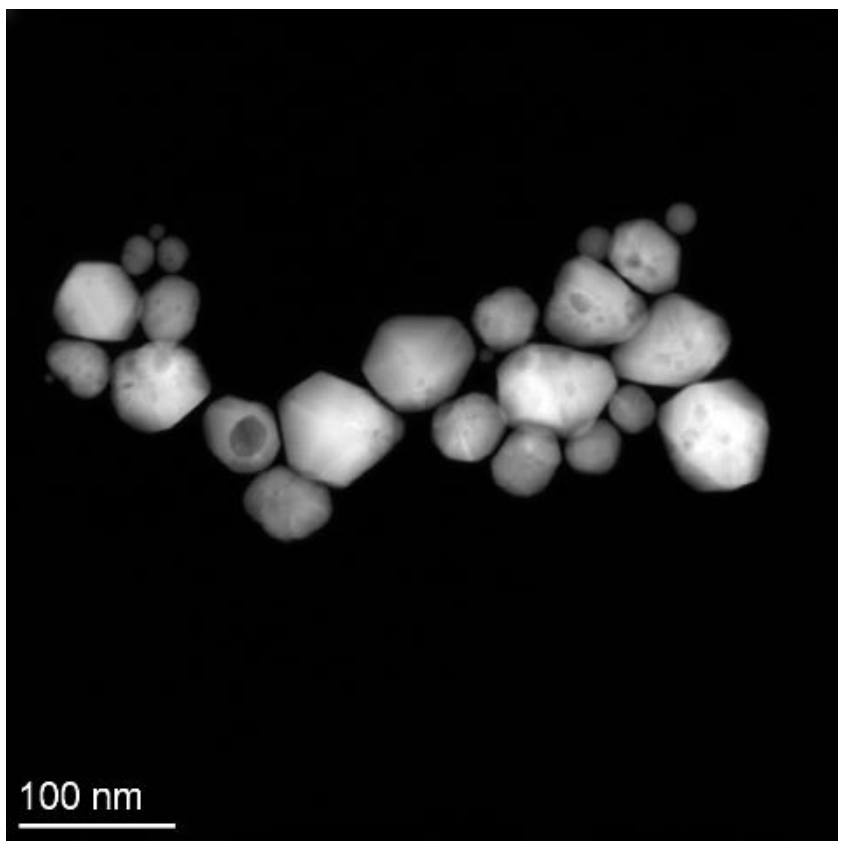
100 nm

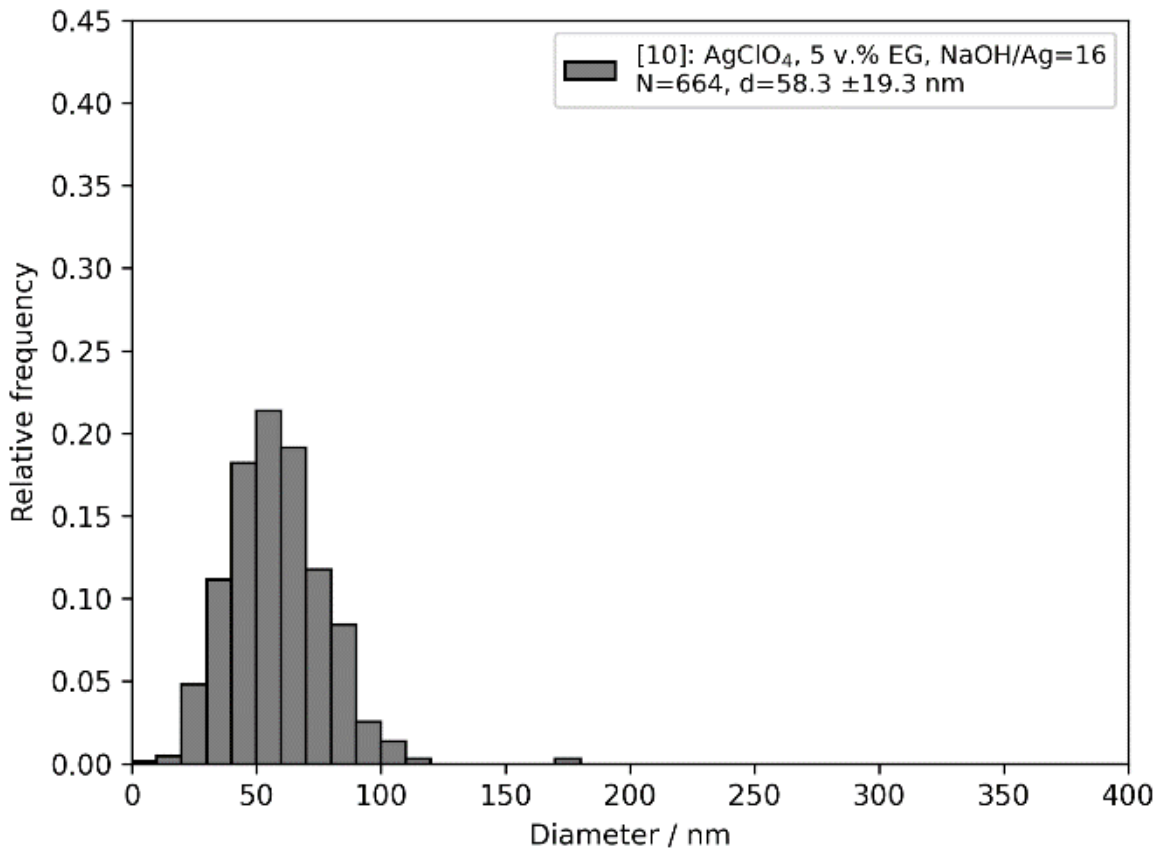
[10]: $AgClO_4$, 5 v.% EG, NaOH/Ag=16
N=664, d=58.3 ±19.3 nm
Relative frequency
Diameter / nm

11)

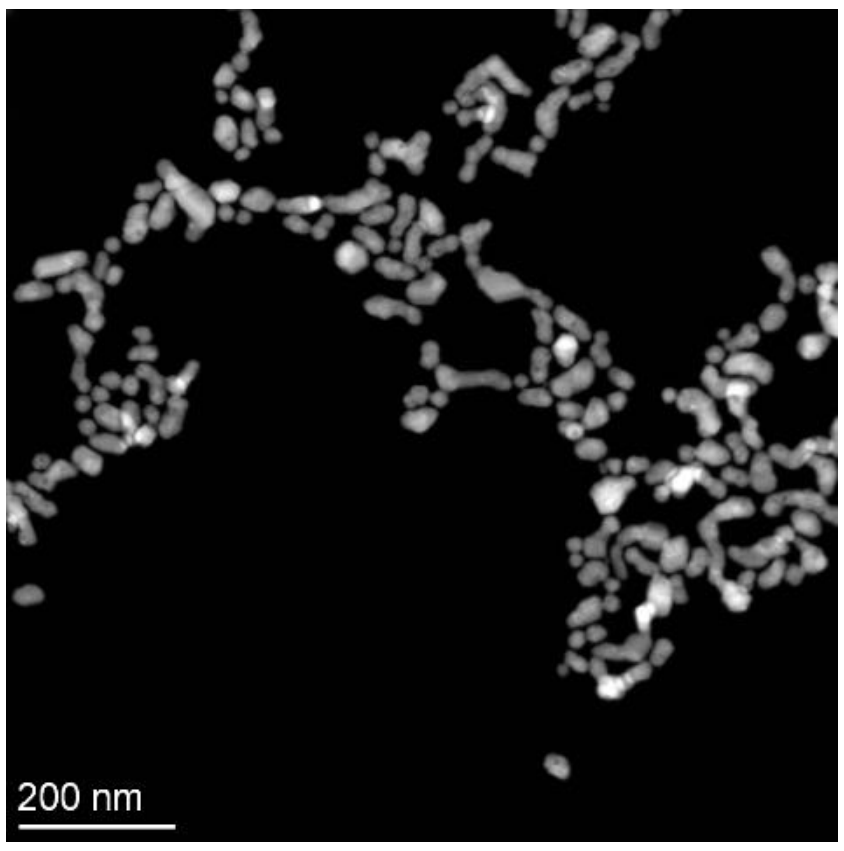
200 nm

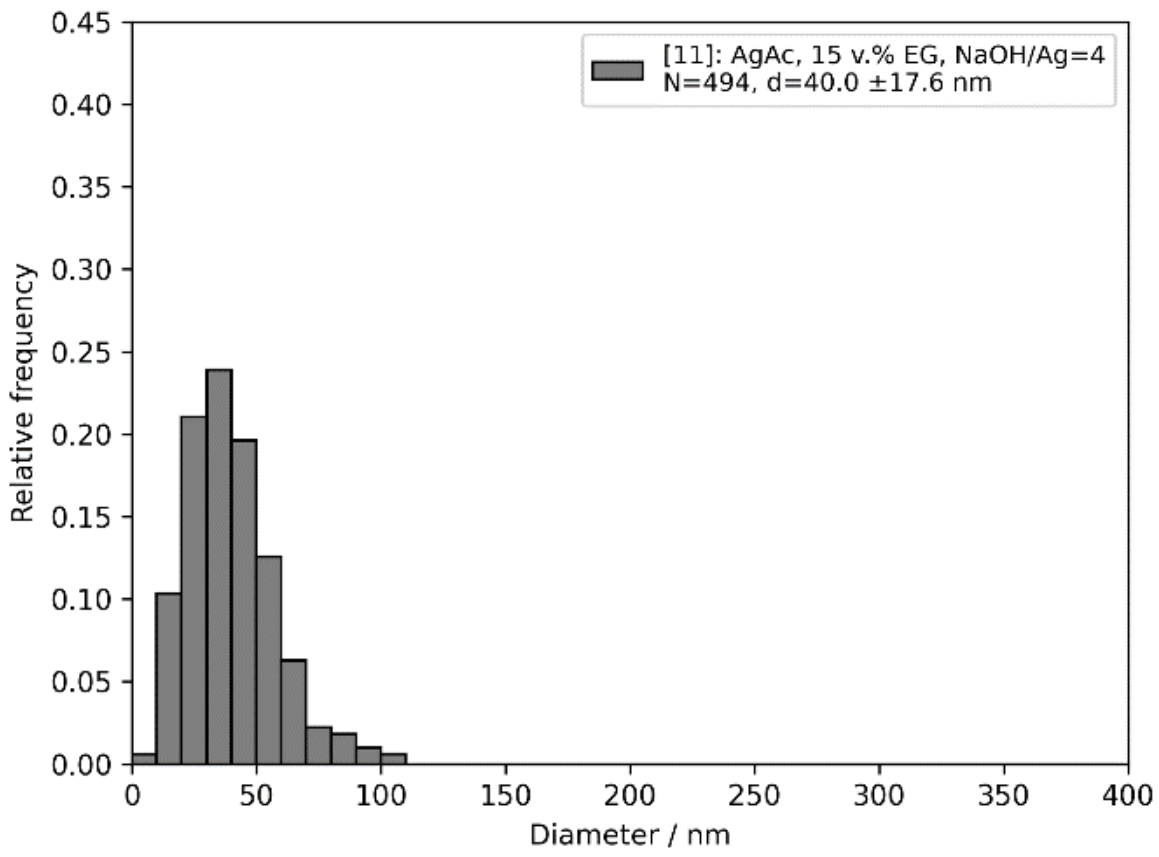
[11]: AgAc, 15 v.% EG, NaOH/Ag=4
N=494, d=40.0 ±17.6 nm
Relative frequency
Diameter / nm

12)

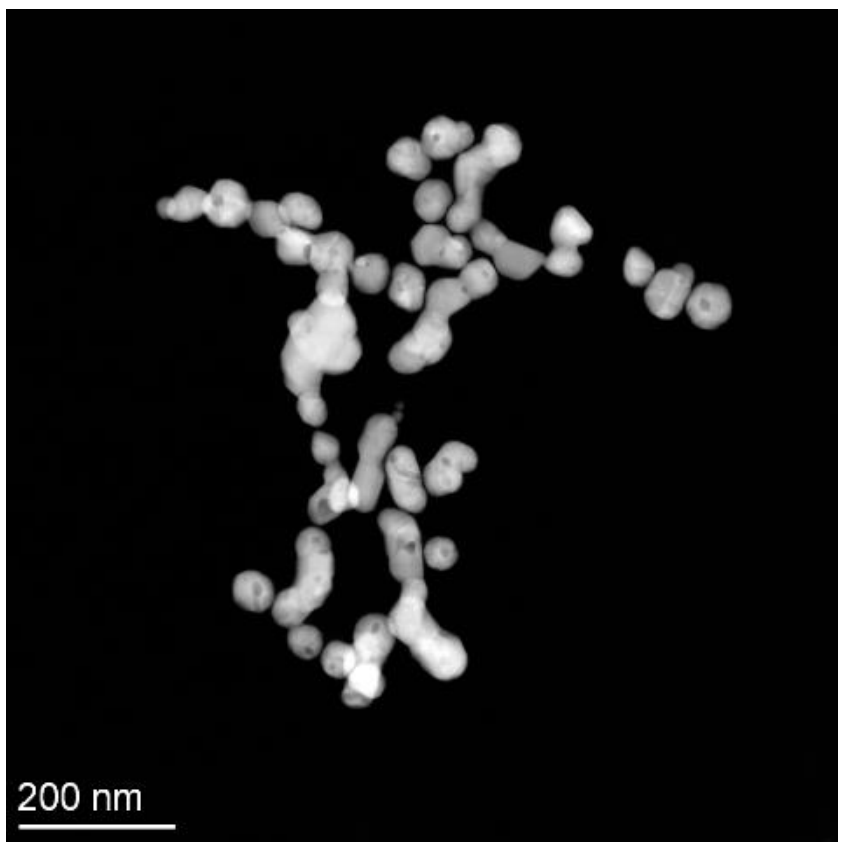
200 nm

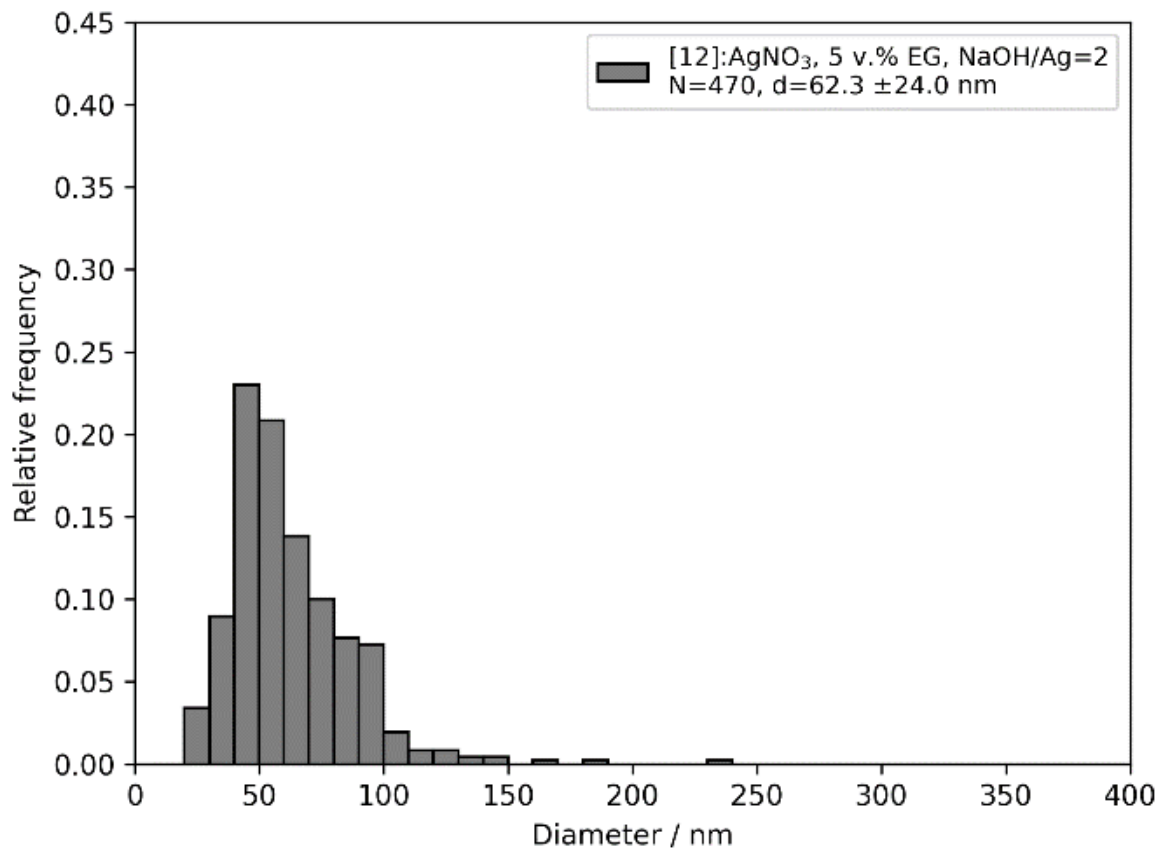
[12]:$AgNO_3$, 5 v.% EG, NaOH/Ag=2
N=470, d=62.3 ±24.0 nm
Relative frequency
Diameter / nm

13)

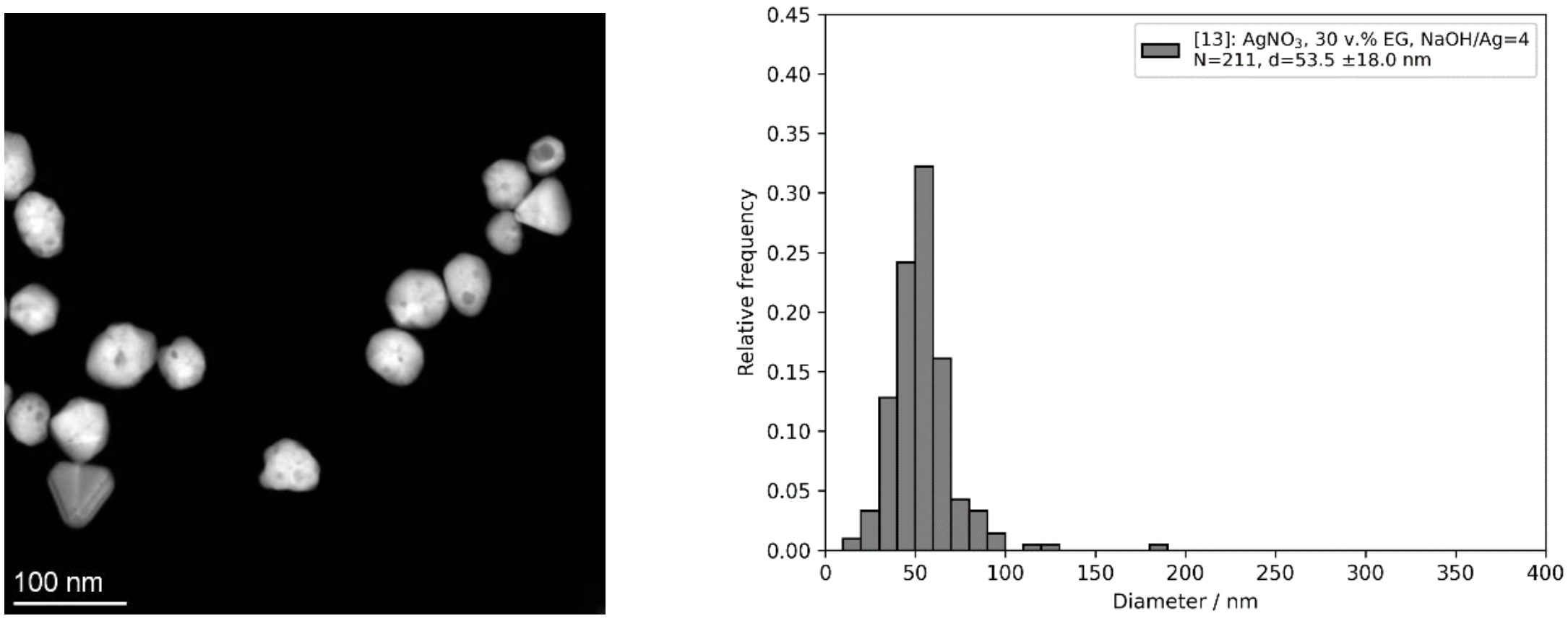
100 nm
[13]: AgNO$_3$, 30 v.% EG, NaOH/Ag=4
N=211, d=53.5 ±18.0 nm
Relative frequency
Diameter / nm

14)

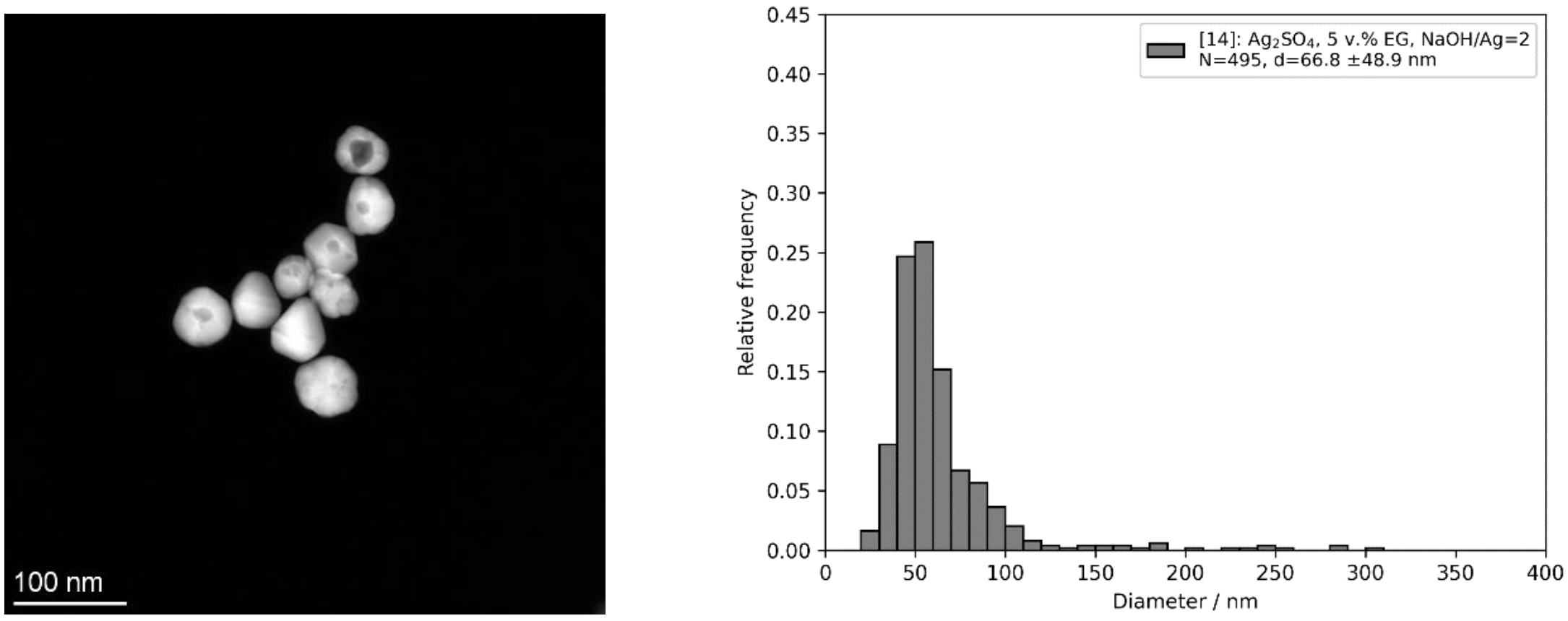
100 nm
[14]: Ag$_2$SO$_4$, 5 v.% EG, NaOH/Ag=2
N=495, d=66.8 ±48.9 nm
Relative frequency
Diameter / nm

15)

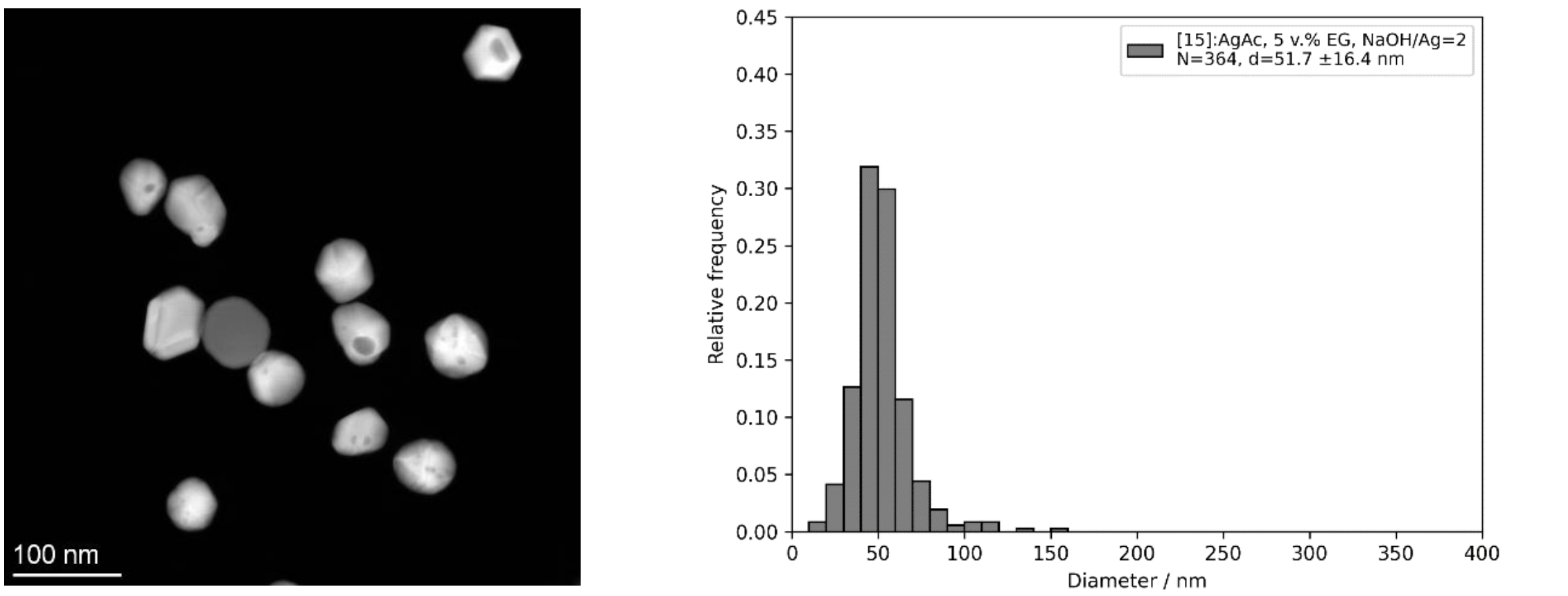
100 nm
[15]:AgAc, 5 v.% EG, NaOH/Ag=2
N=364, d=51.7 ±16.4 nm
Relative frequency
Diameter / nm

16)

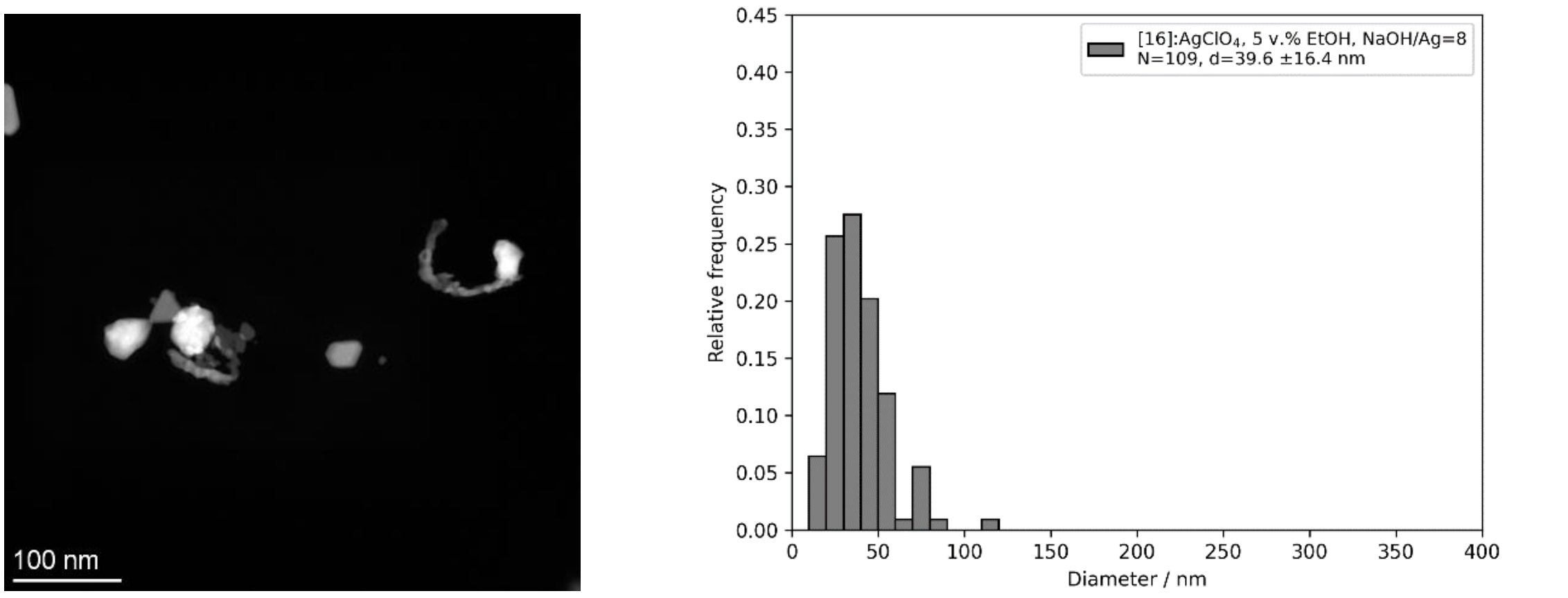
100 nm
[16]:AgClO$_4$, 5 v.% EtOH, NaOH/Ag=8
N=109, d=39.6 ±16.4 nm
Relative frequency
Diameter / nm

17)

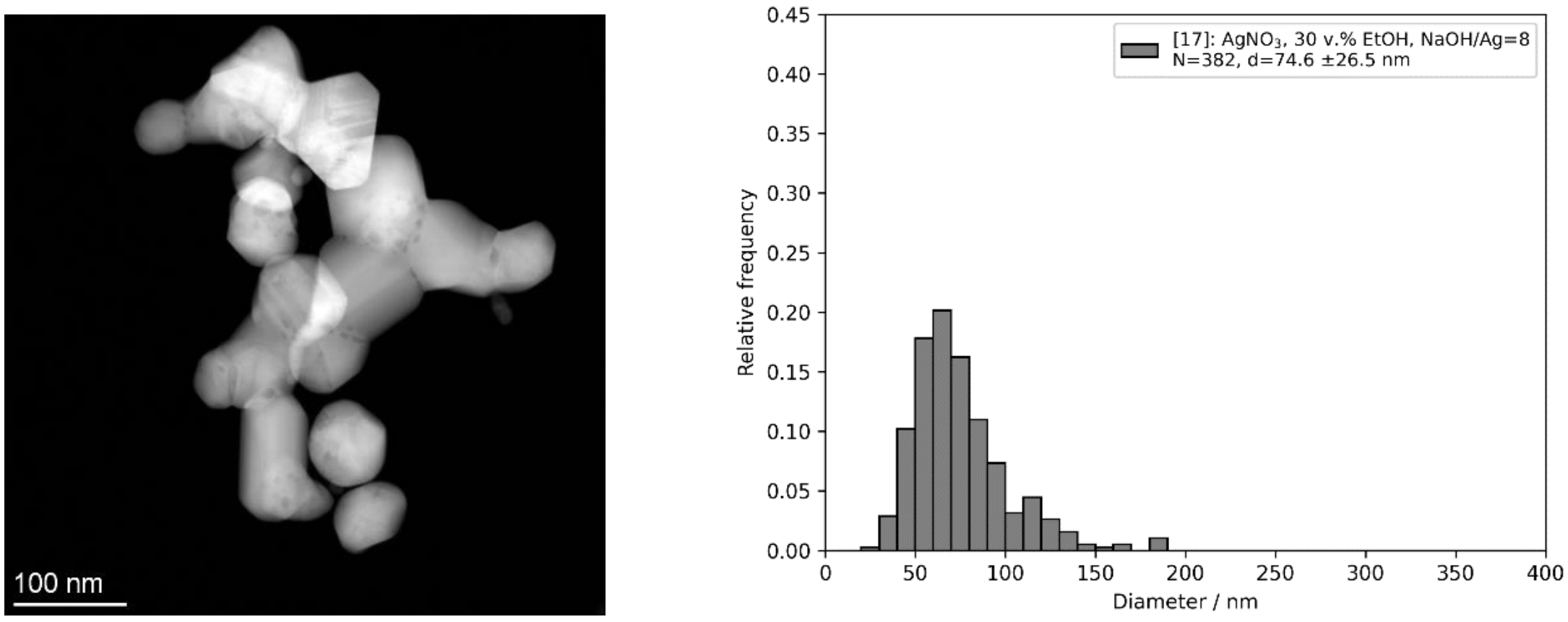

18)

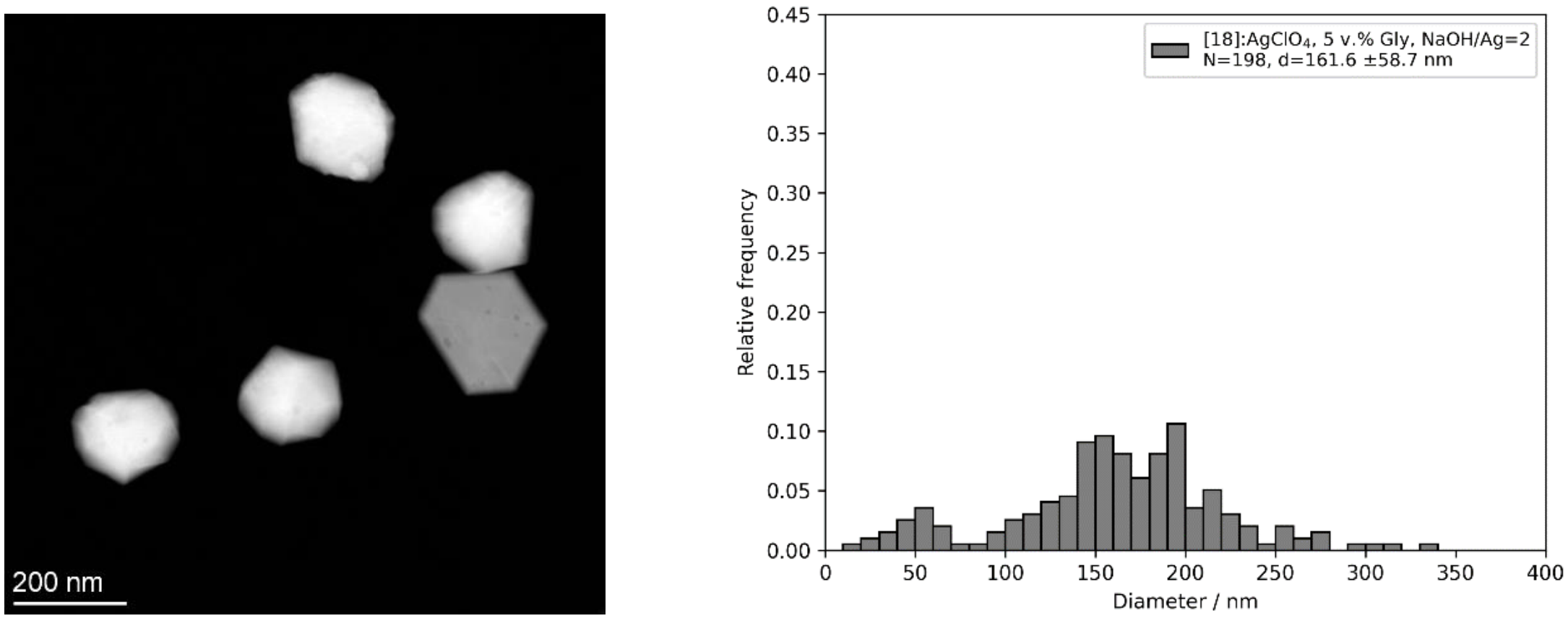

19)

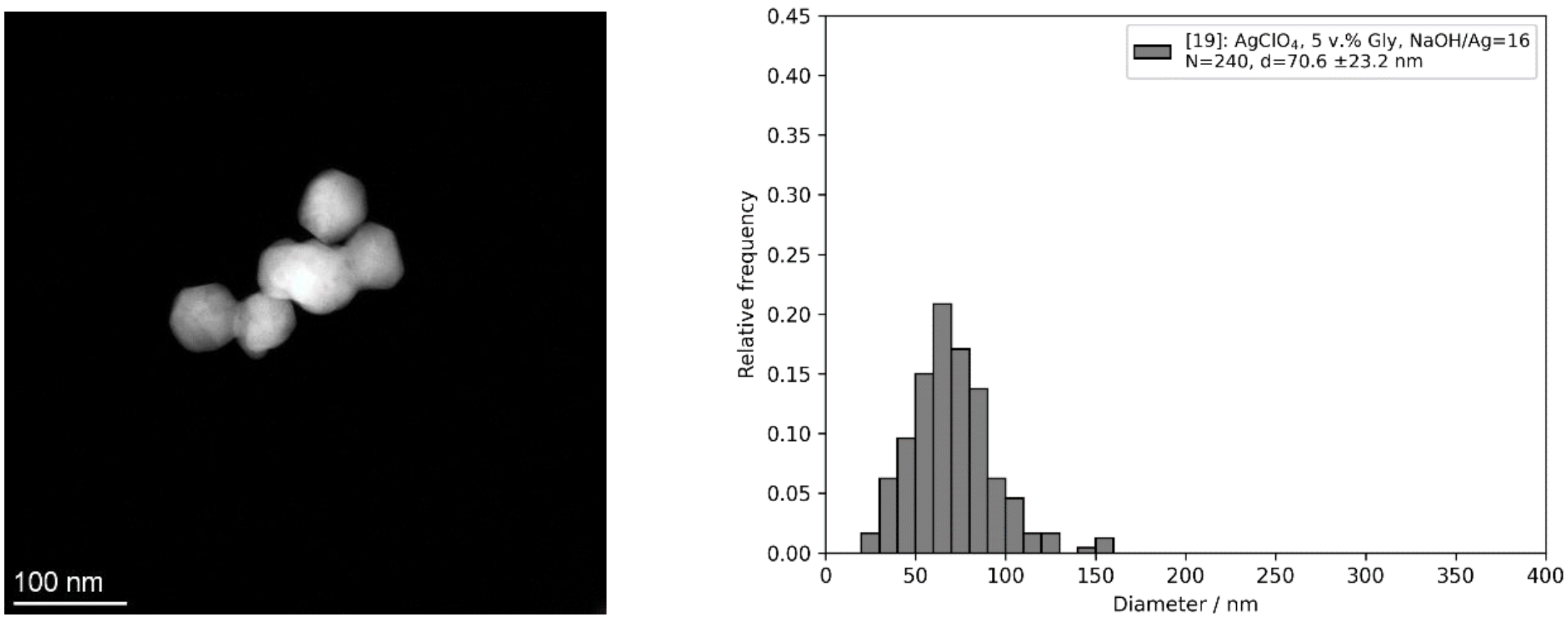

Figure S4. STEM micrographs (left-hand column) and size distribution (right-hand column) for samples corresponding to entries in **Table S3**, as indicated.

Overall, based on UV-vis and STEM data, it can be seen that EtOH and glycerol do not lead to very well defined and small NPs. In the case of AgAc at relatively low EG content and low NaOH/Ag molar ratio there is a tendency to obtain elongated materials (entry 11 in **Figure S4**).

S3. Influence of light

3.1. Influence of light on the synthesis

In **Figure S5** are reported UV-vis spectra and in **Figure S6** the A_{spr} and λ_{spr} values for samples prepared in different ways and then stored in different conditions. A constant value of A_{spr} and λ_{spr} indicates a stable colloidal dispersion over time.

A first simple approach is to perform the synthesis at ambient or controlled light (here controlled light in a photobox). For RT synthesis of Au NPs, a simple manual shake is enough to obtain small size colloidal NPs.[5, 52] A manual shake did not lead to stable colloids for Ag NPs and stirring is here preferred. Living the reaction medium exposed to ambient or controlled light for 2 or 1 hours did not influence much the properties of the Ag colloids. This result suggests that the reaction occurs in less than an hour as discussed in **section S5. Kinetics**.

In terms of storage, based on the A_{spr} values, storage in a fridge tends to lead to more stable colloidal dispersions overtime, as it can be expected. However, a promising condition leading to even more stable colloids comes when the synthesis is performed in the dark (wrapped in aluminum). When the samples prepared in the dark were then kept at RT (in the dark), they showed long term stability. This finding is important to develop simpler and cheaper syntheses since it suggests that long term storage can be achieved without the need for an (energy consuming) fridge. As detailed in the manuscript, the storage can even be performed at light conditions and the colloids are stable, provided the synthesis itself is performed under dark conditions. The exact reason behind this stability is not established yet.

It is also important to note that lower A_{spr} and higher λ_{spr} values were obtained for synthesis performed in the dark compared to those obtained under light conditions. This indicates that larger NPs are obtained when the synthesis is performed in the dark but also more stable dispersions were obtained, which is considered a significant advantage.

In **Figure S6a**, the increase of the λ_{spr} values for the samples shaken under light conditions (stars and hexagone datapoints) is due to the small signal (see corresponding lower A_{spr} values in **Figure S6b**) obtain that leads to a broad spr feature.

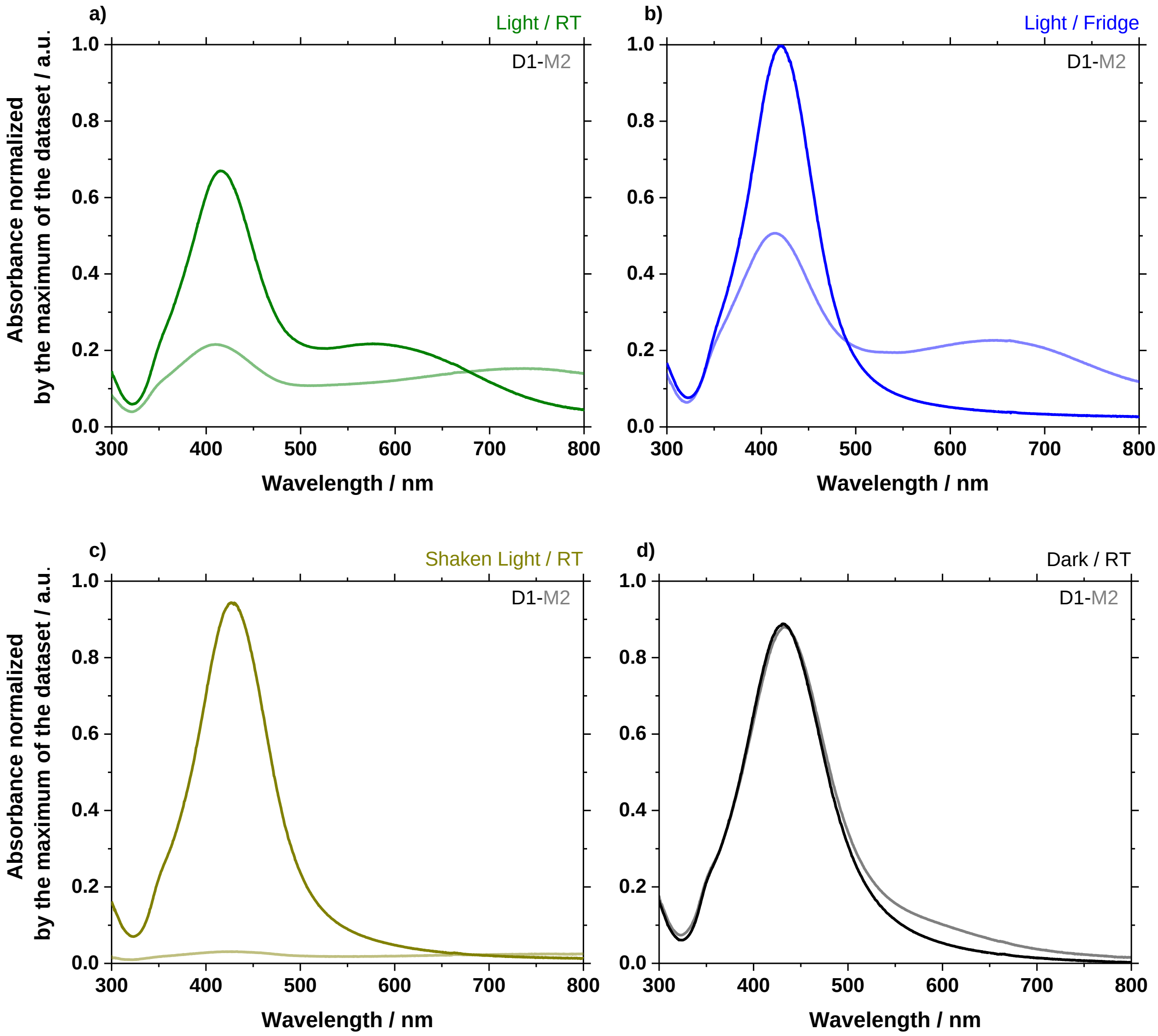

Figure S5. Illustrative UV-vis spectra for samples prepared using 30 v.% EG, 0.25 mM $AgClO_4$ and 1 mM NaOH (NaOH/Ag molar ratio of 4). *Shaken* means that the samples were shaken 10 times (under ambient light conditions). *Dark* means that the sample was wrapped in aluminum foil and stirred for 2h in dark (and kept in aluminum foil in this case to prevent light to interact during the synthesis and storage). *Fridge* means that the sample was stored in a fridge. In darker color are the spectra recorded after 1 day (D1) and in lighter color the spectra recorded after two months (M2).

a)

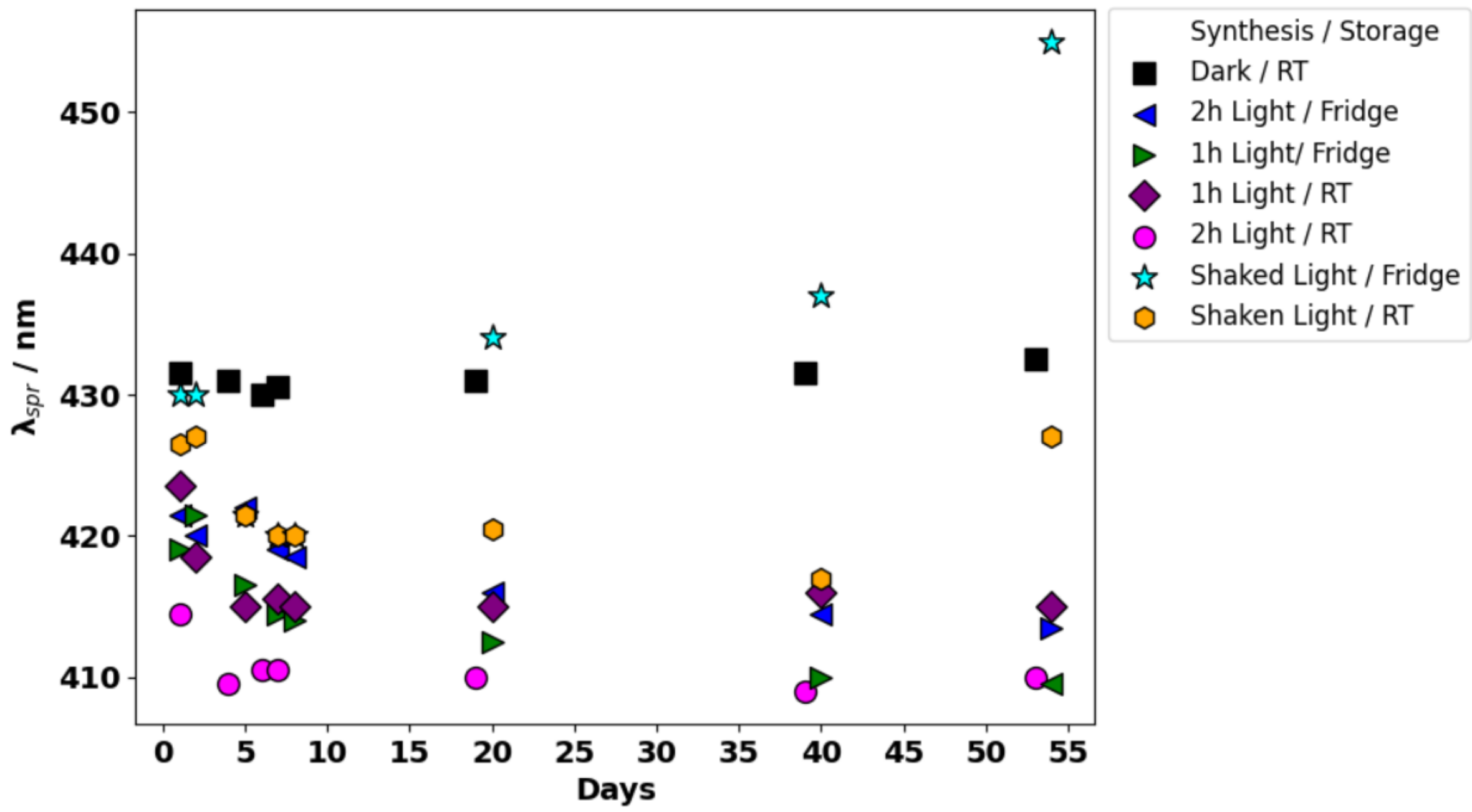

b)

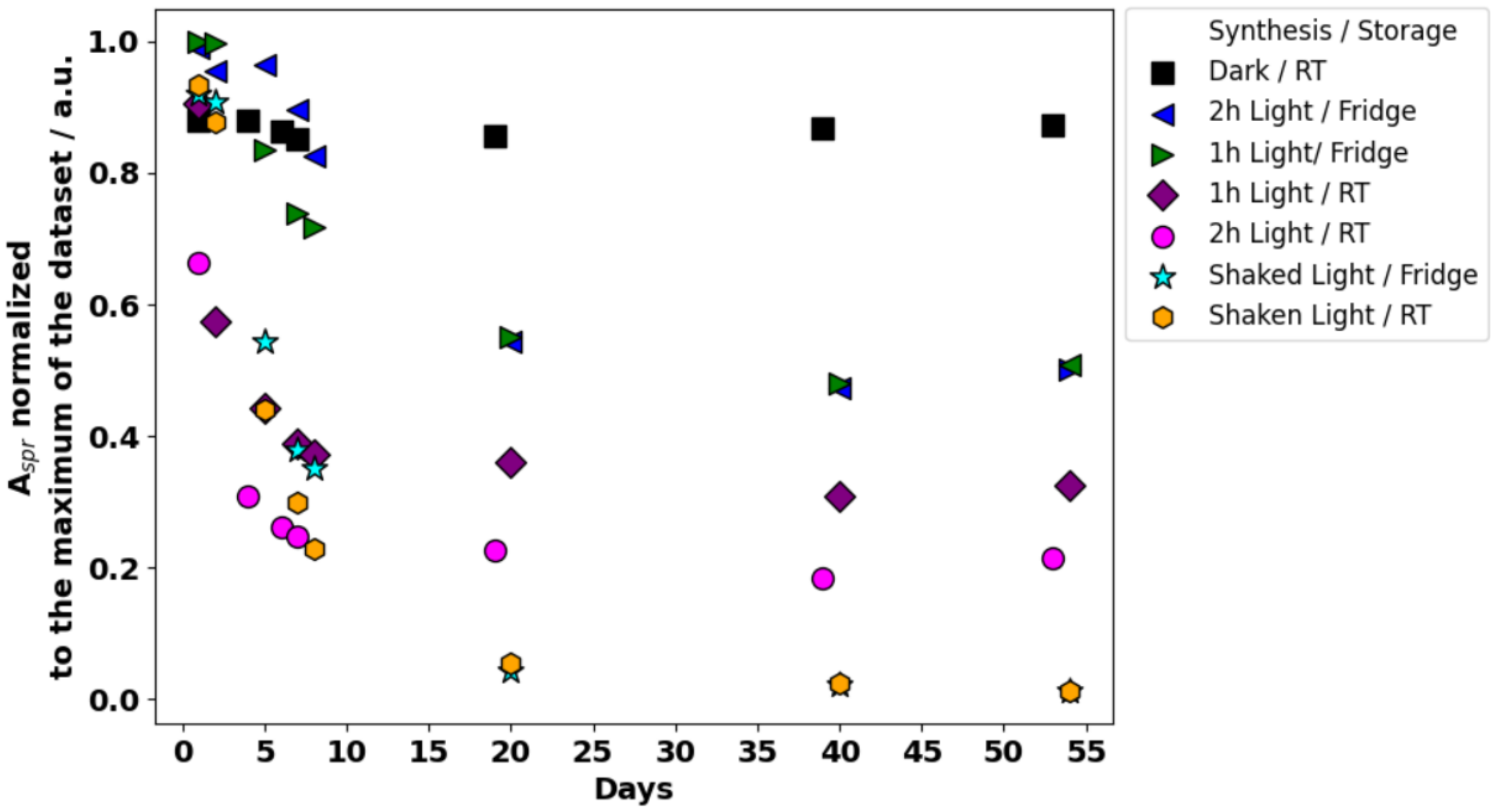

Figure S6. Influence of synthesis and storage conditions over time for samples prepared using 30 v.% EG, 0.25 mM $AgClO_4$ and 1 mM NaOH (NaOH/Ag molar ratio of 4). *Shaken* means that the samples were shaken 10 times (under ambient light conditions). *Dark* means that the sample was wrapped in aluminum foil and stirred for 2h in dark (and kept in aluminum foil in this case to prevent light to interact during the synthesis). *Fridge* means that the sample was stored in a fridge.

3.2. Influence of light on storage

Having established the influence of light on the synthesis and given the higher stability observed when the samples was kept in the dark, a more specific focus is given to the importance of light on storage.

Figure S7 illustrates that in addition to a decrease of the A_{spr} values, samples exposed to light during synthesis tend to show a second maxima at higher wavelengths in UV-vis, indicating size and/or shape modification, also observed on **Figure S5**. This was not investigated further at this stage.

In **Figure S8** are reported the A_{spr} and λ_{spr} values for samples prepared in different ways and then stored in different conditions. This Figure is complementary to **Figure 2** in the main manuscript. The conditions are always 2 hours synthesis and then storage under different conditions: (i) synthesis in the dark and storage in the dark, (ii) synthesis in the dark and storage in a fridge (which is also a dark condition environment), (iii) synthesis in the dark and storage in the light at RT, (iv) a control with synthesis in the light and storage in the dark.

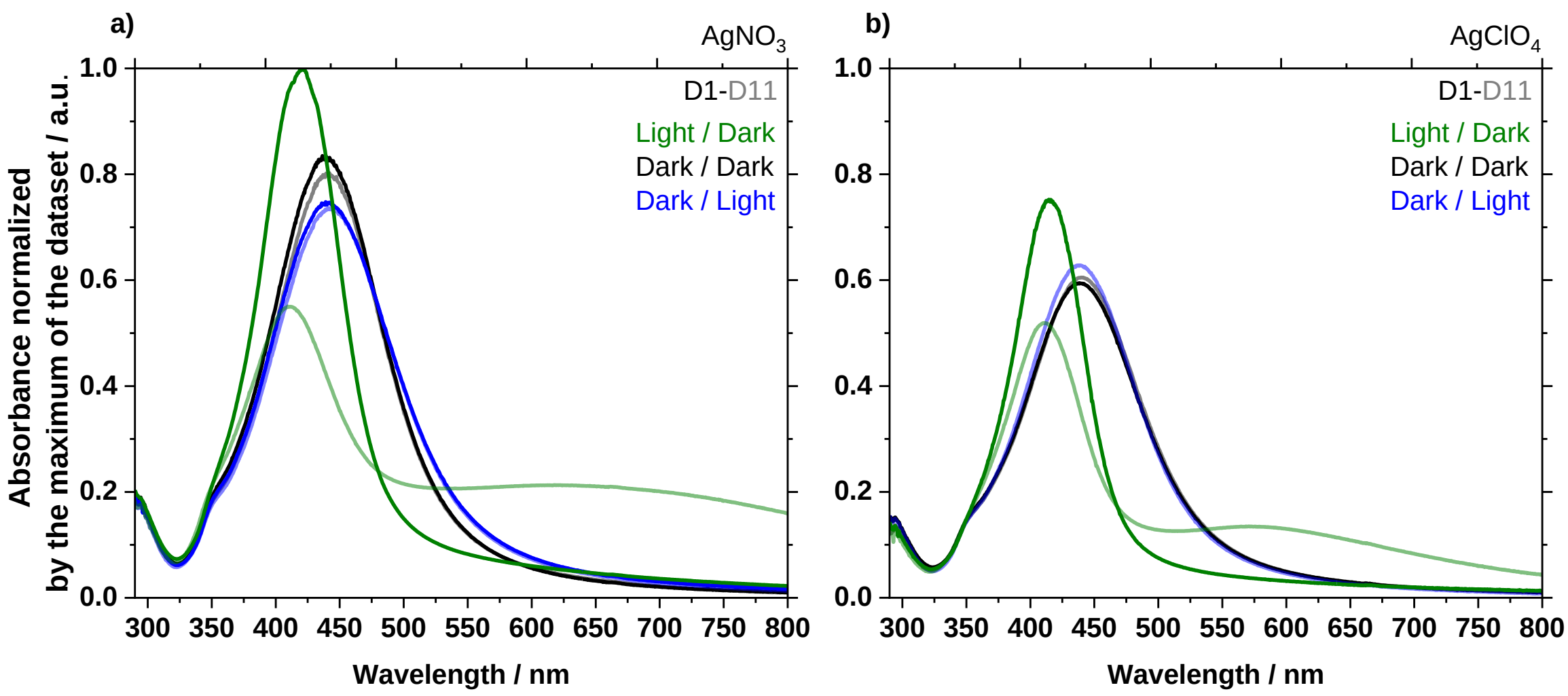

Figure S7. Influence of light exposure during and after synthesis for samples prepared using 30 v.% EG, 0.25 mM (a) $AgNO_3$ or (b) $AgClO_4$, and 1 mM NaOH (NaOH/Ag molar ratio of 4), as indicated. The spectra recorded a day after synthesis (D1) are darker and the spectra recorded eleven days after synthesis (D11) are lighter and only changed for the conditions Light / Dark.

a)

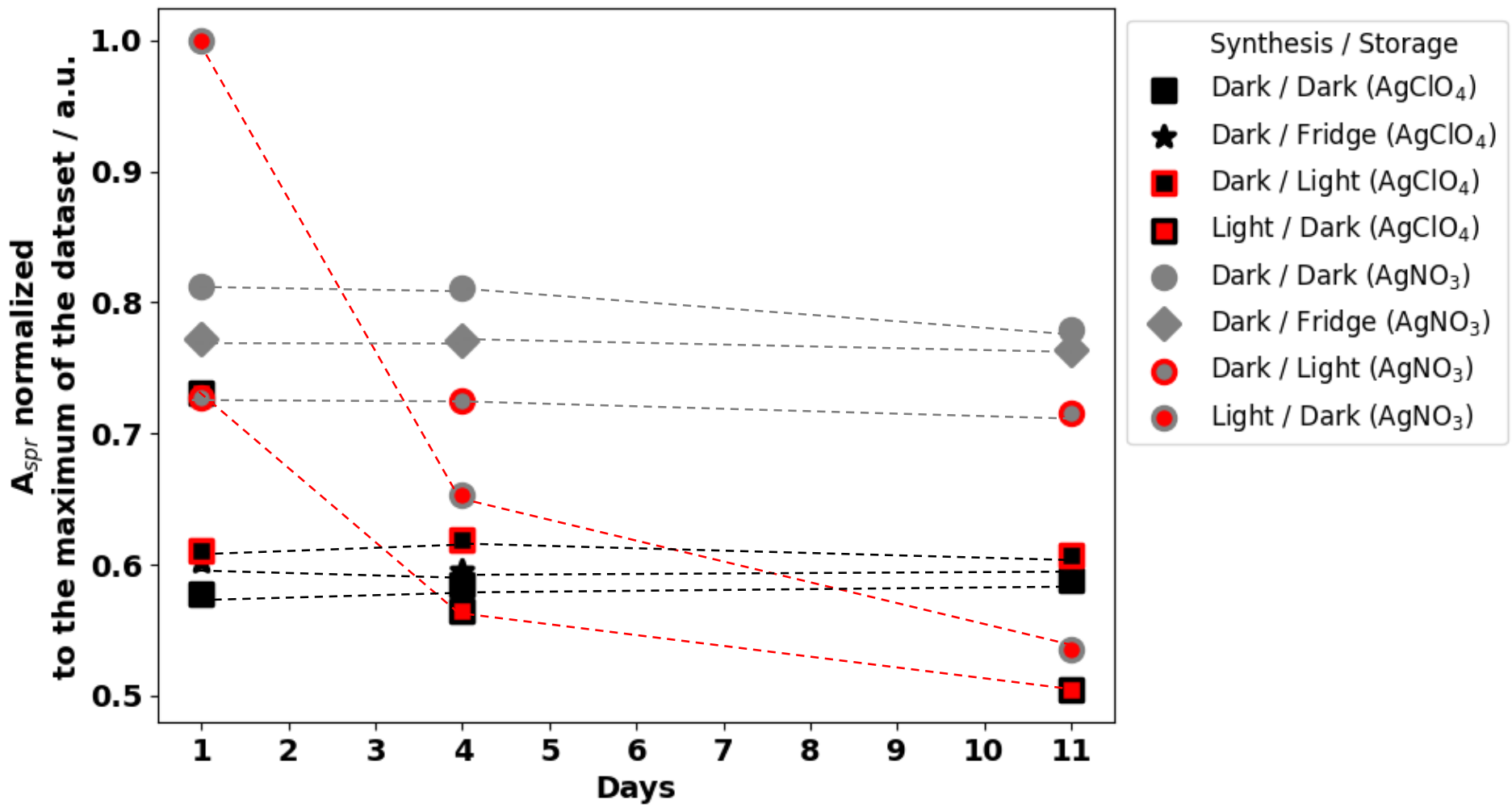

b)

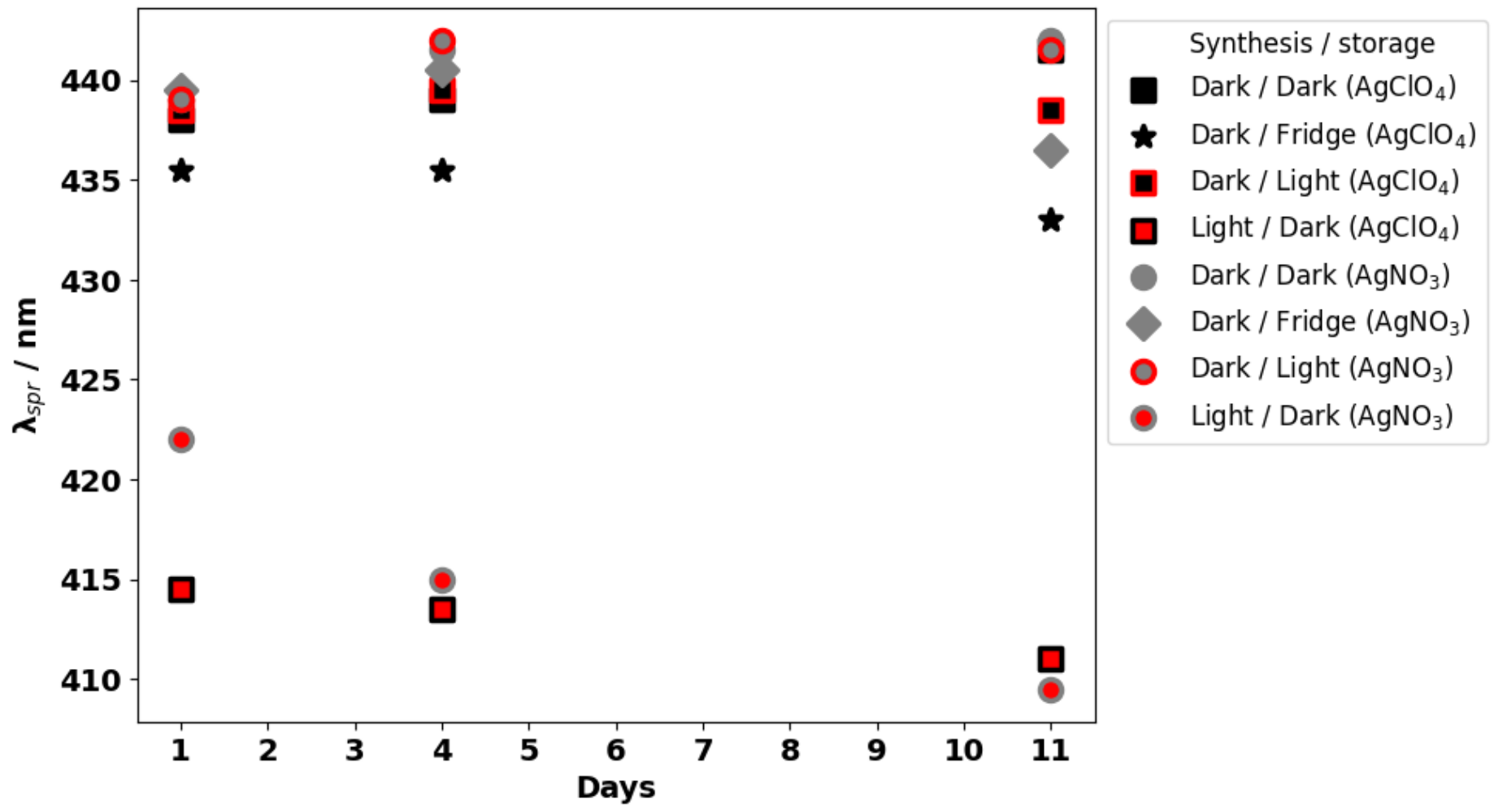

Figure S8. Influence of light exposure during and after synthesis for samples prepared using 30 v.% EG, 0.25 mM $AgNO_3$ or $AgClO_4$, as indicated, and 1 mM NaOH (NaOH/Ag molar ratio of 4) considering (a) A_{spr} or (b) λ_{spr} values. The dotted lines are a guide for the eyes and the red dotted line correspond to conditions where the synthesis was performed at ambient light whereas the gray/dark dotted lines correspond to cases where the synthesis was performed under dark conditions. Results from **Figure 2** in the main manuscript are reproduced in (a) for comparison.

Performing the synthesis is the dark (here for 2 hours) seems to be the key to ensure colloidal stability over extended period of time.

S4. Parametric study

In this section, we try to give an account of the influence of each parameters. We report on the influence of the nature of the alcohol used as source of reducing agent (methanol, EtOH, EG, glycerol), the alcohol content (5-97.5 v.%), the concentration of NaOH required (NaOH/Ag molar ratio from 2 to 16, for a precursor concentration of 0.25 mM) and the metal precursor used ($AgNO_3$, $AgClO_4$, Ag_2SO_4, AgAc), for RT syntheses of Ag NPs performed without the need for other chemicals. With the objective to develop more sustainable syntheses, a focus is given to conditions leading to (i) small size NPs, relevant for instance for catalysis by maximizing surface area, (ii) obtained with relatively low base concentrations (NaOH/Ag molar ratio < 20, for a concentration in Ag of 0.25 mM), (iii) low toxicity alcohols and (iv) low amount of alcohol (< 50 v.%).

Due to the large parametric space explored here, not all samples could be characterized by STEM. Only the most promising samples towards smaller sizes based on UV-vis measurement as well as illustrative samples of some conditions. In particular, we could not always performed all characterization timely and therefore we indicate here the time elapsed between the synthesis and the STEM analysis and/or UV-vis. We are aware that it is very uncommon to report in so many details the specific of the samples. However, we believe that by doing so we comply with transparency. Note that most samples were relatively stable over time as detailed along the article and stressed in **section S6. Stability**. Also for transparency, we stress which samples were obtained using light and those prepared in the dark, as detailed above and summarized in **Table S3**.

The STEM analysis tends to show that using dark conditions leads to samples with smaller size distribution, as expected from UV-vis data and results detailed above, although smaller size Ag NPs are obtained when the alcohol content increases using $AgNO_3$, EG and a NaOH/Au molar ratio of 4, entries 1-2-3 in **Table S3**, see also **Figure S9**. Below are complementary STEM data to those given in **Figure S4**.

4.1. Further STEM data

Ethanol - 30 v.% - 4 - $AgNO_3$ - L - D2

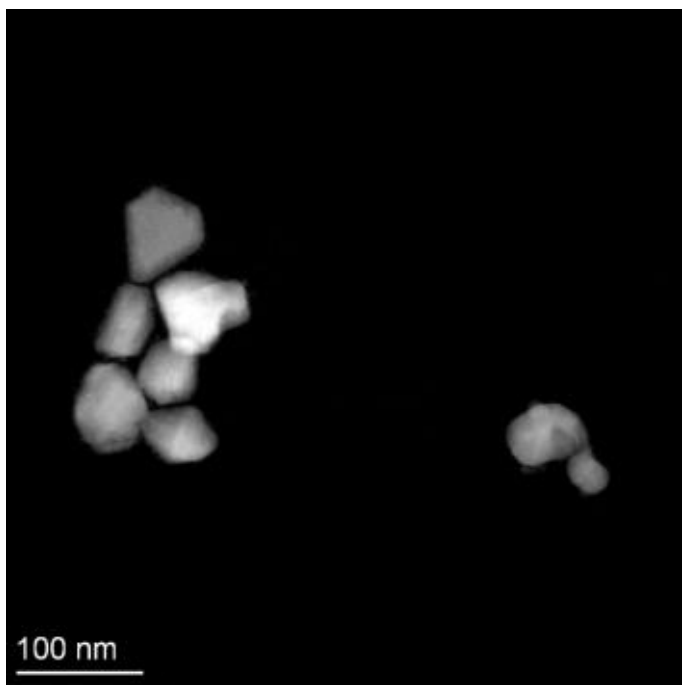

EG - 10 v.% - 4 - $AgNO_3$ - L - D2

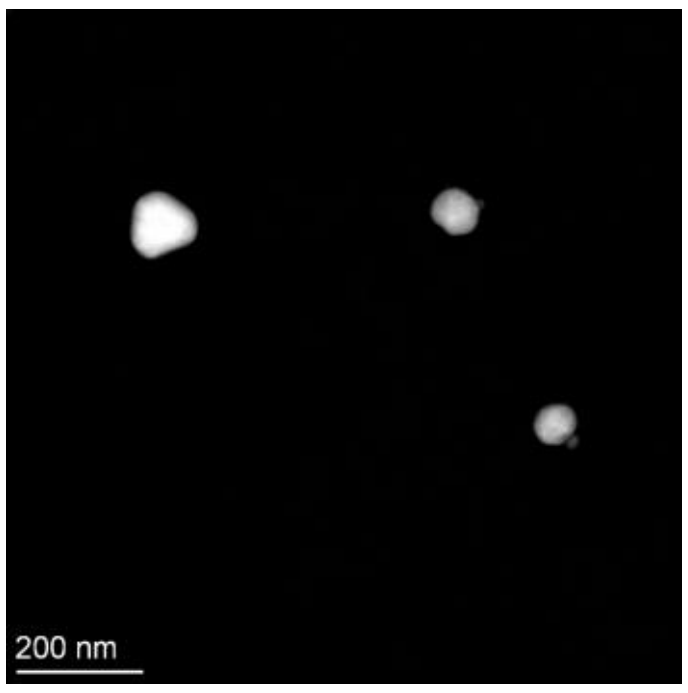

EG - 30 v.% - 4 - $AgNO_3$ - L - D2

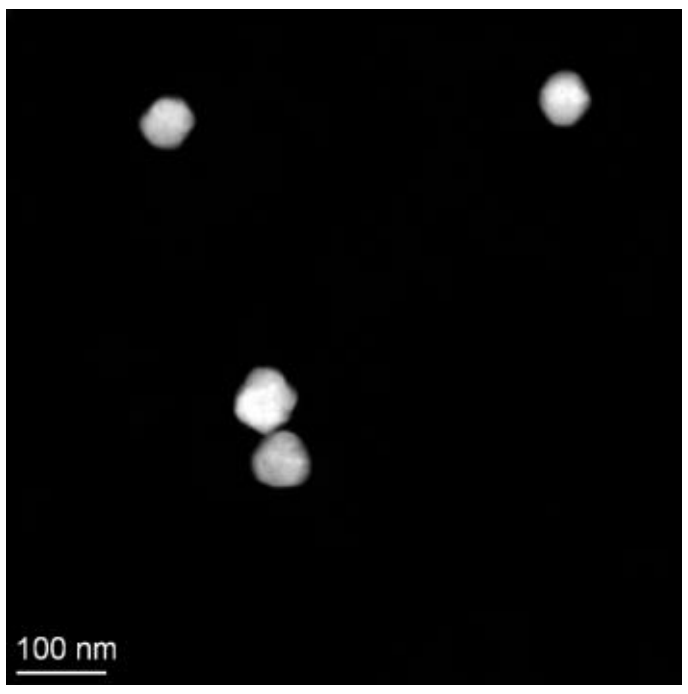

EG - 45 v.% - 4 - $AgNO_3$ - L - D2

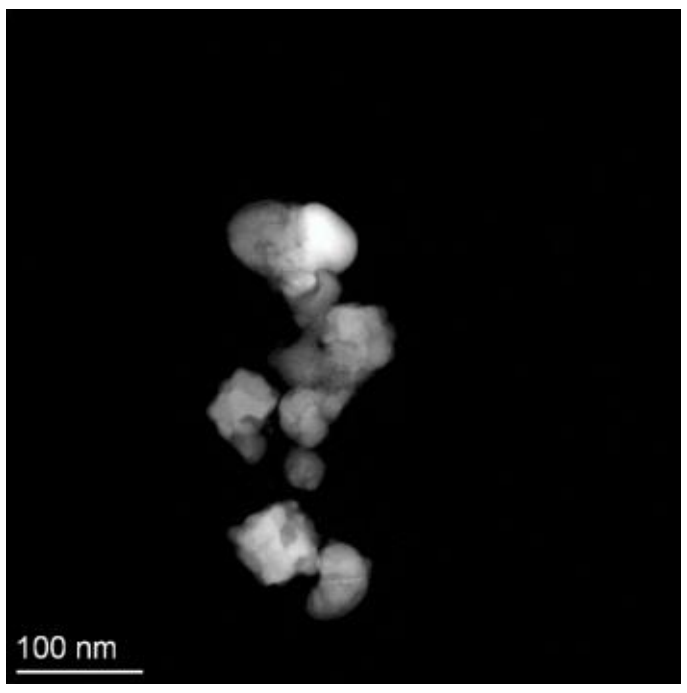

EG - 30 v.% - 4 – $AgClO_4$ - L – D1

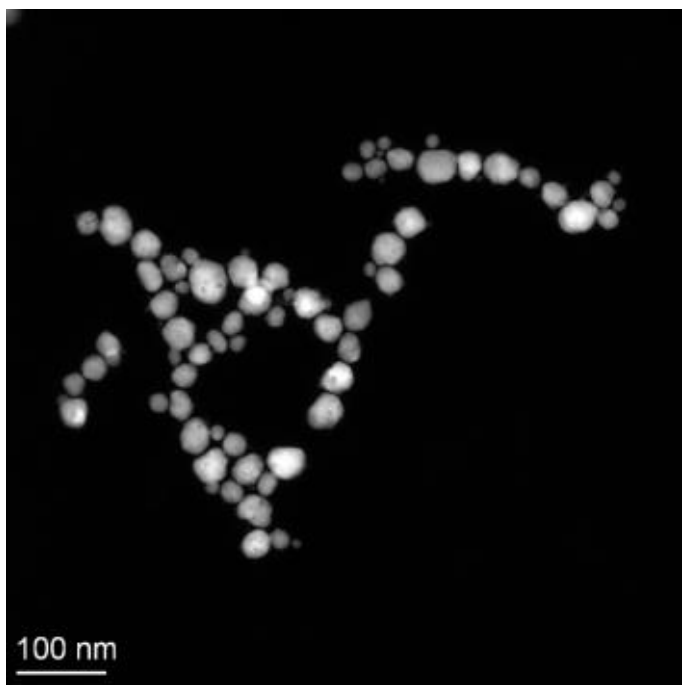

Figure S9. Illustrative STEM micrographs for samples obtained using different conditions, as indicated. The indications given are from left to right: Alcohol – v.% of alcohol – NaOH/Ag molar ratio – Precursor – Light condition for synthesis – Day after synthesis when the grid was prepared for STEM. All samples were prepared with 0.25 mM equivalent of Ag for the precursor and stored at RT in the dark. L means that the samples were prepared in the light. DX indicates the number X of days after synthesis when the grid was prepared for STEM analysis.

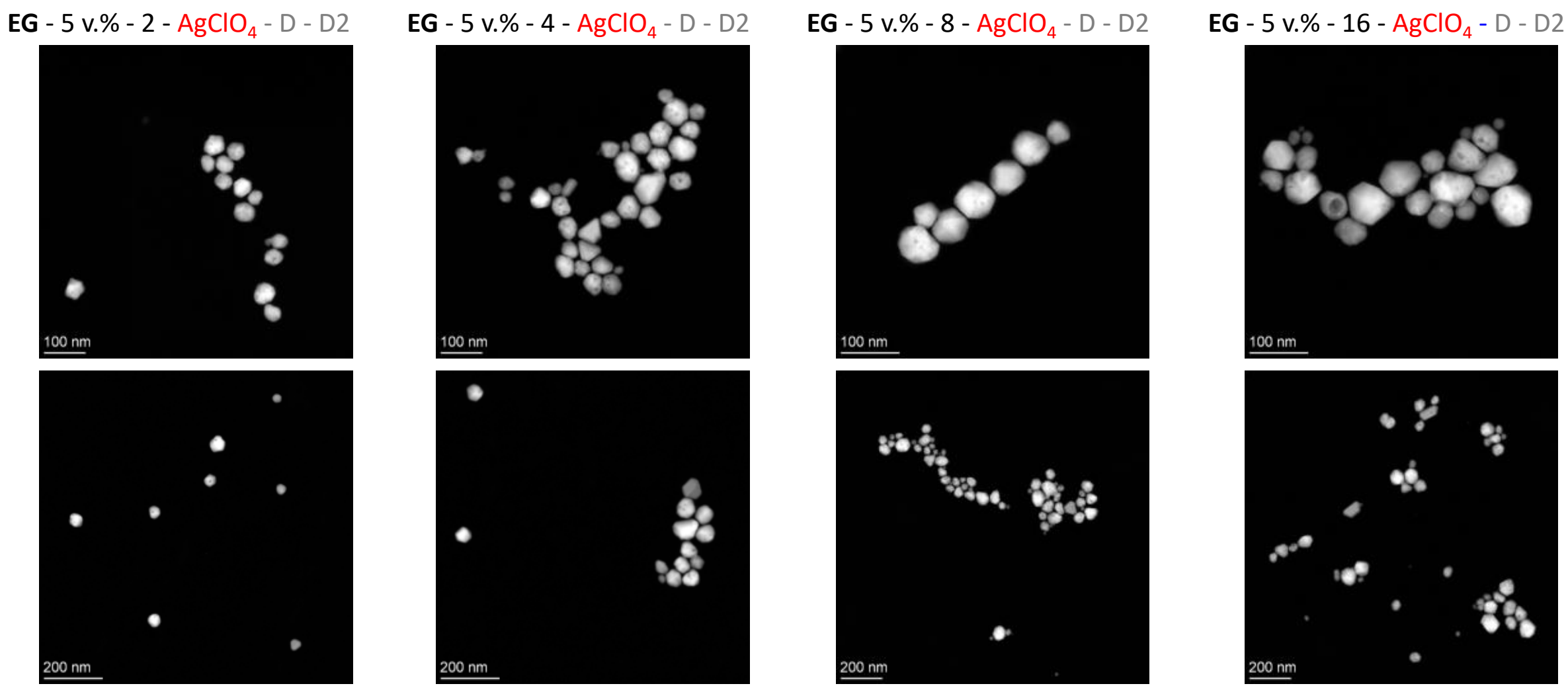

Figure S10. Illustrative STEM micrographs at different magnifications for samples obtained using different conditions, as indicated. The indications given are from left to right: Alcohol – v.% of alcohol – NaOH/Ag molar ratio – Precursor – Light condition for synthesis – Day after synthesis when the grid was prepared for STEM. All samples were prepared with 0.25 mM equivalent of Ag for the precursor and stored at RT in the dark. D means that the samples were prepared in the dark. DX indicates the number X of days after synthesis when the grid was prepared for STEM analysis.

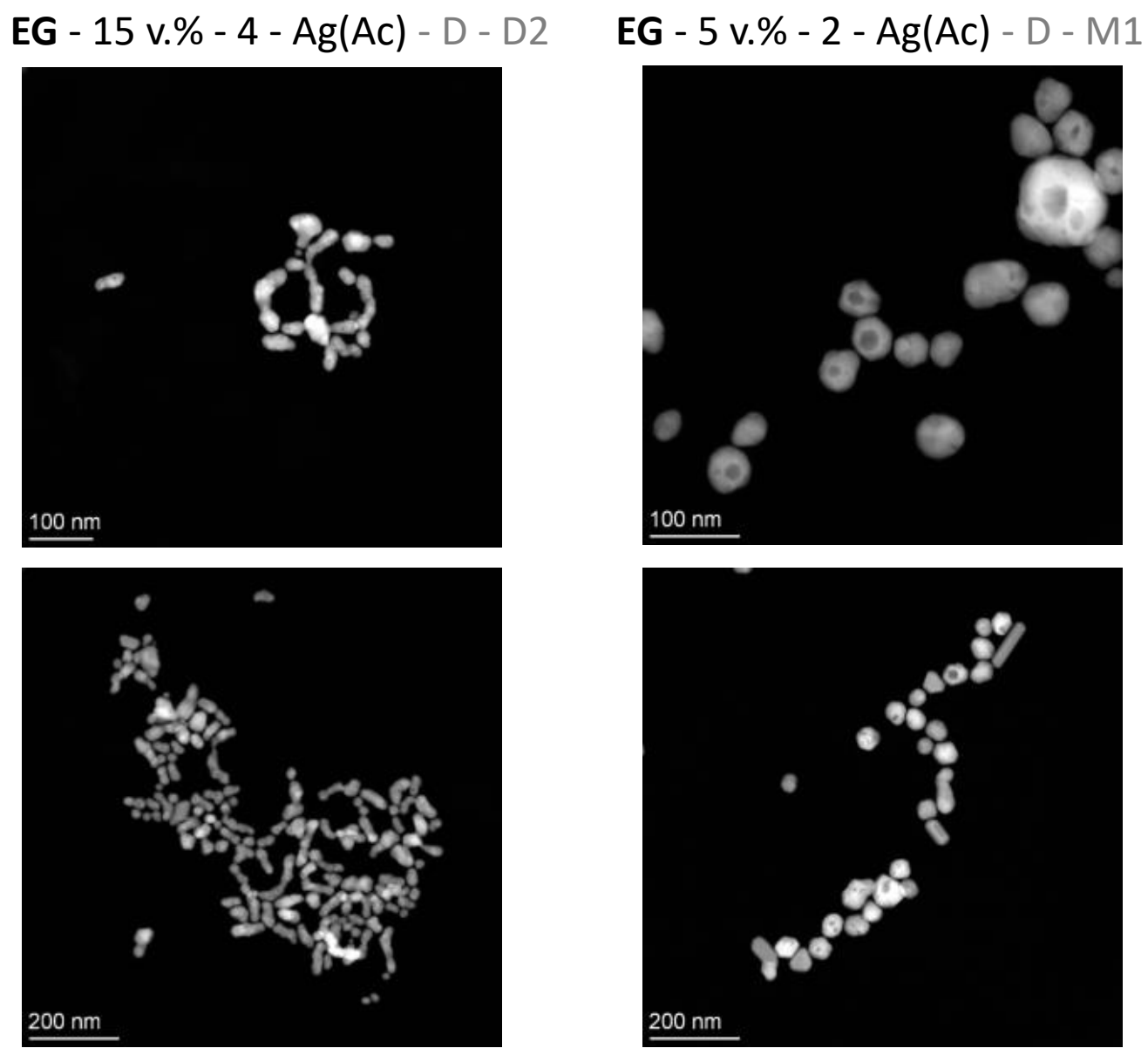

Figure S11. Illustrative STEM micrographs at different magnifications for samples obtained using different conditions, as indicated. The indications given are from left to right: Alcohol – v.% of alcohol – NaOH/Ag molar ratio – Precursor – Light condition for synthesis – Day after synthesis when the grid was prepared for STEM. All samples were prepared with 0.25 mM equivalent of Ag for the precursor and stored at RT in the dark. D means that the samples were prepared in the dark. MX indicates the number of X months after synthesis when the grid was prepared for STEM analysis.

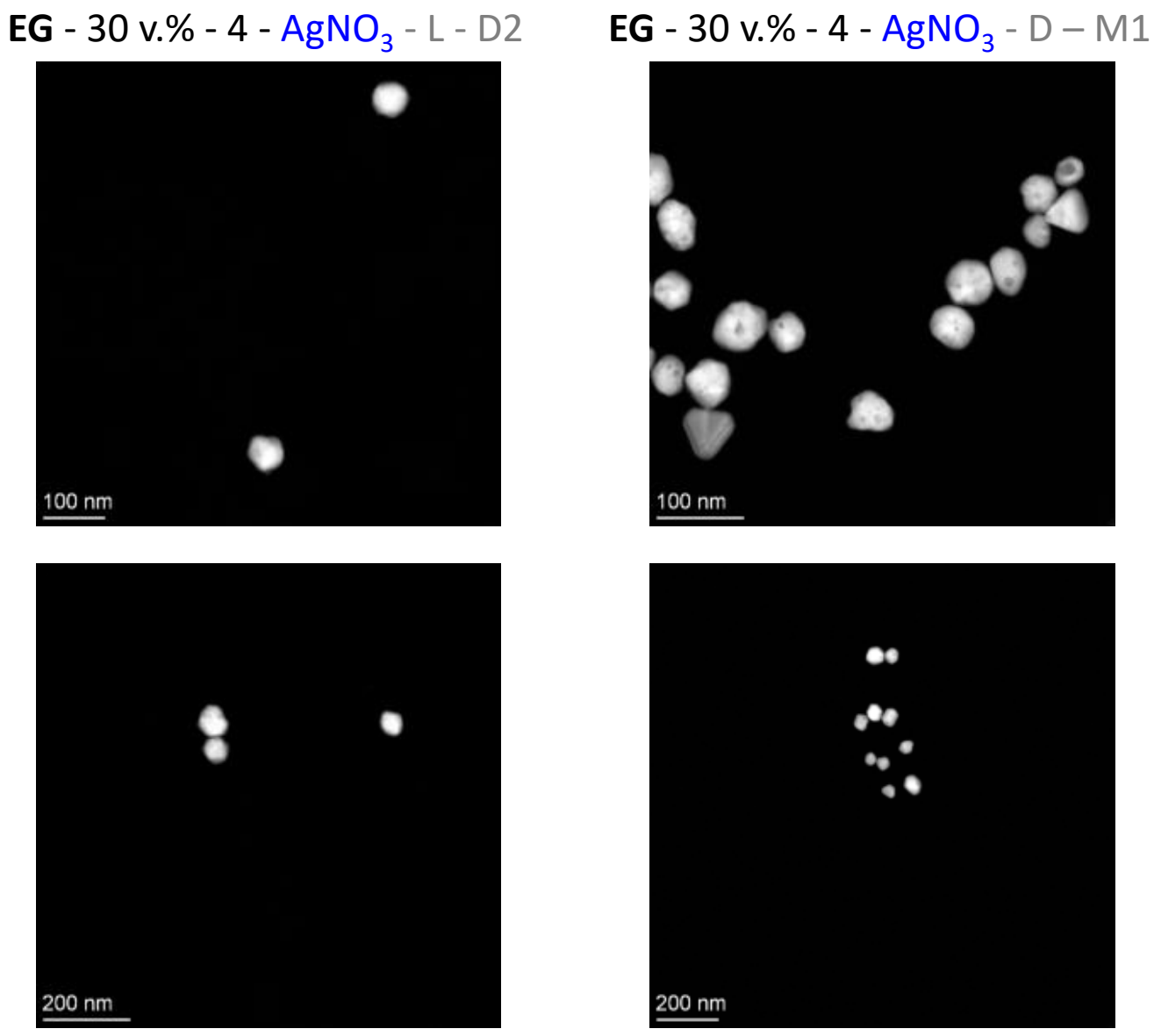

Figure S12. Illustrative STEM micrographs at different magnifications for samples obtained using different conditions, as indicated. The indications given are from left to right: Alcohol – v.% of alcohol – NaOH/Ag molar ratio – Precursor – Light condition for synthesis – Day after synthesis when the grid was prepared for STEM. All samples were prepared with 0.25 mM equivalent of Ag for the precursor and stored at RT in the dark. D means that the samples were prepared in the dark. L means that the samples were prepared in the light. DX and MX indicate the number X of days or months, respectively, after synthesis when the grid was prepared for STEM analysis.

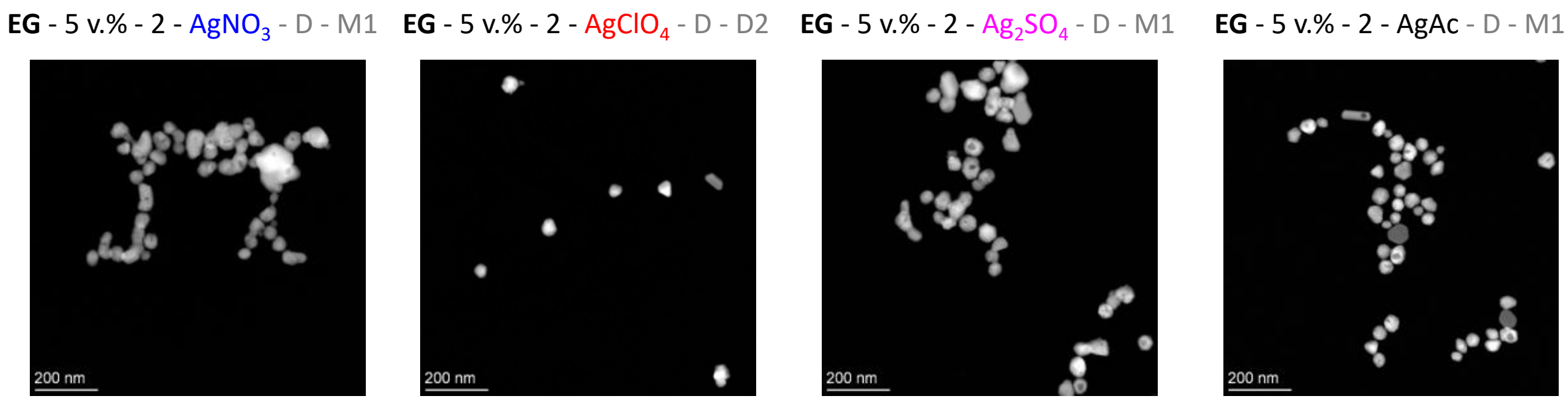

Figure S13. Illustrative STEM micrographs for samples obtained using different conditions, as indicated. The indications given are from left to right: Alcohol – v.% of alcohol – NaOH/Ag molar ratio – Precursor – Light condition for synthesis – Day after synthesis when the grid was prepared for STEM. All samples were prepared with 0.25 mM equivalent of Ag for the precursor and stored at RT in the dark. D means that the samples were prepared in the dark. L means that the samples were prepared in the light. DX and MX indicated the number X of days or months, respectively, after synthesis when the grid was prepared for STEM analysis.

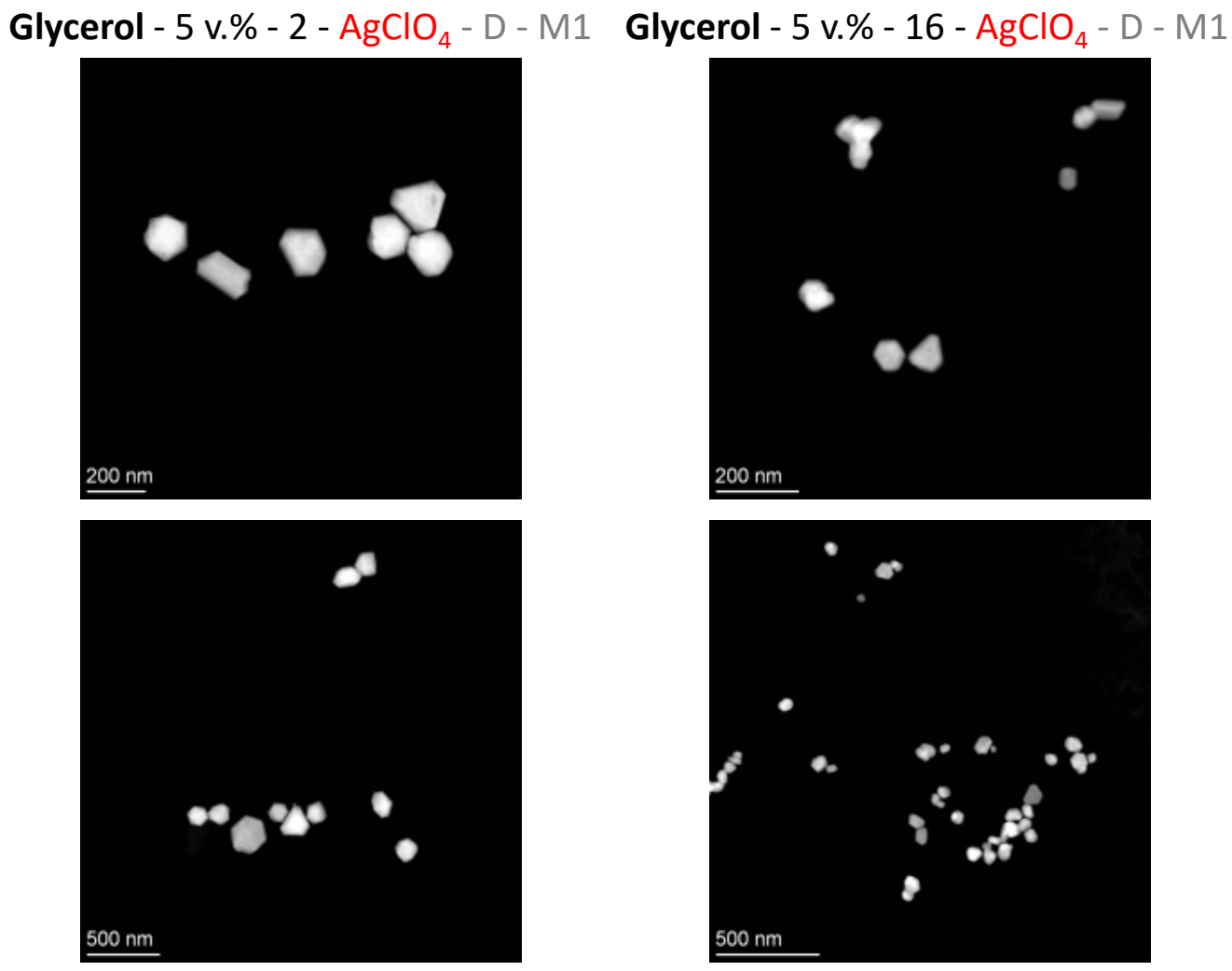

Figure S14. Illustrative STEM micrographs at different magnifications for samples obtained using different conditions, as indicated. The indications given are from left to right: Alcohol – v.% of alcohol – NaOH/Ag molar ratio – Precursor – Light condition for synthesis – Day after synthesis when the grid was prepared for STEM. All samples were prepared with 0.25 mM equivalent of Ag for the precursor and stored at RT in the dark. D means that the samples were prepared in the dark. MX indicate the number X of months after synthesis when the grid was prepared for STEM analysis.

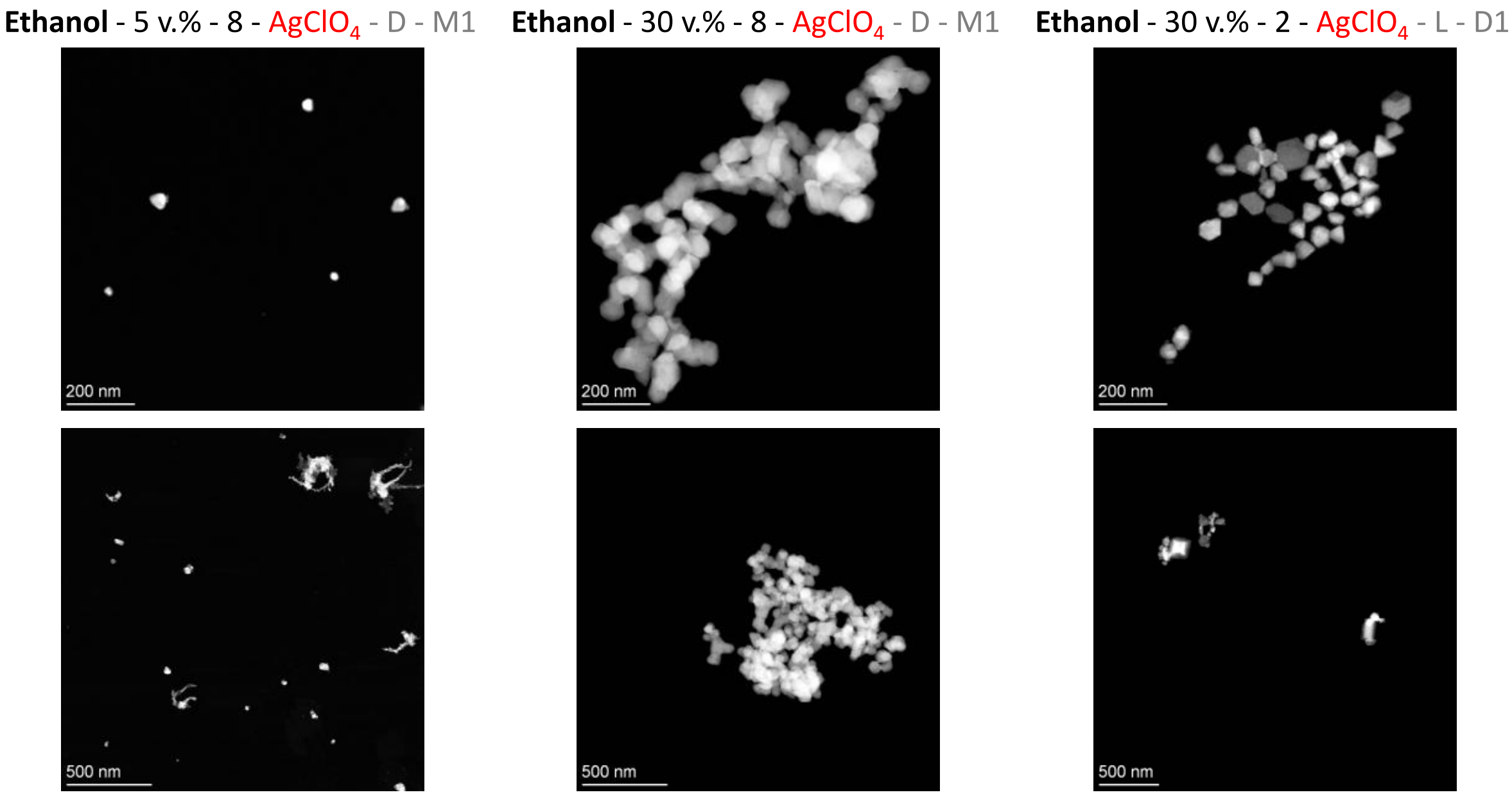

Figure S15. Illustrative STEM micrographs at different magnifications for samples obtained using different conditions, as indicated. The indications given are from left to right: Alcohol – v.% of alcohol – NaOH/Ag molar ratio – Precursor – Light condition for synthesis – Day after synthesis when the grid was prepared for STEM. All samples were prepared with 0.25 mM equivalent of Ag for the precursor and stored at RT in the dark. D means that the samples were prepared in the dark. L means that the samples were prepared in the light. DX and MX indicate the number X of days or months, respectively, after synthesis when the grid was prepared for STEM analysis.

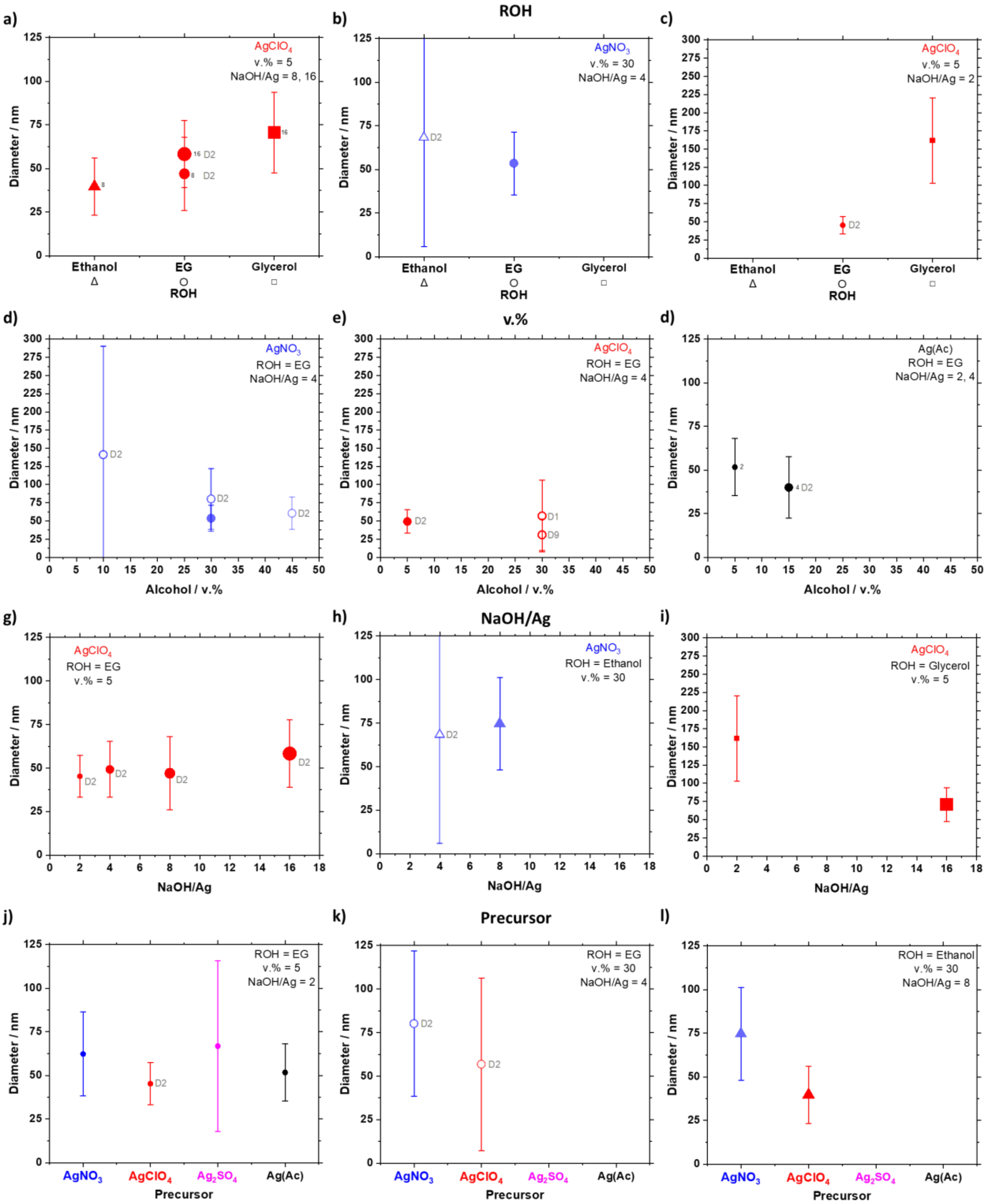

Figure S16. Sizes retrieve from STEM for different samples. (a-c) Influence of the alcohol (ROH) used. (d-f) Influence of the v.% of alcohol. (g-i) Influence of the NaOH/Au molar ratio at a fixed concentration of Ag at 0.25 mM. (j-l) Influence of the Ag precursor. All experiments were performed using 0.25 mM Ag. Across all panels: the type of alcohol used is coded by the shape of the data point: Ethanol (Δ), EG (o), and glycerol (□), see (a-c); the content of alcohol is coded by the transparency of the color of the data point where more transparent data points correspond to samples more alcohol, see (d-f); the NaOH/Au molar ratio is coded by the size of the data point

where larger data point correspond to higher NaOH/Au molar ratio, see (g-i); the precursor used is coded by the color of the data point see (j-l). Unless otherwise specified the grid was prepared a month after synthesis (and storage at RT in a drawer in aluminum), otherwise D1, D2 or D9 indicate the number of day after synthesis the sample was prepared, 1, 2 or 9 days, respectively. Furthermore, some samples were prepared in light conditions (data point with no color in the middle, e.g. ○) and other in dark conditions (data point with an inner color, e.g. ●), as indicated. The values in (a) and (d) close to the data points are the related NaOH/Ag molar ratios.

4.2. Further UV-vis data

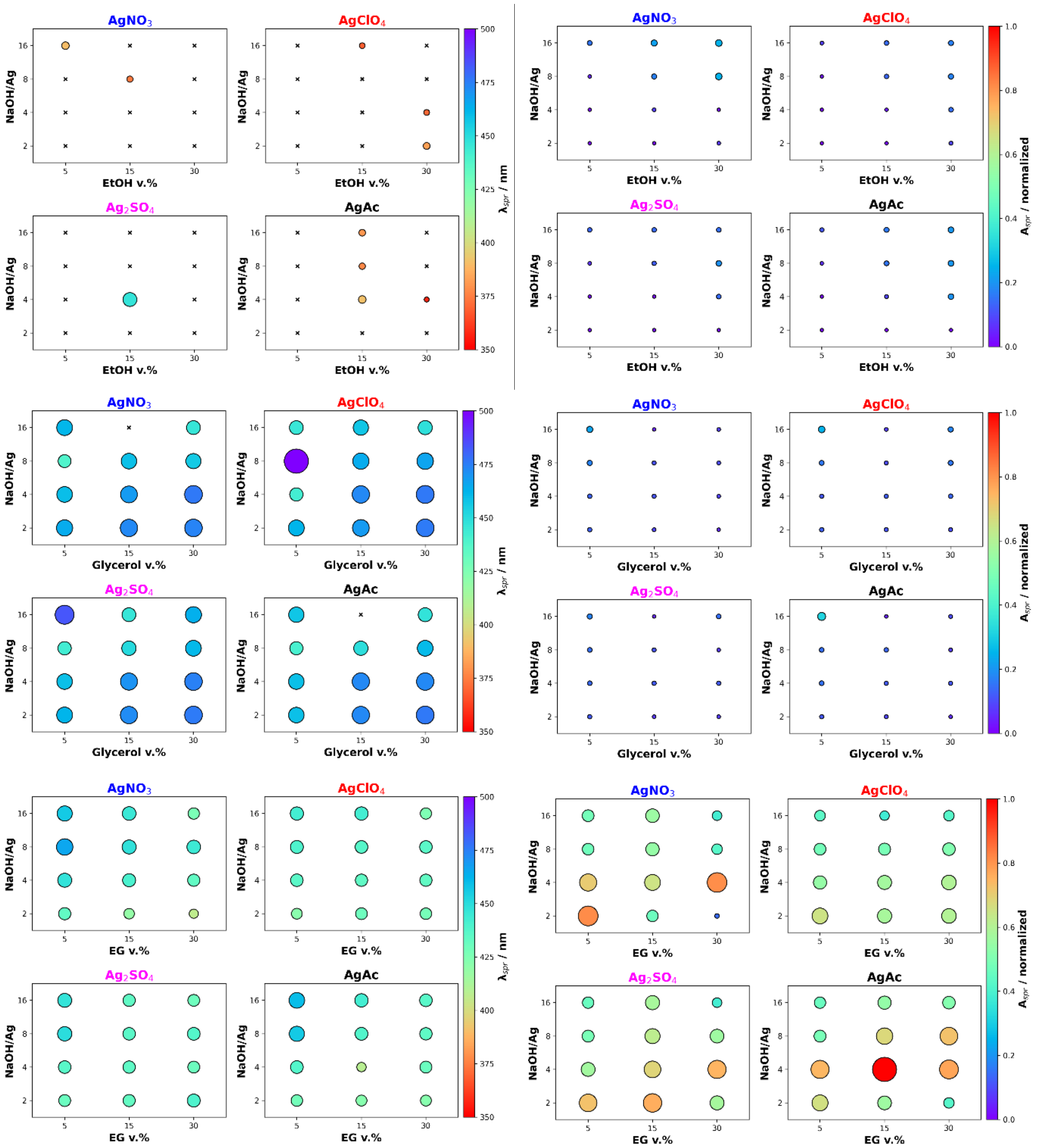

Figure S17. Bubble pots of (left-hand side column) λ_{spr} values and (right-hand side column) A_{spr} values (normalized to the maximum of the dataset) for the parametric study performed using different alcohols, different precursors, different contents of alcohols and different base concentrations (NaOH/Ag molar ratio), as indicated. In all cases the concentration of Ag was 0.25 mM. The size of the datapoint is proportional to the value they represent, in addition a color bar is provided to guide the eyes where more red colors are desirable features (indicative a relatively smaller size). The data related to $AgNO_3$ are presented in **Figure 3** of the manuscript for A_{spr}. A X indicates that the experiment was performed but not clear spr features were observed in the UV-vis spectrum indicative of non-stable colloids. Nevertheless an indication of the absorbance is given as A_{spr}.

Given the criteria defined above, it is considered that optimal syntheses are characterized by large size dots with a more orange color for A_{spr} values and smaller size with a more orange color for λ_{spr} values.

Although the samples obtained in EtOH lead to smaller λ_{spr} values the corresponding A_{spr} values are small and in many cases it is actually challenging to identify a proper maximum. When glycerol is used the spr is more pronounced at relatively high λ_{spr} values with still relatively low A_{spr} values. Only the use of EG lead to clear spr at relatively low λ_{spr} values and high A_{spr} values.

Overall, there is no clear benefit to use one precursor or another. There is also no benefit to use large amount of alcohol. Increasing the NaOH/Ag molar ratio does not lead to improved samples and the smaller NP sizes tend to be obtained at low alcohol content and low NaOH/Ag ratio. In EG at low v.% there is a tendency to observe lower λ_{spr} values and higher A_{spr} values as the NaOH/Ag molar ratio decreases.

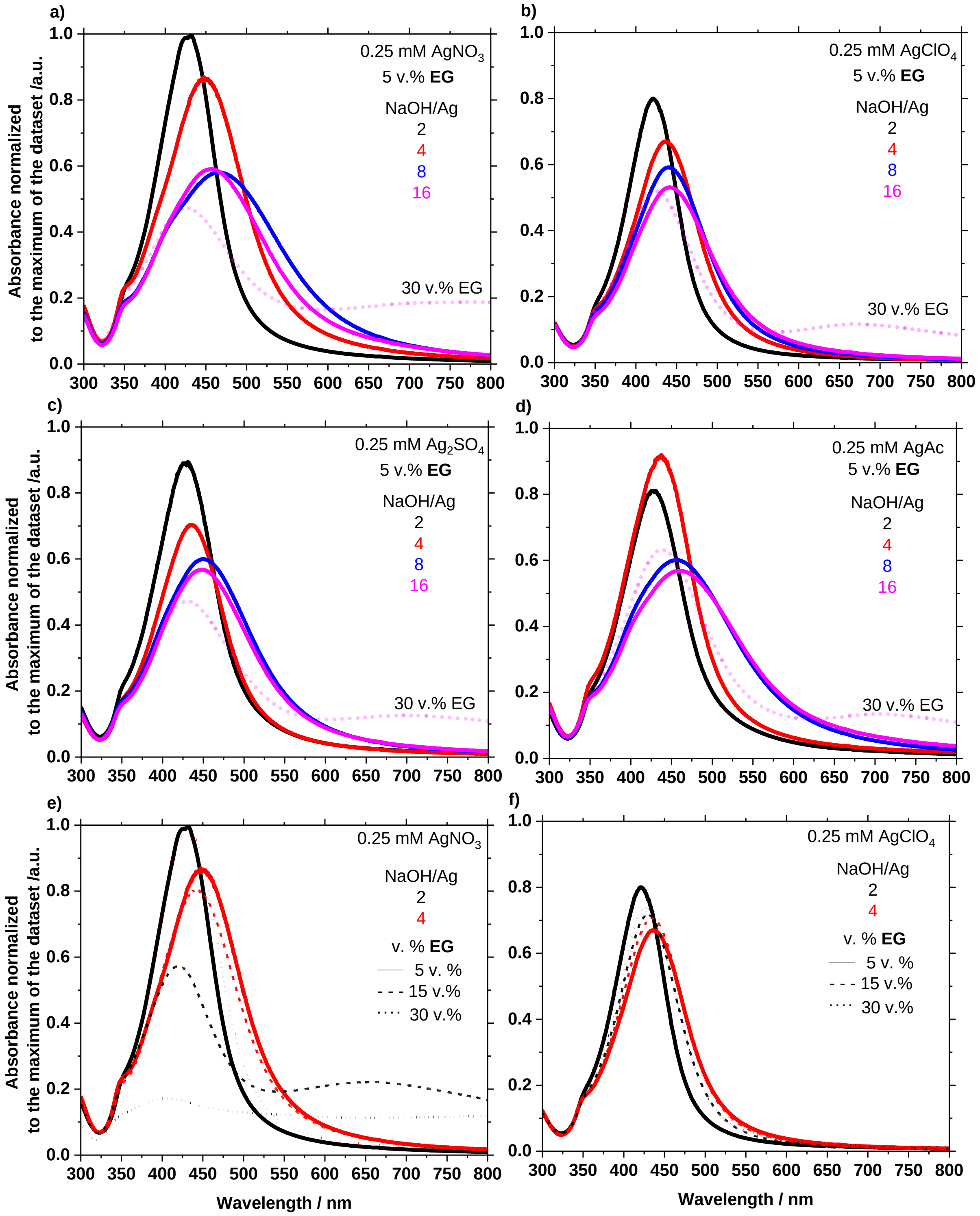
a)
0.25 mM $AgNO_3$
5 v.% **EG**
NaOH/Ag
2
4
8
16
30 v.% EG
b)
0.25 mM $AgClO_4$
5 v.% **EG**
NaOH/Ag
2
4
8
16
30 v.% EG
c)
0.25 mM Ag_2SO_4
5 v.% **EG**
NaOH/Ag
2
4
8
16
30 v.% EG
d)
0.25 mM AgAc
5 v.% **EG**
NaOH/Ag
2
4
8
16
30 v.% EG
e)
0.25 mM $AgNO_3$
NaOH/Ag
2
4
v. % **EG**
5 v. %
15 v.%
30 v.%
f)
0.25 mM $AgClO_4$
NaOH/Ag
2
4
v. % **EG**
5 v. %
15 v.%
30 v.%
Absorbance normalized to the maximum of the dataset /a.u.
Wavelength / nm

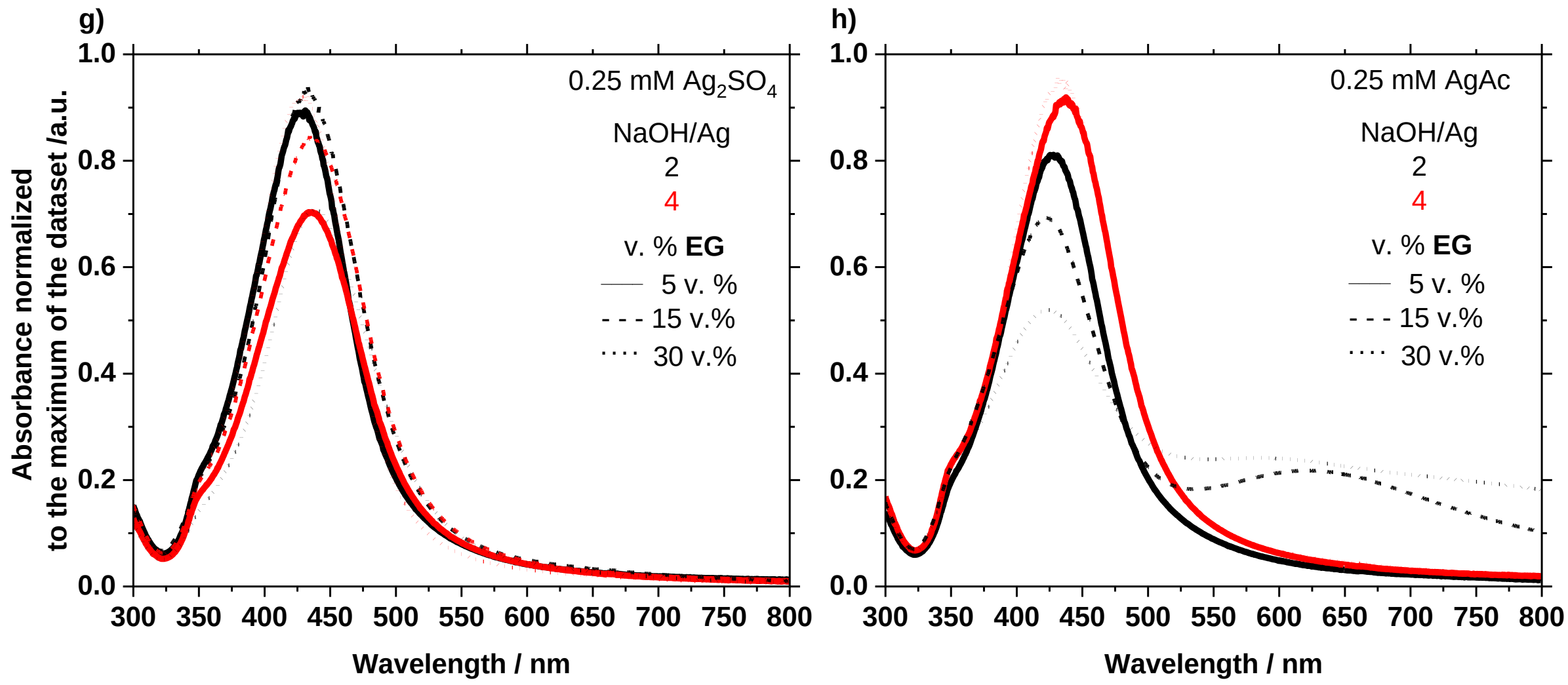

Figure S18. UV-vis spectra for samples prepared under different conditions, as indicated. In all cases 0.25 mM Ag and EG was used. (a) reproduces the data presented in **Figure 3f** to facilitate the comparison.

Figure S18 illustrates the relative size control when the NaOH/Ag molar ratio increases but also the little benefits to increase the amount of EG.

4.3. Influence of alcohol

Based on the results above, the use of methanol (not shown) or ethanol does not easily lead to small size Ag NPs. The UV-vis spectra of Ag NPs obtained in methanol or ethanol (across different NaOH concentration, alcohol v.%, and for different precursors) typically do not show a well-defined λ_{spr} and show relatively low A_{spr} values. In most cases the solution show grey-ish precipitate at the bottom of the reactor. For such samples, no UV-vis signal could be recorded (flat lines). Illustrative UV-spectra of samples obtained in ethanol when a UV-vis spectra could be measured are given in **Figure 3a**. It is therefore concluded that methanol or ethanol are not a suitable solvent to obtain Ag NPs. Methanol is furthermore a rather toxic chemical,[53] compared to ethanol, EG or glycerol, and was therefore not considered further.

The use of glycerol is commonly reported for the synthesis of Ag NPs, although the use of stabilizers is often preferred. The suitability of glycerol comes from two main factors: (i) under alkaline conditions the formation of alkoxides will favors the reduction of the Ag precursors[30] and (ii) the high viscosity of glycerol might play a role in the stabilization of the NPs. Glycerol-mediated syntheses lead to better defined spr features (compared to the case of ethanol) and moderate A_{spr} values across the all parameter, space which suggests that glycerol-mediated syntheses successfully lead to Ag nanomaterials. However, the features in the UV-vis spectra are relatively broad as illustrated in **Figure 3b**.

Finally, the use of EG leads to the best defined spr features with an easily identified λ_{spr} values and higher A_{spr} values, as illustrated in **Figure 3c** and for the data above. The related dispersions have a yellow color.

As an illustration, from STEM data in **Figure S16a-c**, it can be concluded that the size obtained depends on the NaOH/Ag molar ratio. The benefit of EG over glycerol to obtain small size Ag NPs at low alcohol content (5 v.%) and low amount of base (NaOH/Au = 2) is exemplified in **Figure S16c**. There is a trend that to obtain smaller size Ag NPs, using glycerol requires higher amount of NaOH: For 5 v.% and glycerol used, with $AgClO_4$, smaller NPs are obtained using NaOH/Ag molar ratio of 16 vs. 2, see **Figure S16i**.

The reasons why such strong difference are observed when EG is used compared to the other alcohols considered in this study is attributed to the different properties of the organic molecules,[38] and a definite answer remain beyond the scope of this first report.

4.4. Influence of alcohol content

Overall, for a given alcohol, a given precursor and a given concentration of NaOH, the amount of alcohol does not have a strong influence on the resulting UV-vis features. This result suggests that only low amount of alcohol between 5-10 v.% are enough to ensure the formation of Ag NPs. This is a positive feature to develop green(er) syntheses.

As an illustration, from STEM data in **Figure S16d-f**, it can be conducted that overall the size obtained does not depend much on the amount of alcohols, in particular if the reaction is performed in the dark.

4.5. Influence of NaOH/Ag molar ratio

Overall, for a given alcohol, a given precursor and a given amount of alcohol, the concentration of NaOH does not have a strong influence on the resulting UV-vis features. In EG there is a tendency to observe that lower NaOH/Ag molar ratio leads to higher A_{spr} and smaller λ_{spr} values. This result suggests that low amount of base are enough to ensure the formation of Ag NPs. This is a positive feature to develop green(er) syntheses. Note that without base of for concentration lower than those investigated, the reaction does not seem to proceed, in agreement with slow kinetics (over days) observed in the absence of base.[28]

As an illustration, from STEM data in **Figure S16g-i**, it can be conducted that the size obtained does not depend much on the NaOH/Ag molar ratio when EG is used. There is a trend to obtain larger NPs when the NaOH/Ag molar ratio increases when $AgClO_4$ is used. For glycerol, the NaOH/Ag molar ratio has a strong effect, see **Figure S16i**, where smaller Ag NPs are then obtained at higher NaOH/Ag ratios.

4.6. Influence of precursors

Overall, different precursors, all containing Ag in the oxidation degree +I were screened. It can be that the different precursors have different redox properties that might lead to different Ag NPs. It can also be expected that the anions from the precursor can play different roles in the stability of the Ag NPs.

Overall, for a given alcohol, a given amount of alcohol, a given concentration of NaOH, the nature of the precursor does not have a strong influence on the resulting UV-vis features. There is an overall trend to observe slightly lower λ_{spr} values when $AgClO_4$ is used with glycerol compared to other precursor. In EG, it seems that Ag_2SO_4 leads to slightly lower λ_{spr} values and higher A_{spr} values. The prices of the precursor depends on the grade and the scale at which the precursor is used, but it is positive features that four common precursors are suitable to obtain Ag NPs in EG-water mixtures. It in particular encouraging the inorganics salts ($AgNO_3$, $AgClO_4$, Ag_2SO_4) lead to Ag NPs given that organic based molecules (Ac) are often derived from fossil fuels.

4.7. Influence of temperature

A focus was here given to RT processes to develop green(er) synthesis of Ag NPs. Attempts were also performed to tune the temperature of the synthesis (up to 40 °C) without significant improvements on the resulting optical properties of the Ag NPs.

4.8. Comments on size and shape control

The results presented suggest that some degree of size control is possible by the present approach. An extra knob to explore for a finer control towards this endeavor would be the concentration of precursor used, but is beyond the scope of this first report that establishes that stable surfactant-free colloidal Ag NPs can be obtained in particular using alkaline EG as source of reducing agent. Shape and/or structure control are important for materials and are limited for surfactant-free synthesis although the more detailed study of the use of different precursors such as AgAc, see **Figure S11** for some elongated structures, could lead to different shapes and/or more faceted NPs as observed on some samples. Although this is not the main focus of this first report, this suggest some degree of shape and structure control might be achievable in the future by the careful selection of the experimental conditions.

4.9. Conclusion

The synthesis is robust to several parameters and conveniently performed at relatively low NaOH/Ag molar ratios around 2-4, with different Ag precursors at a concentration of 0.25 mM, in water-alcohol mixtures with low alcohol contents, e.g. around 5-10 v.%. The synthesis is fast and typically completed in ca. 30 min (see **Figure 4**). It is ideally performed in the dark to ensure the long-term stability of the ca. 50 nm Ag NPs over several weeks.

As a note: attempts to perform synthesis in EG (pure EG, 0.10 mM $AgNO_3$, 5 mM NaOH, NaOH/Ag molar ratio of 50) without surfactant led to broadening of the UV-vis spr peaks overtime.[29] The peak show significant absorption between 500 and 700 nm and a shoulder is rather pronounced with nearly a second maximum around 550 nm. In the present case of syntheses performed with EG and a NaOH/Ag molar ratio of 16, there is no always such shoulder and long term stability is achieved but a second maximum was typically observed for 30 v.% EG, see **Figure S18**. The difference can be attributed to (i) our focus on syntheses performed in dark conditions and (ii) our focus on minimizing the amount of chemicals (EG and base).

S5. Kinetics

Given the results presented in the article in **Figure 4**, there is room to optimize the time of stirring here preferentially performed for 2 hours but it could probably be reduced to 30 minutes.

An important last piece of information retrieved from these kinetic data is on the formation mechanism of the Ag NPs. The classical nucleation theory will dictate that the rapid formation of several seeds followed by a slow growth will be a key to obtain small size NPs. However, the smallest size NPs were obtained using EG where the reaction is the slowest. This could be explained by recently reported model where the stabilization of the NPs plays a role maybe as important as the formation mechanism, [54] especially here in the case of surfactant-free Ag NPs. While it remains challenging to correlate the formation pathway observed from UV-vis to the properties of the alcohols used at this stage, the results nevertheless show the opportunities that the surfactant-free Ag NPs at RT can offer to ultimately gain a better understanding of NP formation and refine current models.

S6. Stability

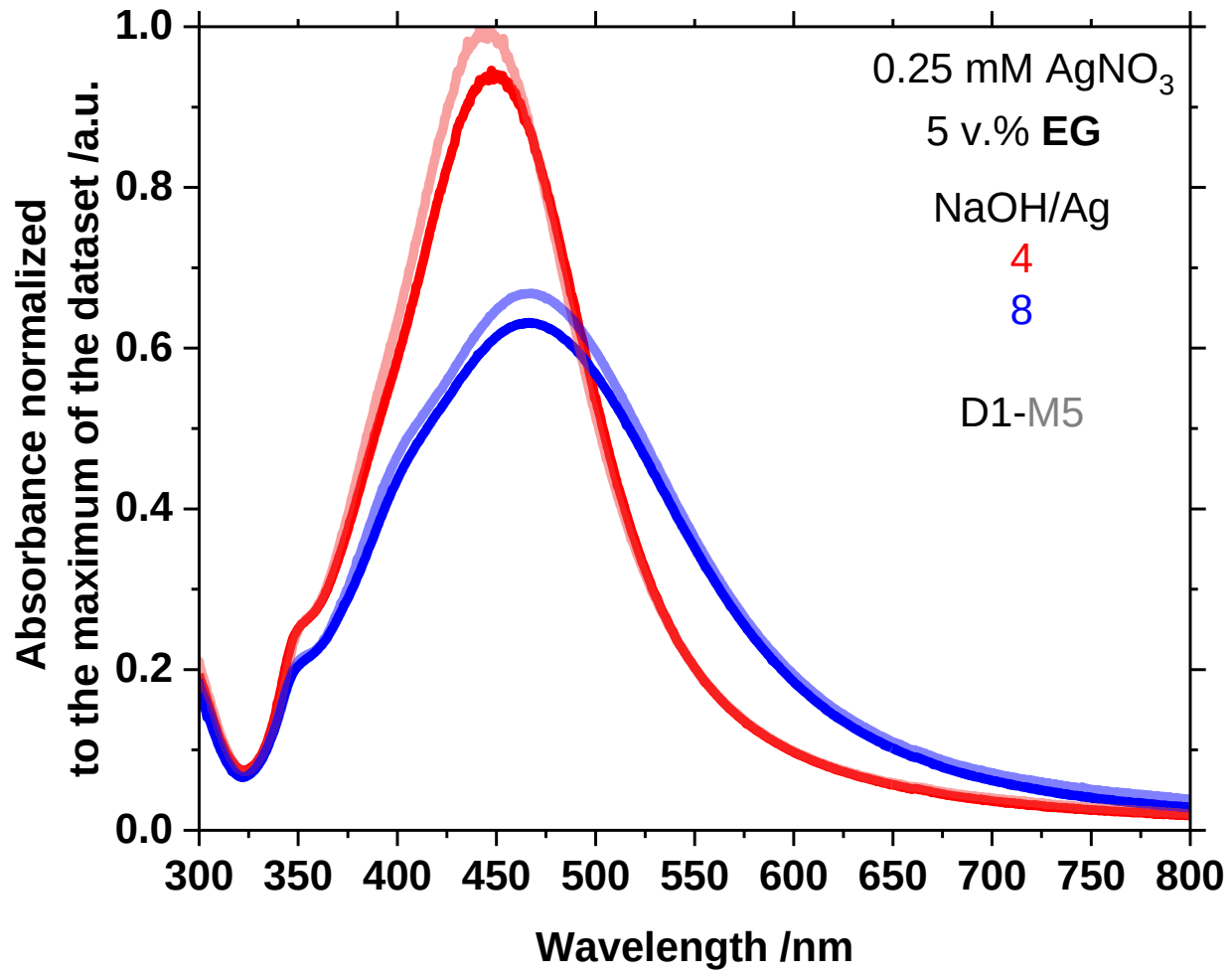

Figure S19. Illustrative UV-vis spectra showing the stability of the surfactant-free colloids for samples obtained under different conditions, as indicated, and for spectra measured the day after synthesis (D1, darker) or 5 months after synthesis (M5, lighter) with storage in the dark and a RT.

Despite being surfactant-free, the synthesis leads to colloids stable for months.

S7. Proof of concept for catalysis

As a proof of concept of the relevance of the NPs for catalysis, we evaluated if the Ag NPs were active for the 4-nitrophenol reduction based on the protocols reported previously.[45]

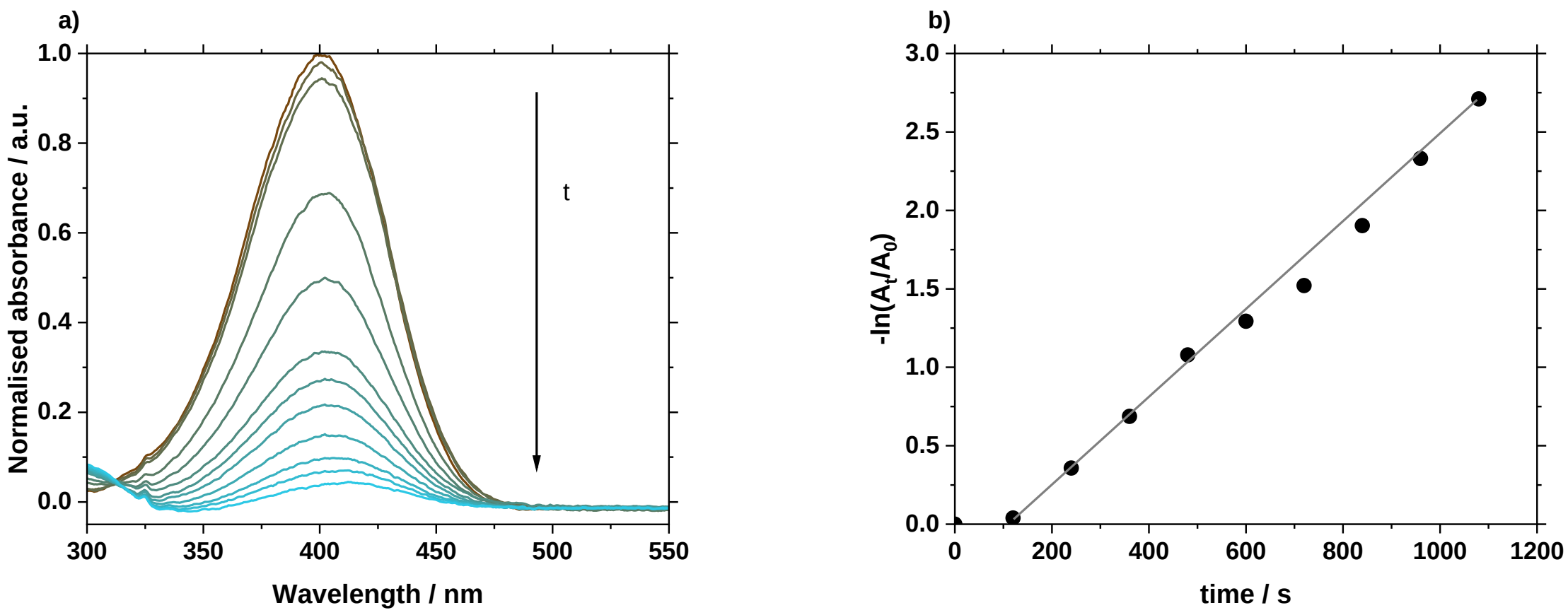

Figure S20. Proof of concept of the catalytic activity of the Ag NPs for catalysis with the example of the 4-nitrophenol reduction. (b) Illustration of the decrease of absorbance at 400 nm over time. (b) $-\ln(A_t/A_0)$ where A_0 is the absorbance at t = 0 s and A_t the absorbance at 400 nm at a given time t. In this case NPs prepare using 0.25 mM $AgClO_4$, 2 mM NaOH, 5 v.% EG were used, ca. 45 nm in diameter.

The induction period observed at the beginning of the reaction (after addition of the Ag NPs) is due to oxygen because the solution was not purged with N_2. The estimated k_{app} value is 0.00257 s^{-1} for a TOF of 38 h^{-1}.

S8. Comparison gold *vs.* silver nanoparticles

Both Au and Ag NPs, due to the relatively close redox potentials and chemistry of the precursors used, can be obtained by relatively similar synthetic procedures. However (crucial) differences make it challenging to transfer the recipe developed for one to the other, in general,[55] and for the specific synthetic approach developed here, as summarized in **Table S4**.

Table S4. Differences between Au and Ag NP synthesis using RT surfactant-free syntheses in alkaline water-alcohol mixtures (selected main parameters). The synthesis for Au NPs is reported elsewhere.[5, 36, 38]

	**[Precursor]*** mM	**[NaOH]** mM	**NaOH**	**ROH**	**v.% ROH**	**Speed**	**Light**	**Stirring Shaking**
**Au NPs**	0.5	4	Strong effect on size	Ethanol[5, 36] EG[5, 17, 36] Glycerol[5, 36]	20-30 optimal	hour(s)	Strong effect on size: lower wavelengths lead to smaller NPs[38]	Moderate effect
**Ag NPs**	0.25	0.5	Moderate effect on size	EG most suitable	< 20 optimal	minutes < hour	Larger NPs in the dark but better stability	Proper stirring seems more important

* The choice is mainly driven by the ability to measure the (many) samples directly by UV-vis without saturating the detector and therefore without having to dilute all the (many) samples. However, note that both 0.5 mM for Au NPs and 0.25 mM for Ag are on the high end of reported precursor concentration for water-based syntheses, especially considering that no stabilizers are used, see **Table S1**.

S9. References

(1) Losch, P.; Huang, W. X.; Goodman, E. D.; Wrasman, C. J.; Holm, A.; Riscoe, A. R.; Schwalbe, J. A.; Cargnello, M. Colloidal nanocrystals for heterogeneous catalysis. *Nano Today* **2019**, *24*, 15-47. DOI: 10.1016/j.nantod.2018.12.002.
(2) Quinson, J.; Jensen, K. M. Ø. From platinum atoms in molecules to colloidal nanoparticles: A review on reduction, nucleation and growth mechanisms. *Advances in Colloid and Interface Science* **2020**, *286*, 102300. DOI: 10.1016/j.cis.2020.102300.
(3) Thanh, N. T. K.; Maclean, N.; Mahiddine, S. Mechanisms of Nucleation and Growth of Nanoparticles in Solution. *Chemical Reviews* **2014**, *114* (15), 7610-7630. DOI: 10.1021/cr400544s.
(4) De Souza, C. D.; Nogueira, B. R.; Rostelato, M. Review of the methodologies used in the synthesis gold nanoparticles by chemical reduction. *Journal of Alloys and Compounds* **2019**, *798*, 714-740. DOI: 10.1016/j.jallcom.2019.05.153.
(5) Quinson, J.; Aalling-Frederiksen, O.; Dacayan, W. L.; Bjerregaard, J. D.; Jensen, K. D.; Jørgensen, M. R. V.; Kantor, I.; Sørensen, D. R.; Theil Kuhn, L.; Johnson, M. S.; et al. Surfactant-free colloidal syntheses of gold-based nanomaterials in alkaline water and mono-alcohol mixtures. *Chemistry of Materials* **2023**, *35* (5), 2173–2190. DOI: 10.1021/acs.chemmater.3c00090.
(6) Quinson, J. On the Importance of Fresh Stock Solutions for Surfactant-Free Colloidal Syntheses of Gold Nanoparticles in Alkaline Alcohol and Water Mixtures. *Inorganics* **2023**, *11* (4), 140. DOI: 10.3390/inorganics11040140.
(7) El Amri, N.; Roger, K. Polyvinylpyrrolidone (PVP) impurities drastically impact the outcome of nanoparticle syntheses. *Journal of Colloid and Interface Science* **2020**, *576*, 435-443. DOI: 10.1016/j.jcis.2020.04.113.
(8) Liu, T. H.; Baek, D.; Kim, J. S.; Joo, S. W.; Lim, J. K. Green Synthesis of Silver Nanoparticles with Size Distribution Depending on Reducing Species in Glycerol at Ambient pH and Temperatures. *ACS Omega* **2020**, *5* (26), 16246-16254. DOI: 10.1021/acsomega.0c02066.
(9) Titkov, A. I.; Borisenko, T. A.; Logutenko, O. A.; Vorobyev, A. M.; Gerasimov, E. Y.; Shashkov, M. V. Tailoring Morphology and Size of Silver Nanoparticles Prepared by Polyol Method by Changing the Structure of Silver Carboxylate Precursor. *ChemistrySelect* **2023**, *8* (47). DOI: 10.1002/slct.202303693.
(10) Wolf, J. B.; Stawski, T. M.; Smales, G. J.; Thunemann, A. F.; Emmerling, F. Towards automation of the polyol process for the synthesis of silver nanoparticles. *Scientific Reports* **2022**, *12* (1), 5769. DOI: 10.1038/s41598-022-09774-w.
(11) Quinson, J. Questioning Established Protocols for New and More Sustainable Syntheses of Nanomaterials. *Langmuir* **2025**, *41* (50), 33669-33676. DOI: 10.1021/acs.langmuir.5c05054.
(12) Akhtar, M. S.; Panwar, J.; Yun, Y. S. Biogenic Synthesis of Metallic Nanoparticles by Plant Extracts. *ACS Sustainable Chemistry & Engineering* **2013**, *1* (6), 591-602. DOI: 10.1021/sc300118u.
(13) Hasan, K. M. F.; Liu, X. Y.; Zhou, S. Q.; Horváth, P. G.; Bak, M.; Bejó, L.; Sipos, G.; Alpár, T. Functional silver nanoparticles synthesis from sustainable point of view: 2000 to 2023-A review on game changing materials. *Heliyon* **2022**, *8* (12), e12322. DOI: 10.1016/j.heliyon.2022.e12322.
(14) Charitidis, C. A.; Georgiou, P.; Koklioti, M. A.; Trompeta, A.-F.; Markakis, V. Manufacturing nanomaterials: from research to industry. *Manufacturing Review* **2014**, *1*, 11. DOI: 10.1051/mfreview/2014009.
(15) Freese, T.; Elzinga, N.; Heinemann, M.; Lerch, M. M.; Feringa, B. L. The relevance of sustainable laboratory practices. *RSC Sustainability* **2024**, *2*, 1300-1336. DOI: 10.1039/D4SU00056K.

(16) Vadakkan, K.; Rumjit, N. P.; Ngangbam, A. K.; Vijayanand, S.; Nedumpillil, N. K. Novel advancements in the sustainable green synthesis approach of silver nanoparticles (AgNPs) for antibacterial therapeutic applications. *Coordination Chemistry Reviews* **2024**, *499*, 215528. DOI: 10.1016/j.ccr.2023.215528.
(17) Rasmussen, D. R.; Nielsen, M. F.; Quinson, J. Room Temperature Surfactant-Free Synthesis of Gold Nanoparticles in Alkaline Ethylene Glycol. *Chemistry* **2023**, *5* (2), 900-911. DOI: 10.3390/chemistry5020061.
(18) Yan, S.; Wu, Z.; Yu, H.; Gong, Y.; Tan, Y.; Du, R.; Chen, W.; Xing, X.; Mo, G.; Chen, Z.; et al. Time-Resolved Small-Angle X-ray Scattering Study on the Growth Behavior of Silver Nanoparticles. *Journal of Physical Chemistry C*, **2014**, *118* (21), 11454-11463. DOI: 10.1021/jp502482c.
(19) Sarkar, A.; Kapoor, S.; Mukherjee, T. Reduction and aggregation of silver ions in *tert*-butanol. *Research on Chemical Intermediates* **2005**, *31* (9), 845-856. DOI: 10.1163/156856705774576191.
(20) Huang, Z. Y.; Mills, G.; Hajek, B. Spontaneous formation of silver particles in basic 2-propanol. *Journal of Physical Chemistry* **1993**, *97* (44), 11542-11550. DOI: 10.1021/j100146a031.
(21) Liz-Marzan, L. M.; LadoTourino, I. Reduction and stabilization of silver nanoparticles in ethanol by nonionic surfactants. *Langmuir* **1996**, *12* (15), 3585-3589. DOI: 10.1021/la951501e.
(22) Pal, A.; Shah, S.; Devi, S. Microwave-assisted synthesis of silver nanoparticles using ethanol as a reducing agent. *Materials Chemistry and Physics* **2009**, *114* (2-3), 530-532. DOI: 10.1016/j.matchemphys.2008.11.056.
(23) Sun, Y.; Xia, Y. Shape-controlled synthesis of gold and silver nanoparticles. *Science*, **2002**; 298, 2176-2179. DOI: 10.1126/science.1077229.
(24) Fievet, F.; Ammar-Merah, S.; Brayner, R.; Chau, F.; Giraud, M.; Mammeri, F.; Peron, J.; Piquemal, J. Y.; Sicard, L.; Viau, G. The polyol process: a unique method for easy access to metal nanoparticles with tailored sizes, shapes and compositions. *Chemical Society Reviews* **2018**, *47* (14), 5187-5233. DOI: 10.1039/c7cs00777a.
(25) Jacob, J. A.; Kapoor, S.; Biswas, N.; Mukherjee, T. Size tunable synthesis of silver nanoparticles in water-ethylene glycol mixtures. *Colloids and Surfaces a-Physicochemical and Engineering Aspects* **2007**, *301* (1-3), 329-334. DOI: 10.1016/j.colsurfa.2006.12.070.
(26) Skrabalak, S. E.; Wiley, B. J.; Kim, M.; Formo, E. V.; Xia, Y. N. On the polyol synthesis of silver nanostructures: Glycolaldehyde as a reducing agent. *Nano Letters* **2008**, *8* (7), 2077-2081. DOI: 10.1021/nl800910d.
(27) Lim, J. K.; Liu, T.; Park, J. S. Enhanced Reaction Rate via Different Mechanisms for the Synthesis of Silver Nanoparticles in Glycerol by Eco-friendly Manipulations. *Bulletin of the Korean Chemical Society* **2021**, *42* (6), 863-871. DOI: 10.1002/bkcs.12276.
(28) Slistan-Grijalva, A.; Herrera-Urbina, R.; Rivas-Silva, J.; Avalos-Borjia, M.; Castillón-Barraza, F.; Posada-Amarillas, A. Assessment of growth of silver nanoparticles synthesized from an ethylene glycol-silver nitrate-polyvinylpyrrolidone solution. *Physica E-Low-Dimensional Systems & Nanostructures* **2005**; 25, 438-448. DOI: 10.1016/j.physe.2004.07.010.
(29) Sarkar, A.; Kapoor, S.; Mukherjee, T. Synthesis and characterisation of silver nanoparticles in viscous solvents and its transfer into non-polar solvents. *Research on Chemical Intermediates* **2010**, *36* (4), 411-421. DOI: 10.1007/s11164-010-0151-4.
(30) Gomes, J. F.; Garcia, A. C.; Ferreira, E. B.; Pires, C.; Oliveira, V. L.; Tremiliosi-Filho, G.; Gasparotto, L. H. S. New insights into the formation mechanism of Ag, Au and AgAu nanoparticles in aqueous alkaline media: alkoxides from alcohols, aldehydes and ketones as universal reducing agents. *Physical Chemistry Chemical Physics* **2015**, *17* (33), 21683-21693. DOI: 10.1039/c5cp02155c.
(31) Chen, S. Y.; Drehmel, J. R.; Penn, R. L. Facile Synthesis of Monodispersed Ag NPs in Ethylene Glycol Using Mixed Capping Agents. *ACS Omega* **2020**, *5* (11), 6069-6073. DOI: 10.1021/acsomega.9b04492.

(32) Cargnello, M.; Chen, C.; Diroll, B. T.; Doan-Nguyen, V. V. T.; Gorte, R. J.; Murray, C. B. Efficient Removal of Organic Ligands from Supported Nanocrystals by Fast Thermal Annealing Enables Catalytic Studies on Well-Defined Active Phases. *Journal of the American Chemical Society* **2015**, *137* (21), 6906-6911. DOI: 10.1021/jacs.5b03333.
(33) Pinto, V.; Ferreira, M.; Silva, R.; Santos, H.; Silva, F.; Pereira, C. Long time effect on the stability of silver nanoparticles in aqueous medium: Effect of the synthesis and storage conditions. *Colloids and Surfaces A-Physicochemical And Engineering Aspects* **2010**, *364*, 19-25. DOI: 10.1016/j.colsurfa.2010.04.015.
(34) Izak-Nau, E.; Huk, A.; Reidy, B.; Uggerud, H.; Vadset, M.; Eiden, S.; Voetz, M.; Himly, M.; Duschl, A.; Dusinska, M.; et al. Impact of storage conditions and storage time on silver nanoparticles' physicochemical properties and implications for their biological effects. *RSC Advances*, **2015**, 5, 84172-84185. DOI: 10.1039/C5RA10187E.
(35) Quinson, J.; Neumann, S.; Wannmacher, T.; Kacenauskaite, L.; Inaba, M.; Bucher, J.; Bizzotto, F.; Simonsen, S. B.; Kuhn, L. T.; Bujak, D.; et al. Colloids for Catalysts: A Concept for the Preparation of Superior Catalysts of Industrial Relevance. *Angewandte Chemie-International Edition* **2018**, *57* (38), 12338-12341. DOI: 10.1002/anie.201807450.
(36) Quinson, J.; Nielsen, T. M.; Escudero-Escribano, M.; Jensen, K. M. Ø. Room temperature syntheses of surfactant-free colloidal gold nanoparticles: the benefits of mono-alcohols over polyols as reducing agents for electrocatalysis. *Colloids and Surfaces A: Physicochemical and Engineering Aspects* **2023**, 675, 131853. DOI: 10.1016/j.colsurfa.2023.131853.
(37) Mathiesen, J. K.; Quinson, J.; Blaseio, S.; Kjær, E. T. S.; Dworzak, A.; Cooper, S.; Pedersen, J. K.; Wang, B.; Bizzotto, F.; Johanna, S.; et al. Chemical insights on the formation of colloidal iridium nanoparticles from in situ X-ray total scattering: Influence of precursors and cations on the reaction pathway. *Journal of the American Chemical Society* **2023**, *145* (3), 1769–1782. DOI: 10.1021/jacs.2c10814.
(38) Rasmussen, D. R.; Lock, N.; Quinson, J. Lights on the synthesis of surfactant-free colloidal gold nanoparticles in alkaline mixtures of alcohols and water. *ChemSusChem* **2025**, *18* (3), e202400763. DOI: 10.1002/cssc.202400763.
(39) Quinson, J. Influence of the alcohol and water grades on surfactant-free colloidal syntheses of gold nanoparticles in alkaline water-alcohol mixtures. *Gold Bulletin* **2024**, 57, 27-31. DOI: 10.1007/s13404-024-00345-7.
(40) Quinson, J.; Kunz, S.; Arenz, M. Surfactant-free colloidal syntheses of precious metal nanoparticles for improved catalysts. *ACS Catalysis* **2023**, *13* (7), 4903–4937. DOI: 10.1021/acscatal.2c05998.
(41) Han, J.; Chen, Y. M.; Nie, X. B. Environmentally Benign and Large-Scale Synthesis of Monodisperse Oleate-Protected Silver Nanoparticles in Ethanol. *Journal of Cluster Science* **2021**, *32* (4), 899-905. DOI: 10.1007/s10876-020-01852-1.
(42) Garcia, A.; Gasparotto, L.; Gomes, J.; Tremiliosi-Filho, G. Straightforward Synthesis of Carbon-Supported Ag Nanoparticles and Their Application for the Oxygen Reduction Reaction. *Electrocatalysis* **2012**; 3, 147-152. DOI: 10.1007/s12678-012-0096-z.
(43) Garcia, A. G.; Lopes, P. P.; Gomes, J. F.; Pires, C.; Ferreira, E. B.; Lucena, R. G. M.; Gasparotto, L. H. S.; Tremiliosi-Filho, G. Eco-friendly synthesis of bimetallic AuAg nanoparticles. *New Journal of Chemistry* **2014**, *38* (7), 2865-2873. DOI: 10.1039/c4nj00041b.
(44) Munir, I.; Yesiloz, G. Novel Size-Tunable and Straightforward Ultra-Small Nanoparticle Synthesis in a Varying Concentration Range of Glycerol as a Green Reducing Solvent. *ACS Omega* **2023**, *8* (31), 28456-28466. DOI: 10.1021/acsomega.3c02697.
(45) Jæger, F.; Pedersen, A. A.; Wacherhausen, P. S.; Smolska, A.; Quinson, J. Surfactant-free gold nanoparticles synthesized in alkaline water-ethanol mixtures: leveraging lower grade chemicals for size control of active nanocatalysts. *RSC Sustainability* **2025**, 3, 2870-2875. DOI: 10.1039/D5SU00213C.
(46) Larm, N. E.; Bhawawet, N.; Thon, J. A.; Baker, G. A. Best practices for reporting nanocatalytic performance: lessons learned from nitroarene reduction as a model reaction. *New Journal of Chemistry* **2019**, *43* (46), 17932-17936. DOI: 10.1039/c9nj01745c.

(47) Mourdikoudis, S.; Pallares, R. M.; Thanh, N. T. K. Characterization techniques for nanoparticles: comparison and complementarity upon studying nanoparticle properties. *Nanoscale* **2018**, *10* (27), 12871-12934. DOI: 10.1039/c8nr02278j.
(48) Amirjani, A.; Firouzi, F.; Haghshenas, D. F. Predicting the Size of Silver Nanoparticles from Their Optical Properties. *Plasmonics* **2020**, *15* (4), 1077-1082. DOI: 10.1007/s11468-020-01121-x.
(49) Sileikaite, A.; Puiso, J.; Prosycevas, I.; Tamulevicius, S. Investigation of Silver Nanoparticles Formation Kinetics During Reduction of Silver Nitrate with Sodium Citrate. *Materials Science-Medziagotyra* **2009**, 15 (1), 21-27. DOI: 10.5755/j02.ms.26114.
(50) Klinavicius, T.; Khinevich, N.; Tamuleviciene, A.; Vidal, L.; Tamulevicius, S.; Tamulevicius, T. Deep Learning Methods for Colloidal Silver Nanoparticle Concentration and Size Distribution Determination from UV-Vis Extinction Spectra. *Journal of Physical Chemistry C* **2024**, *128* (23), 9662-9675. DOI: 10.1021/acs.jpcc.4c02459.
(51) Shume, W.; Murthy, H.; Zereffa, E. A Review on Synthesis and Characterization of Ag_2O Nanoparticles for Photocatalytic Applications. *Journal of Chemistry* **2020**, *2020*, 5039479. DOI: 10.1155/2020/5039479.
(52) Parveen, R.; Ullah, S.; Sgarbi, R.; Tremiliosi, G. One-pot ligand-free synthesis of gold nanoparticles: The role of glycerol as reducing-cum-stabilizing agent. *Colloids and Surfaces a-Physicochemical and Engineering Aspects* **2019**, *565*, 162-171. DOI: 10.1016/j.colsurfa.2019.01.005.
(53) Prat, D.; Wells, A.; Hayler, J.; Sneddon, H.; McElroy, C. R.; Abou-Shehada, S.; Dunn, P. J. CHEM21 selection guide of classical- and less classical-solvents. *Green Chemistry* **2016**, *18* (1), 288-296. DOI: 10.1039/c5gc01008j.
(54) Ozkar, S.; Finke, R. G. Nanoparticle Formation Kinetics and Mechanistic Studies Important to Mechanism-Based Particle-Size Control: Evidence for Ligand-Based Slowing of the Autocatalytic Surface Growth Step Plus Postulated Mechanisms. *Journal of Physical Chemistry C* **2019**, *123* (22), 14047-14057. DOI: 10.1021/acs.jpcc.9b03220.
(55) Polte, J.; Tuaev, X.; Wuithschick, M.; Fischer, A.; Thuenemann, A.; Rademann, K.; Kraehnert, R.; Emmerling, F. Formation Mechanism of Colloidal Silver Nanoparticles: Analogies and Differences to the Growth of Gold Nanoparticles. *ACS Nano* **2012**, 6, 5791-5802. DOI: 10.1021/nn301724z.
